# Environmental exposures in early-life and general health in childhood

**DOI:** 10.1186/s12940-023-01001-x

**Published:** 2023-07-21

**Authors:** Ines Amine, Alicia Guillien, Claire Philippat, Augusto Anguita-Ruiz, Maribel Casas, Montserrat de Castro, Audrius Dedele, Judith Garcia-Aymerich, Berit Granum, Regina Grazuleviciene, Barbara Heude, Line Småstuen Haug, Jordi Julvez, Mónica López-Vicente, Léa Maitre, Rosemary McEachan, Mark Nieuwenhuijsen, Nikos Stratakis, Marina Vafeiadi, John Wright, Tiffany Yang, Wen Lun Yuan, Xavier Basagaña, Rémy Slama, Martine Vrijheid, Valérie Siroux

**Affiliations:** 1 University Grenoble Alpes, Inserm U 1209, CNRS UMR 5309, Team of Environmental Epidemiology applied to Development and Respiratory Health, Institute for Advanced Biosciences, Grenoble, France; 2 Parc de Recerca Biomèdica de Barcelona (PRBB), ISGlobal-Instituto de Salud Global de Barcelona Campus MAR, 08003 Barcelona, Spain; 3 CIBEROBN, (Physiopathology of Obesity and Nutrition CB12/03/30038), Institute of Health Carlos III (ISCIII), 28029 Madrid, Spain; 4 Pompeu Fabra University (UPF), 08002 Barcelona, Spain; 5 Spanish Consortium for Research On Epidemiology and Public Health (CIBERESP), Av. Monforte de Lemos, 3-5. Pabellón 11, 28029 Madrid, Spain; 6 Epidemiology and Environmental Health Joint Research Unit, Foundation for the Promotion of Health and Biomedical Research in the Valencian Region, FISABIO-Public Health, FISABIO-Universitat Jaume I-Universitat de València, Av. Catalunya 21, 46020 Valencia, Spain; 7 Department of Environmental Science, Vytautas Magnus University, 44248 Kaunas, Lithuania; 8 Division for Climate and Environmental Health, Norwegian Institute of Public Health, 0213 Oslo, Norway; 9 Université Paris Cité and Université Sorbonne Paris Nord, Inserm, INRAE, Center for Research in Epidemiology and StatisticS (CRESS), 75004 Paris, France; 10 Clinical and Epidemiological Neuroscience (NeuroÈpia), Institut d’Investigació Sanitària Pere Virgili (IISPV), 43204 Reus, Spain; 11 Bradford Institute for Health Research, Bradford Teaching Hospitals NHS Foundation Trust, Bradford, UK; 12 Department of Social Medicine, School of Medicine, University of Crete, Heraklion, Crete, Greece; 13 Singapore Institute for Clinical Science, Agency for Science, Technology and Research (A*STAR), Singapore, Singapore

**Keywords:** Environment, Exposome, Cohort studies, Cardiometabolic risk factors, Neurodevelopment, Respiratory diseases, Pregnancy, Child, Multimorbidity, General health status

## Abstract

**Background:**

Early-life environmental exposures are suspected to be involved in the development of chronic diseases later in life. Most studies conducted so far considered single or few exposures and single-health parameter. Our study aimed to identify a childhood general health score and assess its association with a wide range of pre- and post-natal environmental exposures.

**Methods:**

The analysis is based on 870 children (6–12 years) from six European birth cohorts participating in the Human Early-Life Exposome project. A total of 53 prenatal and 105 childhood environmental factors were considered, including lifestyle, social, urban and chemical exposures. We built a general health score by averaging three sub-scores (cardiometabolic, respiratory/allergy and mental) built from 15 health parameters. By construct, a child with a low score has a low general health status. Penalized multivariable regression through Least Absolute Shrinkage and Selection Operator (LASSO) was fitted in order to identify exposures associated with the general health score.

**Findings:**

The results of LASSO show that a lower general health score was associated with maternal passive and active smoking during pregnancy and postnatal exposure to methylparaben, copper, indoor air pollutants, high intake of caffeinated drinks and few contacts with friends and family. Higher child’s general health score was associated with prenatal exposure to a bluespace near residency and postnatal exposures to pets, cobalt, high intakes of vegetables and more physical activity. Against our hypotheses, postnatal exposure to organochlorine compounds and perfluorooctanoate were associated with a higher child’s general health score.

**Conclusion:**

By using a general health score summarizing the child cardiometabolic, respiratory/allergy and mental health, this study reinforced previously suspected environmental factors associated with various child health parameters (e.g. tobacco, air pollutants) and identified new factors (e.g. pets, bluespace) warranting further investigations.

**Supplementary Information:**

The online version contains supplementary material available at 10.1186/s12940-023-01001-x.

## Introduction

It is recognized that the early-life period is particularly vulnerable to the influences of environmental factors, in particular the pregnancy period and the first years of life [1]. The concept of “exposome” is defined by all the exposures that a human being undergoes since conception [2], ranging from air pollution to chemical pollutants, the social environment etc. In recent years, an increased number of studies based on the exposome approach identified the main environmental threats for specific health parameters [3–5] or for a specific health domain [6–8]. However, this traditional approach of investigating exposures associated with single health parameter is limited. One main limit is that it fails to recognize the whole system nature of multiple interactive exposures that shape multiple health outcomes.

In addition to the outcome-wide approach previously proposed [9], an approach based on a general health indicator is relevant. While the outcome-wide approach assesses the impact of exposures on several health outcomes considered independently, a general health score aims to cover multiple health domains (*e.g.* cardiometabolic, respiratory/allergy and mental health) in a single indicator. This approach is based on the assumption that mental, cardiometabolic and respiratory outcomes partly share some biological pathways that are affected by environmental factors. This assumption is supported by the identification of pleiotropic genes and evidences for shared influence of major regulating systems such as inflammation and oxidative stress between these various health outcomes [10–13]. Pointing out early-life exposures associated with multiple health domains in children is needed to prioritise public health messages but also to prevent multimorbidity, i.e. the coexistence of several conditions in the same individual. This approach may lead to the identification of new environmental health risk factors as some exposure affects in a low-grade manner multiple health outcomes. As far as we know, few general health indicators exist out of the spectrum of questionnaire on quality of life related to self-perceived general health (*e.g*. the Child Health Questionnaire [14]) and no study has sought for environmental factors affecting a general health indicator in children.

This project aimed to compute a general health score and assess its association with multiple prenatal and postnatal environmental factors, in the large European Human Early-Life Exposome (HELIX) cohort [15, 16]. Our main hypothesis is that this approach can reinforce the significance of some suspected environmental factors and identify new risk factors simultaneously affecting various health parameters.

## Materials and methods

### Study population

This study is based from the HELIX project, which includes six existing population-based birth cohorts: Born in Bradford (BiB, UK) [17], Étude des Déterminants pré et postnatals du développement et de la santé de l’Enfant (EDEN, France) [18], Infancia y Medio Ambiente (INMA, Spain) [19], Kaunas Cohort (KANC, Lithuania) [20], The Norwegian Mother, Father and Child Cohort Study (MoBa, Norway) [21], and Mother–Child Cohort (RHEA, Greece) [22]. Around 32,000 mothers were recruited during pregnancy (2003–2009), from which 1,301 mother–child pairs were followed-up when the child was 6–11 years old (2014–2015). Standardized protocols were used to collect biological samples and questionnaire data, conduct health examinations and characterise a large range of exposures. The present study included 870 mother–child pairs for which data was available to build the general health score (see more details in the following part).

### Health data: cardiometabolic, respiratory/allergy and mental health

Fifteen health parameters were considered for this study, covering the cardiometabolic, respiratory and mental health, as listed in Table 1 (see more details in eMethods 1). The cardiometabolic parameters considered were the child blood pressure (diastolic and systolic), the waist circumference, lipids (high-density lipoprotein (HDL) cholesterol and triglycerides) and insulin levels. The first two parameters were measured by medical staff, and the last two were obtained through blood and serum, respectively. The respiratory and allergy-related health was assessed by spirometry (Forced Expiratory Volume in one second (FEV_1_)) and by a questionnaire adapted from the International Study on Asthma and Allergy in Childhood (ISAAC) [23] including doctor-diagnosed asthma, food allergies, eczema, as well as rhinitis symptoms [3, 7]. The cognitive and behavioural parameters considered were the measured fluid intelligence (Raven Colour Progressive Matrix™), an index regarding symptoms of Attention Disabilities and Hyperactivity Disorders (ADHD) (Conner’s rating scales of 27 items) and internalizing and externalizing scores (99-item Child Behaviour Checklist (CBCL) [6, 8]. All these health parameters were measured at the Helix follow-up when the child was between 6–11 years old (see eTable 1).

**Table 1:** List of health parameters studied

Cardiometabolic health	Respiratory health and allergies	Mental health and cognition
- Lipids (HDL cholesterol, triglycerides) - Blood pressure (diastolic, systolic) - Circumference of the waist - Insulin levels	**-** Lung function (FEV_1_% pred) **-** Asthma **-** Food allergies **-** Eczema **-** Rhinitis	**-** ADHD index (Conners) **-** Internalizing and externalizing indexes (CBCL) **-** Test of fluid intelligence (Raven)

*Abbreviations HDL* High Density Lipoprotein, *FEV1* Forced Expiratory Volume in 1 s, *ADHD* Attention Deficit Hyperactivity Disorders, *CBCL* Child Behaviour Checklist

From the whole HELIX population (*n* = 1,301), at least one health parameter was missing for 11.5% (*n* = 150) of children regarding cardiometabolic parameters, for 32.4% (*n* = 294) of children regarding respiratory and allergic parameters (mostly due to FEV_1_), and for 0.8% (*n* = 23) of children regarding mental parameters. Children with all fifteen health parameters were included, leading to the inclusion of 870 mother–child pairs.

### Characterisation of the exposome

A wide range of environmental exposures was assessed in each mother–child pair, covering 21 families of exposures, with 53 prenatal and 105 postnatal exposures, as detailed in Table 2 (see also previous Helix papers [16, 24, 25]). Briefly, outdoor exposures were assessed based on remote and spatial sensing data from a geographical information system (see eMethods 2). Factors regarding the lifestyle were collected by questionnaire and included smoking habits of the mother, food intakes, the social environment (pregnancy and childhood), physical activity, sleep and the presence of pets (childhood) (see eMethods 3). Biomarkers of chemical compounds were measured through biological samples (mostly serum and urine, as detailed in eTable 2) during pregnancy and childhood (see eMethods 4). Collection time points for prenatal exposures are given in eTables 3 and 4.

**Table 2:** List of all environmental exposures studied

Type of exposure	Exposures pregnancy (N)	Exposures measured during pregnancy	Exposures childhood (N)	Exposures measured during childhood (6–11 years)
URBAN EXPOSOME				
Outdoor air pollution	4	NO_2_, PM_10._ PM_2.5_, PM_2.5_ absorbance	7	NO_2_ (home, school), PM_10_ (home), PM_2.5_ (home, school), PM_2.5_ absorbance (home, school)
Indoor air pollution	0		5	NO_2_, PM_2.5_, PM absorbance, Benzene, TEX
Meteorology	3	Temperature, humidity, pressure	3	Temperature, humidity, UV-vit D
Surrounding natural spaces	3	NDVI, presence of a major greenspace and bluespace	6	NDVI (home, school), presence of a major greenspace (home, school) and bluespace (home, school)
Built environment	7	Population density, building density, street connectivity, accessibility, facility richness, walkability, land use index	16	Population density (home, school), building density (home, school), street connectivity (home, school), accessibility (home, school), facility richness (home, school), facility density (home, school), walkability (home, school), land use index (home, school)
Road traffic	3	Traffic load on all roads, traffic density on nearest road, inverse distance to nearest road	6	Traffic load on all roads (home, school), traffic density on nearest road (home, school), inverse distance to nearest road (home, school)
Water DBPs	3	THMs, brominated THMs, chloroform	0	
LIFESTYLE				
Tobacco	2	Active and passive smoking	2	Child exposure to smoke (ETS) and parental smoking
Diet	3	Specific food intake: meat, fish, cereal	20	KIDMED score, fast-food visits, organic food, specific food intake: sweets, meat, processed meat, fish, yogurt, sodas, bread, breakfast cereals, potatoes, vegetables, dairy, fruits, cereal, bakery products, lipids, caffeinated drinks
Sleep	0		1	Average sleep
Physical activity	0		2	Moderate/vigorous activity, sedentary time
Allergens	0		1	Pet
Socio-economic	1	House crowding	3	Family Affluence Score (FAS), family contact, participation in organizations
CHEMICAL EXPOSOME				
Perfluoroalkyl substances (PFASs)	5	PFHxS, PFOS, PFOA, PFNA, PFUnDA	4	PFHxS, PFOS, PFOA, PFNA
Brominated compounds (PBDEs)	0		1	PBDE 47
Metal and essential elements	1	Hg	10	Hg, Cd, Pb, Cs, Cu, Mn, Co, Mo, Tl, Se
Phthalates	6	MEP, MiBP, MnBP, MBzP, DEHP^b^, DiNP^c^	6	MEP, MiBP, MnBP, MBzP, DEHP^b^, DiNP^c^
Phenols	5	PRPA, BUPA, BPA, OXBE, TCS	6	MEPA, ETPA, BUPA, BPA, OXBE, TCS
Organochlorine pesticides (OCs)	3	PCB^a^, DDE, HCB	3	PCB^a^, DDE, HCB
Organophosphate (OP) metabolites	3	DMP, DMTP, DEP	2	DMTP, DEP
Tobacco	1	Cotinine	1	Cotinine

*BPA* Bisphenol-A, *BUPA* Butyl-paraben, *Cd* Cadmium, *Co* cobalt, *Cu* copper, *DDE* Dichlorodiphenyldichloroethylene, *DEHP* Di EthylhexylPhthalate, *DEP* Diethyl phosphate, *DMP* Dimethyl phosphate, *DMTP* Dimethyl thiophosphate, *ETPA* Ethyl-paraben, *HCB* Hexachlorobenzene, *Hg* Mercury, *KIDMED* Mediterranean diet in children, *MBzP* Mono benzyl phthalate, *DiNP* Diisononyl phthalate, *MEP* Monoethyl phthalate, *MEPA* Methyl-paraben, *Mo* Molybdenum, *MiBP* Mono-iso butyl phthalate, *NDVI* Normalized difference vegetation index, *NO*_*2*_ Nitrogen dioxide, *OCs* organochlorine compound, *OP* organophosphate pesticide, *OXBE* Oxybenzone, *Pb* Plomb, *PBDE* Polybrominated diphenyl ether, *PCB* Polychlorobiphenyls, *PFASs* per- and polyfluoroalkyl substance, *PFHxS* Perfluorohexane sulfonate, *PFNA* Perfluorononanoate, *PFOA* Perfluorooctanoate, *PFOS* Perfluorooctane sulfonate, *PFUNDA* Perfluoroundecanoate, *PM* Particulate matter, *PRPA* Propyl-paraben, *Se* Selenium, *Tl* Thalium, *TRCS* Triclosan, *UV-Vit. D* Vitamin-D dose from ultraviolet

^a^Pregnancy: PCB138 + PCB153 + PCB 180. Childhood: PCB118 + PCB 138 + PCB153 + PCB170 + PCB180

^b^DEHP: molar sum of MEHP, MEHHP, MEOHP and MECPP

^c^DiNP: molar sum of oxo-MiNP and oh-MiNP

### Covariates

Covariates used for the prenatal analyses included cohort, child age and sex, maternal age, highest parental education (primary, secondary or higher education), parental country of birth (none, one or both parents born in the cohort country), pre-pregnancy body mass index (BMI) and season of birth (winter, spring, summer or autumn). Regarding postnatal analyses, breastfeeding duration (< 11 weeks, 11–35 weeks, > 35 weeks) was added to the set of covariates.

### Creation of the general health score

The general health score averaged three sub-scores, each representing a specific health domain (cardiometabolic, respiratory/allergy and mental health). Beforehand, continuous health parameters were transformed in z-scores, using Generalize Additive Model for Location, Scale and Shape (GAMLSS) [26] to standardize on covariates (mostly age and sex, see eTables 5 and 6) and approach normality. The health parameters were not adjusted on each cohort in order to keep the between-cohort variability of the general health status for descriptive purposes. As used previously in the Helix population, the cardiometabolic sub-score was defined as (-z waist circumference) + (- z insulin) + (z HDL cholesterol – z triglycerides)/2 + (-z systolic BP – z diastolic BP)/2 [27, 28]. Following the approach of Eisenmann [29] the respiratory/allergy sub-score and the mental sub-score were defined as the first principal component of a multiple factorial analysis (see eFigures 1–2 and eTable 7–8). All of the three sub-scores were built such that a higher score means the child is in better health (see eMethods 5). The three sub-scores were scaled and aggregated into a single general health score by taking their mean. By construct, the general health score is low for children with conjointly low-to-moderate cardiometabolic, respiratory/allergy and mental health in children, as well as for children highly affected in one health domain while no or moderately affected for the other two.

### Strategy of analysis for the exposome-health association

For all exposures and covariates, the optimal transformation to approach normality was applied (see eTable 9), which is necessary for following steps including imputation and penalized regression models. Imputation of the missing values on exposures and covariates was done using the method of chained equations [30]. (see more details in eMethods 6). It generated 20 imputed datasets, used in the statistical analyses with the Rubin’s rule. After imputation, continuous exposures were centred and standardized by the interquartile range (IQR).

The exposure-general health score association study was performed separately for the prenatal and postnatal exposures using the Least Absolute Shrinkage and Selection Operator (LASSO) as the main analysis [31]. This penalized regression model considers all exposures and covariates simultaneously and selects the best predictors of the outcome (note that covariates were forced in the model). Optimization of the penalizing parameter $\lambda$ was performed by minimizing the mean cross-validated error on each of the 20 imputed dataset. The exposures selected for at least 50% of LASSO models (10 imputed datasets out of the 20) were used as the final set of exposures [32]. The main models consisted in two multivariable linear regressions (one prenatal and one postnatal) considering all the selected exposures, after removing all exposures with *p*-value higher than 10%. More details on the strategy of analysis can be found in eMethods 7.

As secondary analyses, an exposome-wide association study (ExWAS) was conducted. It considered each exposure in separate linear regression models [33], adjusted on the same covariates, and corrected for multiple hypothesis testing (adapted from Li [34]). Moreover, some specific hypotheses were tested: 1) For organochlorine compounds (OCs), the associations found were stratified on the terciles of the BMI because OCs are known to accumulate in fat; 2) for PFASs, the associations were adjusted on fish consumption as a correlation between PFASs and fish consumption has been noticed in the Helix population [35] 3) the final multivariable models were stratified on sex to address a potential gender-specific association; 4) the final multivariable models were stratified on cohort to address the robustness of the findings to the multicentre study design; 5) the linearity of the associations was tested using a Generalized Additive Model (GAM) with smooth functions for all selected exposures. In addition, a sensitivity analysis to assess the robustness to extreme values was conducted by fitting the multivariable model after excluding the 2% lowest and 2% highest values for the general health score (*n* = 836).

**Fig. 1:**
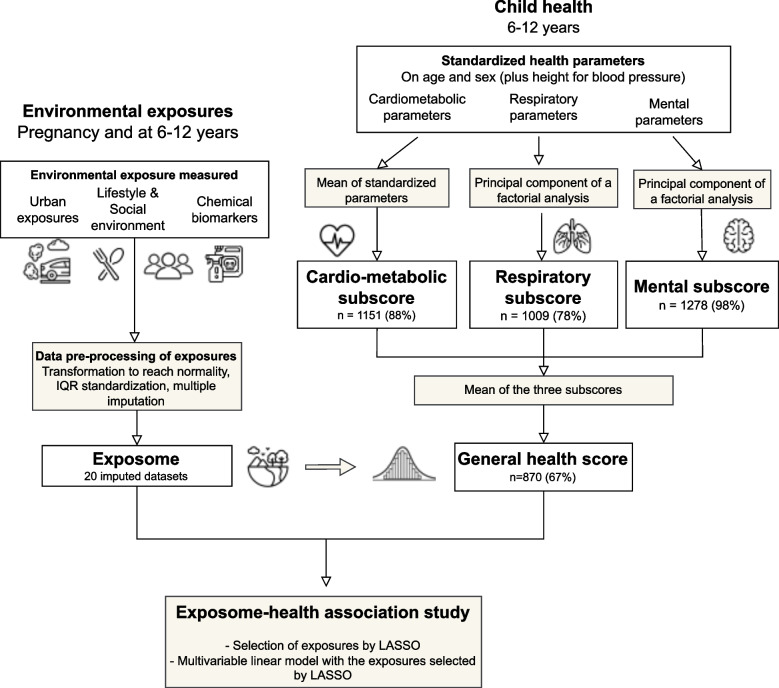
Main steps of the analysis

For better comparability across exposures, estimates were expressed as an increase in interquartile range of the transformed exposure (continuous exposures). Significance level was defined as 5% for all statistical tests. Analyses were done with R version 4.2.1, using the packages *mice*, *gamlss*, *FactoMineR*, *psych* and *glmnet*. The main steps in the analysis are summarized in Fig. 1.

## Results

### Description of the population

The study population, aged between 5.4 and 12.0 years old (median = 8.1 years old) at the HELIX follow-up was 47% girls (Table 3). At birth, mothers were on average 31 years old and about half of them (51%) had a high degree of education. Tables describing the exposures and health parameters (including percent of missing data) during pregnancy and childhood are available in the supplementary materials (eTables 10, 11, 12 and 13).

**Table 3:** Description of the study population before imputation

Variable name	N (%)	Min	Q1	Median	Q3	Max
Maternal age at birth	863 (99.2)	17	27.7	31.0	34	43.3
Pre-pregnancy BMI	855 (98.3)	16.2	21.3	23.2	27	43
Child age at the follow up	870 (100)	5.4	6.52	8.1	8.9	12
		**Category**			**n (%)**	
Cohort	870 (100)	BiB			134 (15.4)	
		EDEN			112 (12.9)	
		INMA			160 (18.4)	
		KANC			129 (14.8)	
		MoBA			193 (22.2)	
		RHEA			142 (16.3)	
Highest parental education	858 (99)	Primary			92 (10.7)	
		Secondary			266 (31.0)	
		Higher			500 (58.3)	
Child sex	870 (100)	Girl			411 (47.2)	
		Boy			459 (52.8)	
Child weight status (IOTF)	870 (100)	Underweight/Normal			700 (80.5)	
		Overweight			119 (13.7)	
		Obese			51 (5.9)	
Child asthma (ever)	870 (100)	No			767 (88.2)	
		Yes			103 (11.8)	
ADHD score	870 (100)	Not at risk for ADHD (≤ 16)			793 (91.1)	
		At risk for ADHD (≥ 17)			77 (8.9)	

Population: study population from the HELIX subcohort, *n* = 870 children

*Abbreviations*: *ADHD* Attention Deficit Hyperactivity Disorders, *BMI* Body Mass Index, *BiB* Born in Bradford, *EDEN* Étude des Déterminants pré et postnatals du développement et de la santé de l’Enfant, *INMA* Infancia y Medio Ambiente, *IOTF* International Obesity Task Force, *KANC* Kaunus Cohort, *MoBa* The Norwegian Mother, Father and Child Cohort Study, *RHEA* Mother–Child Cohort in Crete

### Description of the general health score

The cardiometabolic, respiratory/allergy and mental sub-scores ranged between -3.20 and 3.10, -4.53 and 3.18, and -2.89 and 2.76, respectively. The three sub-scores were poorly correlated (eTable 14), with more details and descriptions in the supplementary (eFigure 3, eTable 7– 8).

**Fig. 2:**
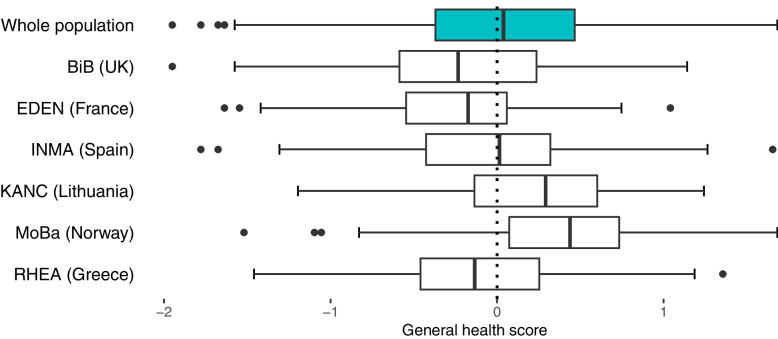
Distribution of the general health score by cohort Boxplot showing the distributions of the built general health score in the whole population and in each cohort. Population: study population from the HELIX subcohort (*n* = 870). Accronyms: BiB: Born in Bradford, EDEN: Étude des Déterminants pré et postnatals du développement et de la santé de l’Enfant, INMA: Infancia y Medio Ambiente, KANC: Kaunus Cohort, MoBa: The Norwegian Mother, Father and Child Cohort Study, RHEA: Mother–Child Cohort in Crete

The general health score, calculated as the mean of the three sub-scores, had a normal distribution (Shapiro test *p*-value = 0.21) with a mean (sd) of 0.03 (0.60). The median general health score varied among cohorts, with the lowest in BiB (median = -0.21) and the highest in MoBA (median = 0.42), as shown in Fig. 2. The general health score increased with parental education, breast-feeding duration and maternal age, and decreased with pre-pregnancy BMI (eTable 15). The joint distributions of the sub-scores, key health parameters and the general health score are presented in the supplementary (eTable 16).

### Which exposures were associated with the general health score?

**Fig. 3:**
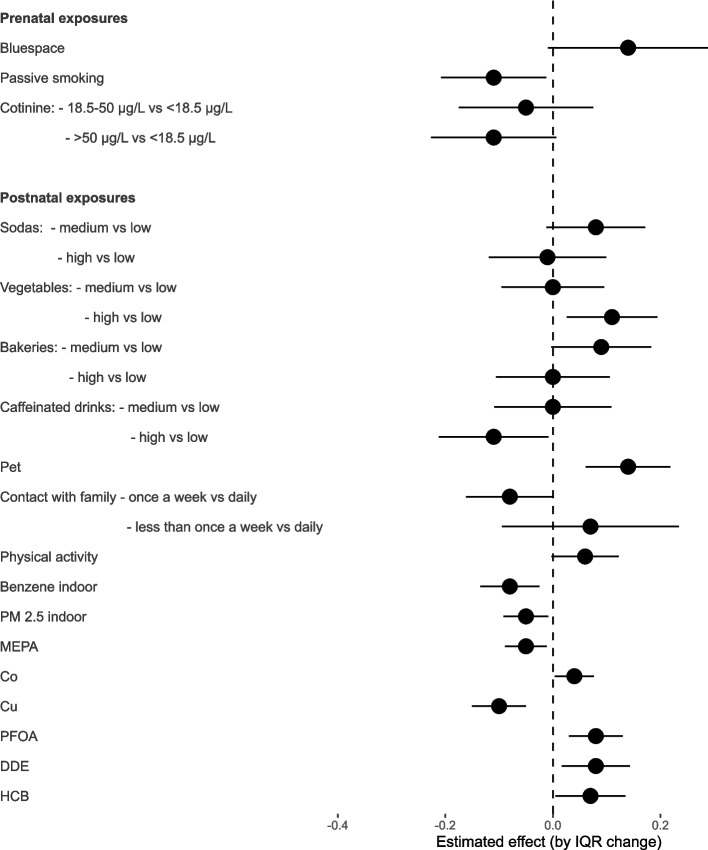
Results of the final multivariable models Population: *n* = 870 children from the HELIX subcohort. Method: multivariable models between the general health score and the exposures selected by LASSO for 50% of models, plus the covariates, separately for prenatal and postnatal exposures. All exposures with a *p*-value > 10% were removed one by one from the final model. Covariates: cohort, child age, maternal education and age, parental country of birth, season of birth, pre-pregnancy BMI, plus the breastfeeding duration for postnatal exposures only. Acronyms: Co: Cobalt, Cu: Copper, DDE: Dichlorodiphenyldichloroethylene, HCB: Hexachlorobenzene, MEPA: Methyl-paraben, PFOA: Perfluorooctanoate, PM: Particulate matter

Three exposures during pregnancy were selected by LASSO: maternal passive smoking (assessed by questionnaire), maternal active smoking (assessed by cotinine levels) and the presence of a bluespace near residency (Fig. 3 and Table 4). In the multivariable model, maternal passive smoking remained significantly associated with a poorer general health score. Although not significant, higher levels of cotinine (> 50 µg/L vs < 18.5 µg/L) were association with a poorer score (*p*-value = 0.09) and the presence of a bluespace was associated with a better score (*p*-value = 0.07).

**Table 4:** Results from LASSO Exposures selected in the final model

**Exposures**	**Transformation**^a^	**LASSO**	**Multivariable model from LASSO results**	
		% of selection among the 20 imputed datasets	Estimate change by IQR [95% CI]	*p*-value^b^
**During pregnancy**				
Presence of a bluespace	-^c^	60%	0.13 [-0.02; 0.28]	0.08
Passive smoking (yes)	-	90%	-0.11 [-0.21; -0.01]	0.02
Cotinine levels	-	65%		
< *18.5 µg/L*			*reference*	
*18.5–50 µg/L (passive smoking)*			-0.05 [-0.18; 0.08]	0.43
> *50 µg/L (active smoking)*			-0.1 [-0.22; 0.01]	0.09
**During childhood**				
Sodas	-	100%		
*Low (*< *1 time/month)*			*reference*	
*Medium (1 time/month – 1 time/week)*			0.09 [0.00; 0.18]	0.06
*High (*> *1 time/week)*			-0.01 [-0.12; 0.10]	0.85
Vegetables	-	100%		
*Low (*< *6 times/week)*			*reference*	
*Medium (6–9 times/week)*			0.00 [-0.09; 0.1]	0.94
*High (*> *9 times/week)*			0.10 [0.02; 0.19]	0.02
Bakery products	-	100%		
*Low (*< *2 times/week)*			*reference*	
*Medium (2–6 times/week)*			0.10 [0.00; 0.19]	0.04
*High (*> *6 times/week)*			0.00 [-0.11; 0.10]	0.93
Caffeinated drink	-	100%		
*Low (Never)*			*reference*	
*Medium (1 time/month)*			0.00 [-0.11; 0.10]	0.93
*High (*> *1 time/month)*			-0.10 [-0.21; 0.00]	0.05
Pet (yes)	-	100%	0.14 [0.06; 0.22]	< 0.01
Contact with family and friends	-	100%		
*Daily*			*reference*	
*Once a week*			-0.08 [-0.16; 0.00]	0.05
*Less than once a week*			0.06 [-0.10; 0.23]	0.44
Physical activity		95%	0.06 [0.00; 0.12]	0.06
Benzene indoor	Log	100%	-0.08 [-0.14; -0.03]	< 0.01
PM_2.5_ indoor	Log	100%	-0.05 [-0.10; -0.01]	0.01
MEPA	Log	100%	-0.05 [-0.09; -0.01]	0.01
Co	Log	100%	0.04 [0.01; 0.08]	0.02
Cu	Log	100%	-0.10 [-0.15; -0.05]	< 0.01
PFOA	Log	100%	0.08 [0.03; 0.13]	< 0.01
DDE	Log	100%	0.08 [0.01; 0.14]	0.02
HCB	Log	100%	0.07 [0.00; 0.13]	0.04

Population: *n* = 870 children from the HELIX subcohort. Methods: first, penalized linear regression model was applied, with regularization parameter (lambda) optimized with tenfold cross validation. Then, all exposures selected for at least 10 imputed datasets (50% of selection) were included in a multivariable linear regression adjusted for the covariates. All exposures with a *p*-value > 10% were removed one by one from the final model. Covariates: cohort, child age, maternal education and age, parental country of birth, season of birth, pre-pregnancy BMI, plus the breastfeeding duration for postnatal exposures only

*Co* cobalt, *Cu* Copper, *DDE* Dichlorodiphenyldichloroethylene, *HCB* Hexachlorobenzene, *MEPA* Methyl-paraben, *PFOA*: Perfluorooctanoate, *PM*: Particulate matter

^a^The transformation was applied before the standardisation on the interquartile range (IQR). This last transformation consisted in removing the mean and dividing by the IQR

^b^t-test on the estimated coefficients, based on the Rubin’s rule

^c^ “- “ means that no transformation was applied, mainly because the variable was categorical

Regarding the exposures during childhood, a total of 23 variables was selected by LASSO and 16 of them were kept in the final multivariable model (*p*-value ≤ 10%) (Fig. 3 and Table 4). High intakes of caffeinated drinks (compared to low intakes), indoor levels of benzene and PM_2.5_, exposure to methylparaben and copper were significantly associated with a poorer general health score. A non-significant association (*p*-value = 0.07) was observed between less frequent contact with family and friends (once a week vs daily) and a poorer health score. On the other hand, intakes of vegetables (high vs low intake), owning a pet, physical activity, cobalt, exposure to perfluorooctanoate (PFOA), dichlorodiphenyldichloroethylene (DDE) and hexachlorobenzene (HCB) were significantly associated with a better score. Suggestive associations (0.05 < *p* < 0.10) were observed between medium intakes of sodas and bakery products and a better score.

The ExWAS approach led to similar results than LASSO, highlighting significant associations of the general health score with postnatal exposures to pets, diet, metals, indoor air pollutants, OCs and PFOA. No association with prenatal exposure remained significant after correcting on multiple testing. All estimations are available in the supplementary materials (eTables 17 and 18).

After stratifying on the terciles of BMI, higher DDE exposure was associated with a better general health score in the low BMI group, but tended to be associated with a poorer score in the high BMI group (see eFigure 4). Adding fish consumption as a confounder variable did not change the results estimated for PFOA (see eTable 19). Results of the multivariable models stratified by sex showed overall similar results in boys and girls (eFigures 5 and 6), although boys-specific associations were observed for postnatal exposures to indoor benzene and HCB. When stratifying on cohorts, results were overall consistent (see eFigures 7 and 8) although some differences were observed for postnatal exposure to copper, DDE, contact with family and friends and intake of bakery products. The results of GAM did not invalidate the assumption of linearity for most exposures at the exception of child HCB (see eFigure 9). The general health score first increased with child HCB for “low” HCB levels, but was constant for “moderate-to-high” HCB level. In the sensitivity analysis where extreme values of the general health score were removed, the magnitude of the associations remained similar (see eFigure 10).

## Discussion

This novel study intended to approach the complexity of multiple exposures impacting multiple health parameters by assessing the association between a wide range of pre- and post-natal exposures and a general health score in children. Three prenatal and fourteen postnatal exposures associated with the child’s general health score were identified. Environmental factors already suspected of being associated with some child’s health parameters were reinforced, such as maternal smoking exposures during pregnancy, a healthy lifestyle, indoor air pollutants and parabens. In addition, our findings pinpoint new environmental factors associated with child’s health, particularly the presence of a nearby bluespace during pregnancy and pets during childhood were associated with a better child’s general health score.

### Interpretation of the results and comparison with the literature 

Previously suspected environmental factors were identified in this study, in particular tobacco, diet, the social environment, metals and parabens. While tobacco, caffeinated drinks, indoor air pollutants, parabens and few contacts with family were associated with a poorer general health score, a healthy diet was associated with a better general health score. Interestingly, these six families of exposures have been highlighted as being associated with at least two health domains (among cardiometabolic, respiratory/allergy and mental health) in previous ExWAS studies conducted on the HELIX population [3–8]. It validates the assumption that using a  general health score allows to identify the exposures associated with multiple health parameters.

Noteworthy, our study identified three exposures, namely pets, the presence of a bluespace and physical activity, that were not identified in previous HELIX studies on single health outcomes. It confirms our hypothesis on the added value of this approach which is able to detect exposures associated in a low-grade manner with multiple health parameters. In particular, this study indicates that the presence of pets during childhood could improve the overall child's health. The literature on pet’s exposure reports conflicting findings on its impact on allergies and asthma [36, 37]. Pets is a well-established source of allergens [38, 39] but being exposed to them early in life could actually prevent allergic diseases [40–42] through microbial and immune mechanisms [43]. Additionally, the literature supports that the presence of pets is associated with lower blood pressure and heart rate [44] as well as lower anxiety [45]. Moreover, our findings add to the limited but growing literature on the beneficial health impact of the presence of a bluespace nearby [46]. To the best of our knowledge, very few studies focused on the pregnancy period, but past studies in adults showed an association between better perceived health with the density of “coastal” land [47] and the proximity of coast [48]. Finally, our study confirmed the benefits of physical activity on child’s BMI [49], respiratory [50] and mental health [51, 52].

Unexpectedly, some positive associations have been found between postnatal blood concentration to three persistent organic pollutants (PFOA, HCB and DDE) and the child’s general health score. These cross-sectional associations could be due to an inverse causality phenomenon, with lower blood levels of DDE and HCB in overweight children due to accumulation in fat. When stratifying on BMI, opposite trends of associations were found for HCB in the low *vs.* high BMI groups, which supports that the body composition might impact these associations. Plus, a non-linear association was suggested for HCB, calling for further investigations. A confounding bias due to fish consumption could be induced for PFASs [35] but further adjusting on total fish consumption did not change the results. Our results are in agreement with similar unexpected results previously found in the HELIX population [4, 5, 8].

### Strength and limitations of this study

This study has several strengths including first the longitudinal design of the HELIX project that allowed for an extended study of the exposome, with a wide range of exposures measured both during pregnancy and childhood using standardised protocols for each cohort site. A novelty of this study lies in the use of a general health score built by aggregating fifteen health parameters, covering three health domains with frequent childhood disorders: the cardiometabolic health (overweight), the respiratory health and allergies (asthma) and mental health (anxiety and behavioural disorders). A further strength relates in the ability of this approach to highlight exposures particularly harmful because affecting several health domains simultaneously, which can help prioritising public health messages.

However, we acknowledge that our study has some limitations. Some errors in exposure assessment could impact the statistical power, in particular regarding the least persistent pollutants like phenols and phthalates [53]. More generally, variability in measurement error between the exposures limits the ability to hierarchize the risk factors. Also, results regarding cross-sectional associations may suffer from reverse causality bias, for example the concentration of some persistent pollutants could be influenced by the child's health (through fat mass) instead of vice versa. In addition, the general health score, designed for etiological research but not for clinical purposes, has not been validated clinically. Finally, the same dataset has been used for the optimization of lambda and the model estimation which can be considered as a limit, even though cross-validation has been used for the first step.

### Public health impact

The identified early-life environmental exposures associated with the general health of children, are suspected to have an impact on several health parameters simultaneously, calling for prioritized public health messages. In terms of public health recommendations, it is helpful to disentangle environmental risk factors affecting multiple health outcomes to those affecting a single health outcome or affecting in different direction several health outcomes.

## Conclusion

This first exposome study on child’s general health attempted to approach the system nature of multiple exposures from our environment that shape multiple health outcomes. Our results reinforced the impact of several environmental risk factors (prenatal exposure to smoking, postnatal exposure to methylparaben, indoor air pollutants, caffeine and few social contacts) and protective factors (high intake of vegetables) on child’s health and identified new environmental protective factors (bluespace, pets) which calls for further investigation.

## Fundings

The study received funding from the European Community’s Seventh Framework Programme (FP7/2007–206) (grant agreement no 308333 - HELIX project) and the H2020-EU.3.1.2. - Preventing Disease Programme (grant agreement no 874583 - ATHLETE project). JJ holds a Miguel Servet-II contract (grant CPII19/00015) awarded by the Instituto de Salud Carlos III (cofunded by the European Social Fund “Investing in your future”). RM and JW are supported by the NIHR Applied Research Collaboration for Yorkshire and Humber (NIHR200166). The views expressed are those of the author(s) and not necessarily those of the NIHR or the Department of Health and Social Care. LM is funded by a Juan de la Cierva-Incorporación fellowship (IJC2018-035394-I) awarded by the Spanish Ministerio de Economía, Industria y Competitividad. NS has received funding from the Ministry of Science and Innovation and State Research Agency through the “Centro de Excelencia Severo Ochoa 2019-2023” Program (CEX2018-000806-S) and from IJC2020-043630-I financed by MCIN/AEI/10.13039/501100011033 and the European Union “NextGenerationEU/PRTR”. The Born in Bradford (BiB) cohort study was supported by infrastructure grant 101597 from the Wellcome Trust and joint grant MR/N024391/1 from the UK Medical Research Council and UK Economic and Social Science Research Council. Data collection at Infancia y Medio Ambiente (INMA) was supported by grants from the Instituto de Salud Carlos III, Centro de Investigacion Biomedica en Red Epidemiologia y Salud Publica, and the Generalitat de Catalunya-CIRIT. The Kaunas cohort (KANC) was supported by grant 6-04-2014_31V-66 and on September 13, 2015, by No. 31V-77, from the Lithuanian Agency for Science Innovation and Technology. A full list of support for the Etude des Determinants Pre et Postnatals du Developpement et de la Sante de l’Enfant (EDEN) cohort is found in Heude B et al. Cohort profile: the EDEN mother-child cohort on the prenatal and early postnatal determinants of child health and development. Int J Epidemiol. 2016;45(2):353-363. The Norwegian Mother and Child Cohort Study (MoBa) is supported by the Norwegian Ministry of Health and the Ministry of Education and Research, NIH/NIEHS (contract no N01-ES-75558), and NIH/NINDS (grant no.1 UO1 NS 047537-01 and grant no.2 UO1 NS 047537-06A1). The RHEA Mother Child Cohort was supported by European projects and the Greek Ministry of Health (Program of Prevention of Obesity and Neurodevelopmental Disorders in Preschool Children, Heraklion, Crete, Greece: 2011–2014; “Rhea Plus”: Primary Prevention Program of Environmental Risk Factors for Reproductive Health, and Child Health: 2012–2015).

## Supplementary Information

**Additional file 1:** **eMethods 1.** Health outcomes assessment. **eMethods 2.** Urban exposure assessment methods (extracted from Maitre^12^). **eMethods 3.** Assessment methods for lifestyle factors and other (extracted from Maitre^12^). **eMethods 4.** Biomarker assessment methods (extracted from Maitre^12^). **eMethods 5.** Creation of the general health score. **eMethods 6.** Data pre-processing. **eMethods 7.** Exposome-health association study. **eTable 1.** Collection time points of health parameters in childhood (mean, SD). **eTable 2.** Biological matrices of maternal and child samples. **eTable 3.** Collection time points of prenatal exposures, **eTable 4.**Collection time points of maternal blood and urine samples (mean, SD). **eTable 5.** Standardization of the cardiometabolic parameters. **eTable 6.** Standardization of the mental and cognitive parameters. **eTable 7.** Factor analysis on respiratory and allergy-related parameters – Dimension 1. **eTable 8.** Factor analysis on mental and cognitive parameters – Dimension 1. **eTable 9.** Transformation applied to continuous postnatal exposures and IQR. **eTable 10.** Description of transformed urban exposures. **eTable 11.** Description of exposures regarding the lifestyle. **eTable 12.** Description of biomarkers in the Helix population. **eTable 13.** Description of the health parameters considered. **eTable 14.** Correlations between the three sub-scores. **eTable 15.** Description of the general health score by covariates. **eTable 16.** Description of the general health score divided in terciles with health parameters and- sub-scores. **eTable 17.** Complete results of the ExWAS on prenatal exposures. **eTable 18.** Complete results of the ExWAS on postnatal exposures. **eTable 19.** Final multivariable models with and without fish consumption as a confounder (results for postnatal PFOA). **eFigure 1.** Variable plot - Mixed Factorial Analysis on standardized variables of mental health (Helix subcohort, n=1278 with mental health data). **eFigure 2.** Variable plots - Mixed Factorial Analysis on standardized variables of respiratory health and allergies (Helix subcohort, n=1009 with respiratory and allergic data). **eFigure 3.** Distribution of each sub-score by cohort. **eFigure 4.** Multi-exposure model on postnatal exposures stratifiedon the terciles of z-BMI. **eFigure 5.** Multi-exposure model on prenatal exposures stratified on sex. **eFigure 6.** Multi-exposure model on postnatal exposures stratified on sex. **eFigure 7.** Multi-exposure model on postnatal exposures stratified on cohorts. **eFigure 8.** Multi-exposure model on postnatal exposures stratified on cohorts. **eFigure 9.** GAM estimated curve for postnatal selected exposures by LASSO. **eFigure 10.** Multi-exposure model without the 4% most extreme values for the general health score.

## Data Availability

The datasets analysed during the current study are not available because they contain multiple sensitive identifiers.

## Abbreviations

ADHD: Attention Deficit Hyperactivity Disorders
BMI: Body Mass Index
BiB: Born in Bradford
BPA: Bisphenol-A
BUPA: Butyl-paraben
Cd: Cadmium
Co: Cobalt
Cu: Copper
DDE: Dichlorodiphenyldichloroethylene
DEHP: Di EthylhexylPhthalate
DEP: Diethyl phosphate
DMP: Dimethyl phosphate
DMTP: Dimethyl thiophosphate
EDEN: Étude des Déterminants pré et postnatals du développement et de la santé de l’Enfant
ETPA: Ethylparaben
FWER: Family Wise Error Rate
HCB: Hexachlorobenzene
Hg: Mercury
INMA: Infancia y Medio Ambiente
IOTF: International Obesity Task Force
KANC: Kaunus Cohort
KIDMED: Mediterranean diet in children
MBzP: Mono benzyl phthalate
DiNP: Diisononyl phthalate
MEP: Monoethyl phthalate
MEPA: Methylparaben
Mo: Molybdenum
MiBP: Mono-iso butyl phthalate
MoBa: The Norwegian Mother Father and Child Cohort Study
NDVI: Normalized difference vegetation index
NO2: Nitrogen dioxide
OC: Organochlorine compound
OP: Organophosphate pesticide
OXBE: Oxybenzone
Pb: Plomb
PBDE: Polybrominated diphenyl ether
PCB: Polychlorobiphenyls
PFASs: Per- and polyfluoroalkyl substance
PFHxS: Perfluorohexane sulfonate
PFNA: Perfluorononanoate
PFOA: Perfluorooctanoate
PFOS: Perfluorooctane sulfonate
PFUNDA: Perfluoroundecanoate
PM: Particulate matter
PRPA: Propyl-paraben
RHEA: Mother–Child Cohort in Crete
Se: Selenium
Tl: Thalium
TRCS: Triclosan
UV-Vit. D: Vitamin-D dose from ultraviolet
