# Supplementary material for: Environmental exposures in early-life and general health in childhood
Source: Environ Health. 2023 Jul 21;22:53. doi: 10.1186/s12940-023-01001-x (PMC10360263; doi:10.1186/s12940-023-01001-x)
Supplement: Supplementary file 1 — Additional file 1: eMethods 1. Health outcomes assessment. eMethods 2. Urban exposure assessment methods (extracted from Maitre12). eMethods 3. Assessment methods for lifestyle factors and other (extracted from Maitre12). eMethods 4. Biomarker assessment methods (extracted from Maitre12). eMethods 5. Creation of the general health score. eMethods 6. Data pre-processing. eMethods 7. Exposome-health association study. eTable 1. Collection time points of health parameters in childhood (mean, SD). eTable 2. Biological matrices of maternal and child samples. eTable 3. Collection time points of prenatal exposures, eTable 4.Collection time points of maternal blood and urine samples (mean, SD). eTable 5. Standardization of the cardiometabolic parameters. eTable 6. Standardization of the mental and cognitive parameters. eTable 7. Factor analysis on respiratory and allergy-related parameters – Dimension 1. eTable 8. Factor analysis on mental and cognitive parameters – Dimension 1. eTable 9. Transformation applied to continuous postnatal exposures and IQR. eTable 10. Description of transformed urban exposures. eTable 11. Description of exposures regarding the lifestyle. eTable 12. Description of biomarkers in the Helix population. eTable 13. Description of the health parameters considered. eTable 14. Correlations between the three sub-scores. eTable 15. Description of the general health score by covariates. eTable 16. Description of the general health score divided in terciles with health parameters and- sub-scores. eTable 17. Complete results of the ExWAS on prenatal exposures. eTable 18. Complete results of the ExWAS on postnatal exposures. eTable 19. Final multivariable models with and without fish consumption as a confounder (results for postnatal PFOA). eFigure 1. Variable plot - Mixed Factorial Analysis on standardized variables of mental health (Helix subcohort, n=1278 with mental health data). eFigure 2. Variable plots - Mixed Factorial Analysis on standardized variabl [file 12940_2023_1001_MOESM1_ESM.docx]

**Supplementary Online Content**

Amine I, Guillien A, Philippat C, et al. Multiple environmental exposures in early-life and general health in childhood.

Table of contents

[eMethods 1. Health outcomes assessment 3](#_Toc132271576)

[eMethods 2. Urban exposure assessment methods (extracted from Maitre^12^) 5](#_Toc132271577)

[eMethods 3. Assessment methods for lifestyle factors and other (extracted from Maitre^12^) 9](#_Toc132271578)

[eMethods 4. Biomarker assessment methods (extracted from Maitre^12^) 11](#_Toc132271579)

[eMethods 5. Creation of the general health score 14](#_Toc132271580)

[eMethods 6. Data pre-processing 15](#_Toc132271581)

[eMethods 7. Exposome-health association study 16](#_Toc132271582)

[eTable 1. Collection time points of health parameters in childhood (mean, SD) 17](#_Toc132271583)

[eTable 2. Biological matrices of maternal and child samples 18](#_Toc132271584)

[eTable 3. Collection time points of prenatal exposures 19](#_Toc132271585)

[eTable 4. Collection time points of maternal blood and urine samples (mean, SD) 20](#_Toc132271586)

[eTable 5. Standardization of the cardiometabolic parameters 21](#_Toc132271587)

[eTable 6. Standardization of the mental and cognitive parameters 22](#_Toc132271588)

[eTable 7. Factor analysis on respiratory and allergy-related parameters – Dimension 1 23](#_Toc132271589)

[eTable 8. Factor analysis on mental and cognitive parameters – Dimension 1 24](#_Toc132271590)

[eTable 9. Transformation applied to continuous postnatal exposures and IQR 25](#_Toc132271591)

[eTable 10. Description of transformed urban exposures 27](#_Toc132271592)

[eTable 11. Description of exposures regarding the lifestyle 30](#_Toc132271593)

[eTable 12. Description of biomarkers in the Helix population 33](#_Toc132271594)

[eTable 13. Description of the health parameters considered 36](#_Toc132271595)

[eTable 14. Correlations between the three sub-scores 37](#_Toc132271596)

[eTable 15. Description of the general health score by covariates 38](#_Toc132271597)

[eTable 16. Description of the general health score divided in terciles with health parameters and sub-scores 39](#_Toc132271598)

[eTable 17. Complete results of the ExWAS on prenatal exposures 40](#_Toc132271599)

[eTable 18. Complete results of the ExWAS on postnatal exposures 42](#_Toc132271600)

[eTable 19. Final multivariable models with and without fish consumption as a confounder (results for postnatal PFOA). 45](#_Toc132271601)

[eFigure 1. Variable plot - Mixed Factorial Analysis on standardized variables of mental health (Helix subcohort, n=1278 with mental health data) 46](#_Toc132271602)

[eFigure 2. Variable plots - Mixed Factorial Analysis on standardized variables of respiratory health and allergies (Helix subcohort, n=1009 with respiratory and allergic data) 47](#_Toc132271603)

[eFigure 3. Distribution of each sub-score by cohort 48](#_Toc132271604)

[eFigure 4. Multi-exposure model on postnatal exposures stratified on the terciles of z-BMI. 49](#_Toc132271605)

[eFigure 5. Multi-exposure model on prenatal exposures stratified on sex 50](#_Toc132271606)

[eFigure 6. Multi-exposure model on postnatal exposures stratified on sex 51](#_Toc132271607)

[eFigure 7. Multi-exposure model on postnatal exposures stratified on cohorts 52](#_Toc132271608)

[eFigure 8. Multi-exposure model on postnatal exposures stratified on cohorts 53](#_Toc132271609)

[eFigure 9. GAM estimated curve for postnatal selected exposures by LASSO 54](#_Toc132271610)

[eFigure 10. Multi-exposure model without the 4% most extreme values for the general health score 55](#_Toc132271611)

[eReferences 56](#_Toc132271612)

#

# eMethods 1. Health outcomes assessment

*Cardiometabolic outcomes* ^1,2^

Blood pressure was measured during the clinical examination using a standardized protocol: after 5 minutes of rest in sitting position, 3 consecutive measurements were taken by oscillometric device (OMRON 705-CPII) with one-minute time intervals between them, in a predefined posture and in preference in the right arm. Adequate cuff sizes were chosen with respect to each child’s arm length and circumference. Systolic (SBP) and diastolic (DBP) blood pressures from each measurement have been recorded and the mean of the second and the third measurements was calculated and used in this analysis.

During the subcohort examination, the waist circumference was measured as an indicator of visceral fat in duplicate to the nearest 0:1 cm in a standing position, at the high point of the iliac crest at the end of a gentle expiration, with the use of a measuring tape (Seca 201; Seca Corporation). Also, height and weight were measured using regularly calibrated instruments. In complementary analysis, we used BMI age-and-sex–standardized z-scores (zBMI) using the international World Health Organization (WHO) reference curves ^3^. For all measures, we used common standardized protocols and the same instruments across the cohorts.

Concentrations (mg/dL) of triglycerides and high-density lipoprotein (HDL) cholesterol was measured in child nonfasting serum using homogeneous enzymatic colorimetric methods on a Modular Analytics System from Roche Diagnostics GmbH Mannheim and according to the manufacturer’s instructions ^4^. Insulin levels were measured in serum using human adipokine 15-plex magnetic panels ^5^.

*Respiratory and allergic outcomes* ^6,7^

Lung function was measured by a spirometry test (EasyOne spirometer; NDD [New Diagnostic Design], Zurich, Switzerland), by trained research technicians using a standardised protocol. The child, sitting straight and equipped with a nose clip, was asked to perform at least six manoeuvres (if possible). Data from unacceptable manoeuvres as a result of errors, including hesitation or false starts, cough, variable efforts, glottis closure, early termination, and leaks, were not retained by the technicians. The protocol required that at least three acceptable manoeuvres were obtained, and that they were reproducible, defined as a difference of less than 200 mL between the two highest values for forced vital capacity (FVC) and Forced Expiratory Volume in one second (FEV₁) taken from the acceptable manoeuvres. We then applied the following validation criteria on the spirometer curves retained by the technicians. We defined a manoeuvre as acceptable if there was no hesitation or false start (defined as a ratio of backward extrapolated volume [BEV] to FVC of <5% or a BEV of <100 mL if FVC was <1000 mL) and if the forced expiratory time was in an acceptable range (>1·5 s and <10 s). The two highest values for FEV₁ taken from acceptable forced expiratory manoeuvres could not vary by more than 150 mL or by more than 5% from the second FEV₁. To address the efficiency of the FEV₁ data cleaning, the 243 examinations from the INMA cohort (185 of which were included in the HELIX study) were further investigated by trained investigators who looked at the shape of the curves; for 192 (79%) of the 243 examinations, the same curve was selected, and for the remaining 51 (21%), the Pearson correlation between the FEV₁ of the two different curves was 0·96. We used the reference equations estimated by the Global Lung Initiative22 for computing the FEV₁ percent predicted values (ie, values standardised by age, height, sex, and ethnicity of the patient) and FEV₁ Z scores. After excluding extreme values (ie, FEV₁ <60% or >140%, which were probably due to measurement error in our young population), we selected the greatest FEV₁ value at an individual level among all accepted curves, hereafter referred to as FEV₁%. For this analysis, we used FEV₁%. as our main measure of the lung function.

Information on the asthma and allergy-related outcomes were obtained through questions adapted from the International Study on Asthma and Allergy in Childhood (ISAAC) ^8^ during the interview with the mother at the follow-up examination. “Has your child ever been diagnosed by a doctor as having asthma?” (hereafter called asthma); “Has your child ever been diagnosed by a doctor as having eczema or atopic dermatitis or neurodermatitis?” (hereafter called eczema); “Has your child ever had an allergic reaction to food, diagnosed by a doctor?” (hereafter called food allergy); “In the last year, has your child had problems with sneezing, or a runny, or blocked nose when he/she did not have a cold or the flu?” (hereafter called rhinitis).

*Mental outcomes*

Trained fieldwork technicians measured the fluid intelligence using a computer-based test which is the Raven Colour Progressive Matrix™ ^9^ (CPM). The CPM comprised a total of 36 items and we used the total number of correct responses as the outcome. A higher CPM scoring indicates better fluid intelligence. Fluid intelligence is the ability to solve novel reasoning problems and depends only minimally on prior learning. All examiners were previously trained following a standardized assessment protocol by the study expert psychologist. Furthermore, during the pilot phase, a coordinator visited each cohort site and checked for any potential error committed by the previously trained examiners.

Parents completed questionnaires related to child’s behaviour, including the child behaviour checklist (CBCL) and the Conner rating scale’s, within a week before the follow-up visit at 6–12 years of age. The 99-item CBCL/6–18 version for school children was used to obtain standardized parent reports of children’s problem behaviours, translated and validated in each native language of the participating six cohort populations ^10^. The parents responded along a 3-point scale with the code of 0 if the item is not true of the child, 1 for sometimes true, and 2 for often true. The internalizing score includes the subscales of emotionally reactive and anxious/depressed symptoms, as well as somatic complaints and symptoms of being withdrawn. The externalizing score includes attention problems and aggressive behaviours. In addition, an ADHD index based on the short form of the Conners’ rating scales of 27 items provided information on inattention and hyperactivity symptoms ^11^. The internal consistency (Cronbach’s alpha) of each of the study scales was >0.80. All outcomes were analyzed as raw count scores.

# eMethods 2. Urban exposure assessment methods (extracted from Maitre^12^)

Outdoor and urban exposures were assessed in the following exposure groups: Atmospheric pollutants, ultraviolet (UV) radiation, surrounding natural space, meteorological measures, built environment, traffic, road traffic noise, water disinfection by-products and indoor air pollutants. Except for the last two groups, exposure assessment was conducted within the PostgreSQL (copyright © 1996-2017 The PostgreSQL Global Development Group), PostGIS (Creative Commons Attribution-Share Alike 3.0 License http://postgis.net) and QGIS (QGIS Development Team, 2016. QGIS Geographic Information System) platforms. For the pregnancy period, exposure was assessed at the geocoded residential address of each woman. For each woman, assessment of exposure during pregnancy at the geocoded residential address at recruitment was made. For the childhood period, exposure was assessed at the geocoded residential and school addresses of each child as reported at the time of the subcohort visit. In case of multiple addresses, results were averaged by mother or child.

*Exposures selected*

- Noise levels were removed because missing in at least 2 entire cohorts and/or for more than 30% of the total Helix population before imputation.
- For sets of variables that measure a very similar exposure and that exhibit correlations higher than 0.9, only one exposure representative of the group was included in the list of exposome variables. For example, this was the case for different UV variables or variables in the built environment calculated for different spatial buffers.
- Home-school correlations were checked and for some exposures (air pollution, humidity, temperature, UV), if the correlation was over 0.9 then school exposures were removed.
- Some exposure variables were calculated for different time windows as described below (e.g. atmospheric pollutants average for the day, week, and year before the subcohort follow-up visit); for this paper we always used the longest available time period.

*Atmospheric pollutants*

The following atmospheric pollutants were assessed: nitrogen dioxide (NO_2_), particulate matter with an aerodynamic diameter of less than 2.5 μm (PM_2.5_) and of less than 10 µm (PM_10_), and absorbance of PM_2.5_ filters (PM_abs_). These were assessed using land use regression (LUR) or dispersion models, temporally adjusted to measurements made in local background monitoring stations and averaged over the periods of interest. In most cases we used site-specific LUR models developed in the context of the European Study of Cohorts for Air Pollution Effects (ESCAPE) project ^13–15,16(p201),17^. For BiB, assessment for PM_2.5_ and PM_10_ was made based on the ESCAPE LUR model developed in London/Oxford (UK) and adjusted for background PM levels from monitoring stations in Bradford ^18^. For EDEN, the ESCAPE European-wide LUR model was applied for PM_2.5_ ^19^, and dispersion models were used to assess NO_2_ and PM_10_ exposure (the latter only for the pregnancy period) ^20^. Data on daily background concentrations of air pollutants for temporal adjustment were obtained from routine background stations active during the whole study period. Back-extrapolation based on other available pollutants was used when data on a pollutant were not available. In particular, daily PM_10_ was used to adjust NO_2_; daily NO_2_ or PM_10_ factors to adjust PM_2.5_; daily NO_2_ to adjust PM_10_; and daily NO_x_ to adjust PM_abs_. For the pregnancy period the exposure estimates were calculated for the three pregnancy trimesters and as the mean of whole pregnancy period. For this manuscript, the pregnancy period was selected as the main exposure variable. For childhood period, exposure calculated as the average over one day, one week and one year before the date of the subcohort follow-up examination of the child; this was done for the home address and school address. The day and the year before examination at home address were selected as the main exposures for this manuscript.

*Surrounding natural space*

We followed the PHENOTYPE protocol^21^ to measure the surrounding greenness, i.e. trees, shrubs and parkland, and applied the Normalized Difference Vegetation Index (NDVI)^22^ derived from the Landsat 4–5 Thematic Mapper (TM), Landsat 7 Enhanced Thematic Mapper Plus (ETM+), and Landsat 8 Operational Land Imager (OLI)/Thermal Infrared Sensor (TIRS) with 30m × 30m resolution (courtesy of the  U.S. Geology Survey). NDVI quantifies greenness by measuring the difference between near-infrared (which vegetation strongly reflects) and red light (which vegetation absorbs). NDVI values range from +1.0 to -1.0, with higher numbers indicating more greenness. To achieve maximum exposure contrast, we used available cloud-free Landsat images during the period between May and August for years relevant to our period of study and calculated greenness within 100, 300 and 500 meter buffers around each address. Furthermore, an indicator for “residential proximity to major green spaces” was created, as it covers different aspects of natural space exposure, i.e. easy access to recreational space. We calculated access to major green spaces (parks or countryside) and major blue spaces (bodies of water) as the straight-line distance from the home or school to nearest blue or green space with an area greater than 5000 m^2^ from topographical maps ^23,24^ or local sources. We also created a dichotomous variable to define whether a major green or blue space was present or not within a buffer of 300 m. For the pregnancy period the presence of a major blue or green space, and NDVI within a 100 meter buffer, were selected as the main exposure variables. For the childhood period, we selected the presence of a major blue or green space and NDVI in a 100 meter buffer (at home and at school).

*Meteorological variables*

We used meteorological stations in the study area to obtain data on temporal variability in temperature. Daily measurements of temperature and humidity were obtained from a local weather station in each study area and averaged over each period of interest. Atmospheric pressure data were obtained from the ESCAPE project, and were available only for pregnancy trimesters and the entire pregnancy period (pregnancy mean was selected as main exposure), not for the childhood period. During the childhood period temperature and humidity were estimated for the home and school address. Daily, weekly and monthly measurements of UV radiation (as erythemal UV, Viamin-D and DNA damaging UV) at home and at school at 0.5 x 0.5 degree resolution were obtained from the Global Ozone Monitoring Experiment onboard the ERS-2 (European Remote Sensing) satellite (Temis), and averaged over the day, week and month before the subcohort follow-up examination. For the childhood period, monthly UV-vit D at home address was selected as main exposure variable. For the pregnancy period, estimates were not available.

*Built environment*

Topological maps for the following built environment indicators were obtained from local authorities or from Europe wide sources. Building density was calculated within 100 and 300 meters buffer by dividing the area of building cover (m^2^) by the area of each buffer (km^2^). Population density was calculated as the number of inhabitants per km^2^ surrounding the home address. Street connectivity was calculated as the number of street intersections inside 100 and 300 meters buffer, divided by the area (km^2^) of each buffer. Facility richness index was calculated as the number of different facility types present divided by the maximum potential number of facility types specified, in a buffer of 300 meters, giving a score of 0 to 1. Facility density index was calculated as the number of facilities present divided by the area of the 300 meters buffer (number of facilities/km^2^). A higher value indicates a more availability of different facility types. Landuse Shannon's Evenness Index (SEI) was calculated to provide the proportional abundance of each type of land use in a buffer of 300 meters, giving a score between 0 and 1 (Shannon and E. 2001). It was calculated by multiplying each proportion of land use type by its logarithm and dividing the sum of all land use type products by the logarithm of the total possible land use types. We developed an indicator of walkability, adapted from the previous walkability indexes ^25–27^, calculated as the mean and sum of the deciles of population density, street connectivity, facility richness index and land use SEI within 300 meters buffers, giving a walkability score ranging from 0 to 1. Accessibility was measured by BST (bus public transport) lines and stops were obtained from local authorities of each study area and from Open Street Maps ^28^ where local layers were not available. BST lines density was calculated as meters of BST lines inside 100, 300 and 500 meters buffer, divided by the buffer area in square kilometers. BST stop density was calculated as number of BST inside 100, 300 and 500 meters buffer, divided by the buffer area in square kilometers. For the current manuscript, we selected the 300 meter buffer at home and school address as main exposure during pregnancy and childhood periods.

*Road traffic*

Traffic density indicators (traffic density on nearest road, traffic load on all and major roads within 100 m buffer and inverse distance to nearest road) were calculated from road network maps following the ESCAPE protocol ^16,17^. A fieldwork campaign was conducted in Heraklion during 2015, to assess multiple exposures as previously described ^29^. Briefly, measurements of manual traffic counts of light and heavy vehicles over 15 minutes, and of noise, averaged over 30 minutes monitoring (Sonometer SC160, CESVA monitors -Spain), were made in 160 sites around the city. Sites were chosen representing multiple types (e.g. traffic, urban background, urban green etc.). During the campaign each monitoring site was measured three times in different seasons (summer, winter and autumn). We applied the LUR methods and GIS predictor variables used within the ESCAPE project and described in Eftens ^17^ develop LUR models of traffic count and road traffic noise. For the analyses in this manuscript, we selected, the total traffic load on major roads in a 100 m buffer (home and school), the total traffic load on all roads in a 100 m buffer (pregnancy and home), traffic density on nearest road (pregnancy and home), and inverse distance to nearest road (pregnancy and home).

*Road traffic noise*

Noise levels, i.e. Lden (annual average sound pressure level of 24h period: day, evening and) and Ln (annual average sound pressure level of night period) were derived from noise maps produced in each local municipality under the European Noise Directive (EC Directive 2002/49/EC (EUR-Lex, n.d.)). To improve comparability between centers, the values were categorized into six categories (<55; 55-59.9; 60-64.9; 65-69.9; 70-74.9; >75) for analysis. For RHEA, estimates on noise were newly modeled following new fieldwork. For this manuscript, noise levels were not used as missing data was above 30% before imputation.

*Water Disinfection By-Products (DBPs)*

We collected data on routine measurements of disinfection by-product (DBP) in water from water companies for the all cohorts for the pregnancy period. For KANC, BiB, INMA and RHEA cohorts this was built on the HiWate project (Health Impacts of long-term exposure to disinfection by-products in drinking Water) ^30^ that previously modeled exposure levels in the water supply of the residence of each participating mother-child pair. For BiB, routine monitoring data on trihalomethanes (THMs) were obtained for the eight water supply zones covering the study area. Each zone was sampled nine times per year on average, giving 374 data points in total ^31^. For INMA, levels of THMs were ascertained based on sampling campaigns and regulatory data from local authorities and water companies. Sampling locations were defined to be geographically representative of the study areas, and water samples were collected from taps with no filtration or other treatments that could affect THMs concentration. THMs were determined in 198 places ^32^. For RHEA, the city was divided into six zones according to the source of underground water used in each area, corresponding to six different water treatment plants. In total, 18 sampling points were selected (12 areas in Heraklion and 6 in rural areas), which covered geographically the residences of participating mother-child pairs ^33^. For KANC, tap water THM concentration, derived as the average of quarterly sample values over the time that the pregnancy occurred from all sampling sites located in each distribution system, and geocoded maternal address at birth to assign the individual women’s residential exposure index ^34^. Routine DBP measurements were acquired for MoBa and EDEN cohorts as these cohorts were not part of the HiWate project. THMs exposure levels were modeled for each residence, following the protocol developed within HiWate ^30^. We estimated exposure to total THMs, and for chloroform and brominated THMs separately during each pregnancy and in the entire pregnancy; for the current manuscript, the pregnancy averages were used.

*Indoor Air Pollutants*

Indoor air concentrations of nitrogen dioxide (NO_2_), particulate matter <2.5μm (PM_2.5_), particulate matter absorbance (PM_abs_), benzene, and toluene, ethylbenzene, xylene (TEX) were estimated through a prediction model that combined measurements in the homes of a subgroup of children with questionnaire data from the subcohort.

Measurements of indoor NO_2_, benzene and TEX were conducted in the homes of 157 participants as part of the child panel study, which was nested within the HELIX subcohort in all cohorts except MoBa. PM_2.5_ and PM_Abs_ were measured in INMA, BiB, and EDEN. Participants in the child panel study were followed for one week in two seasons, and the last day of the first week coincided with the subcohort examination, including the completion the main HELIX questionnaire. NO2, benzene and TEX sampling lasted 7 days, and PM_2.5_ and PM_Abs_ sampling lasted 24 hours.

NO_2_ short-term diffusive Passam samplers were used to measure indoor NO_2_ concentrations. The samplers were composed of polypropylene housing with a 20 mm diameter opening, covered with a removable plastic cap and protected from wind disturbance by a teflon membrane. Triethanolamine was used as absorbent material inside the tube. NO_2_ was collected by molecular diffusion to the absorbent and its concentration was determined spectrophotometrically by the Saltzmann method. The detection limit (DL) for a week’s sampling for the NO_2_ sampler was 0.3 µg/m^3^. Passam ORSA5 diffusion tubes were used to measure indoor levels of benzene, toluene, ethylbenzene and ortho-, para- and metaxylenes. The DL for a week’s sampling for each compound was 0.4 µg/m^3^. The samplers were placed in the living rooms of the participating homes, away from the sources of ventilation. After collection, the NO_2_, Benzene and TEX samplers were hermetically sealed and kept in zip-lock bags in boxes, in a cool and dark place and shipped to the analyzing laboratory within 3 months of the end of the sampling campaign.

For indoor PM modeling, active PM_2.5_ cyclone pumps were placed in the living room. After 24 hours the samplers were collected and sent to laboratory. PM_2.5_ mass was collected gravimetrically using 37-mm Teflon filters held in a cyclone (model GK2.05 SH, BGI Inc., Waltham MA, USA) with an aerodynamic cut point of 2.5 µm and connected to a BGI/Mesa Labs A4004 pump working at 3.5L/min. Filter weighing and reflectance measurements were conducted with a microbalance of 1 µg accuracy (Model MX5, Mettler-Toledo International Inc., Switzerland) and a Smoke Stain Reflectometer (SSR) (Model 43D, Diffusion Systems Ltd., UK), respectively. Measurement procedures, quality control, as well as PM_2.5_ mass concentration and absorbance estimations followed the ESCAPE project protocols (both available at [www.escapeproject.eu/manuals](http://www.escapeproject.eu/manuals)).

Statistical analyses were performed separately for each of the exposure variables. A TEX variable was created by summing the concentrations of each TEX compound. The HELIX main questionnaire ^35^ was used to identify housing and participant characteristics as input for the prediction model; these characteristics included: exposure to environmental tobacco smoke, cooking and heating methods at the home, cleaning products between others.

After extracting potential predictor variables from the questionnaires, bivariate analyses were run by either Kruskal-Wallis or Wilcoxon rank sum tests, as all of the potential predictors were categorical and the exposure variables were not normally distributed. The variables that yielded a p value lower than 0.2 in bivariate analyses were selected to enter into the multiple linear regression models. Prior to that, univariate linear regressions were performed for each of the predictors selected in the bivariate analysis in order to assess the adjusted determination coefficient (adjusted R^2^) for each of them individually. To ensure normality of the distributions of the outcome variables, the univariate linear regression models and subsequent multiple linear regression models were built using log-transformed.

Supervised forward stepwise procedure was employed to build multiple linear regression models. In all cases the starting point for the regression was the variable which yielded the highest adjusted R^2^ in the univariate linear regressions. Then the other predictors were added one-by-one and additional increase in the adjusted R^2^ was recorded. The variable which increased the adjusted R^2^ by a highest value was retained in the model and the procedure was repeated until none of the variables increased the adjusted R^2^ by at least 1%. In case any of the variables included into the model had individual p value equal or higher than 0.05, it was removed from the model. All statistical analyses were performed using R Statistic Software (version 3.4.1).

The best explained pollutant was NO_2_ with an R^2^ of 57%, followed by PM_Abs_ with 50%. For NO_2_, cohort, natural gas oven, type of hob and boiler, butane in the living room, and the number of people living in the house, were the statistically significant variables in the model; all of these were positively correlated. The prediction models were then use to estimate these 5 indoor air pollutants in the entire subcohort.

# eMethods 3. Assessment methods for lifestyle factors and other (extracted from Maitre^12^)

*Tobacco smoke*

Tobacco smoke exposure was assessed in pregnancy via questionnaire for active and passive smoking, as well as based on cotinine measurements (see eMethods 4). Pregnancy questions on tobacco smoke from the cohorts were harmonized as part of the ESCAPE project. Tobacco smoke exposure of the mother at any point during pregnancy was categorised into: no exposure, only passive smoke exposure, active smoking. Active smoking was also measured by the number of cigarettes per day on average during pregnancy

For children, in addition to the cotinine-based classification (see eMethods 4), the following two variables were created based on the questionnaires completed by the parents:

- The global exposure of the child to ETS with two categories: "no exposure", no exposure at home neither in other places; “exposure”: exposure in at least one place, at home or outside.
- Active smoking of the parents: “1” none of the parents, “2” one parent or “3” both parents.

*Diet*

Diet during pregnancy was assessed through food frequency questionnaires by each cohort and harmonized *a posteriori* for the HELIX project. Harmonisation was possible for eight main food groups (average consumption in times/week) and folic acid supplementation intake (yes/no) in the first trimester for five of the six cohorts (KANC not available).

In early childhood years information about breastfeeding duration (in weeks) was collected by the cohorts and then harmonized as part of HELIX.

Information on the child’s diet was collected through the standardized HELIX subcohort questionnaire. The child’s diet was then summarized in 15 food groups (times/week). Food groups collected during both pregnancy and childhood were cereals, dairy products, fish and seafood, fruits, meat and vegetables. During pregnancy only, legumes intakes was also measured. During childhood, 10 other food groups were assessed: bakery products, breakfast cereals, bread (white and whole wheat), potatoes, sweets, yogurt and probiotics, processed meat, total added lipids (butter, margarine and vegetable oils), beverages (sodas, with or without sugar), caffeinated drinks. We also assessed dietary habits, in particular, visits to a fast-food restaurant/take away (pregnancy and childhood), eating organic food (childhood only) and eating readymade supermarket meals (childhood only). We also included the KIDMED index, a dietary score representative of healthy eating and based on the principles of Mediterranean dietary patterns. The KIDMED index consists of 16 questions with questions denoting a negative connotation with respect to the Mediterranean diet assigned a value of -1, and those with a positive aspect scored +1 ^36^. Further, we analysed as separate variables few factors that contribute to the KIDMED index including fast food visits, organic food and ready-made supermarket meal consumption.

*Physical activity*

Physical activity during pregnancy (3^rd^ trimester only) was estimated based on the harmonization of the respective cohort questionnaire data. Two variables were created: (yes) moderate activity corresponding to walking and/or cycling activity (expressed in frequency categories: never or sometimes; often; very often); and (2) vigorous activity (in two frequency categories: low and medium/high) corresponding to exercise or sport activity. Because there were more than 30% of missing data before imputation, physical activity has not been studied for the pregnancy period for this manuscript.

For children, the moderate-to-vigorous physical activity variable was created based on questionnaire data. It was defined as the amount of time children spent doing physical activities with intensity above 3 metabolic equivalent tasks (METs), and is expressed in units of min/day. Physical activity over-reporting was corrected based on the accelerometer (Actigraph) correlation with questionnaire answers, using the data from three cities involved in the HELIX panels (nested study of the HELIX project where participants wore accelerometers for two non-consecutive weeks).

A variable representing sedentary behaviour in the children was created based on the questionnaire and corresponds to the duration of time spent watching TV, playing computer games or other sedentary games. This variable is a new concept which is commonly defined as ‘‘any waking behavior characterized by an energy expenditure <1.5 metabolic equivalent tasks (METs) while in a sitting or reclining posture’’ by the Sedentary Behaviour Research Network (Sedentary Behaviour Research Networ 2012). Sedentary behavior has been shown to be a health risk factor independently from physical activity.

*Alcohol*

Alcohol consumption during pregnancy was harmonized based on questionnaire data from the cohorts and classified as whether or not any alcohol was consumed during pregnancy (except in the KANC cohort where the lowest exposure category included women with less than 1 glass a month).

For this analysis, alcohol was not studied as it showed unexpected high prevalence among some cohorts (ex: 47% in MoBa while the national prevalence is 23%). Also, harmonization could not be fully completed with the KANC cohort, as explained just above.

*Allergens*

For allergen exposure only pet ownership (dog, cats or other) of the child was added to the exposome. There was no prenatal information on this.

*Sleep*

Sleep duration was available for the subcohort children, not for the mothers, and corresponds to the average sleep duration at night during an entire week (weighted average of weekdays and weekend sleep duration). This variable was calculated based on the questionnaire taking the average bedtime and wake-up time (earliest and latest bedtime/wake-up times available) during weekdays and weekends.

*Socio-economic capital*

Questions related to socio-economic position (maternal education and others) were collected during the pregnancy in all cohorts and harmonized for use as covariates in analyses; they were not included in the exposome as separate exposure variables. In the childhood exposome, the Family Affluence Score (FAS) was included based on questions from the subcohort questionnaire ^37^. A composite FAS score was calculated based on the responses to the next four items: (yes) Does your family own a car, van or truck? (2) Do you have your own bedroom for yourself? (3) During the past 12 months, how many times did you travel away on holiday with your family? (4) How many computers does your family own? ^37^. A three point ordinal scale was used, where FAS low (score 0,1,2) indicates low affluence, FAS medium (score 3,4,5) indicates middle affluence, and FAS high (score 6,7,8,9) indicates high affluence FAS ^38^. The FAS score in this study had only a maximum value of 7 instead of 9 because of the smaller number of possible answers for certain items.

Further social capital-related questions were included in the HELIX questionnaire to capture different aspects of social capital, relating both to the cognitive (feelings about relationships) and structural (number of friends, number of organizations) dimensions and to bonding capital (close friends and family), bridging capital (neighborhood connections, looser ties) and linking capital (ties across power levels; for example, political membership). Two summary variables were selected for the exposome analysis: social participation (membership of organizations: 0, 1, or 2) and contact with friends and family (daily, once a week, less than once a week). In addition, house crowding was included, representing the number of persons living in the house with the child.

# eMethods 4. Biomarker assessment methods (extracted from Maitre^12^)

For all the 1,301 children in the subcohort, biomarker the determinations of a set of chemical contaminants (organochlorine compounds, brominated compounds, perfluorinated alkylated substances (PFAS), metals and elements, phthalate metabolites, phenols, and organophosphate (OP) pesticide metabolites) were performed at the Department of Environmental Exposure and Epidemiology at the Norwegian Institute of Public Health (NIPH), in Norway or in collaboration with their contract laboratories. This was also the case for the majority of the maternal samples collected during pregnancy or at birth and stored in cohort biobanks; however, for some maternal samples in some cohorts, measurements were already completed at thus we used these results. Here we provide a summary of the methods used to determine biomarker levels for the chemical contaminants; more detailed information can be found in Haug et al ^39^.

*Exposures selected*

Some exposures have been removed/modified for the analysis:

- Prenatal exposures to PBDE47, PBDE153, ETPA, MEPA, DDT, PCB118, PCB170 and all essential minerals and metals except Hg were removed because missing in at least 2 entire cohorts and/or for more than 30% of the total Helix population before imputation.
- Some biomarkers had more than 30% values under the LOD and could not be re-coded: prenatal exposures to DDT, DETP, DMDTP and postnatal exposures to PBDE153, PRPA, PFUNDA, DETP, DMP and DMDTP. They were all removed from the analysis.
- High correlations (>0.9) were observed between individual PCBs and individual metabolites of DEHP (Phthalates). Sum variables were also created to combine the individual compounds into one variable. For the current analysis, combined variables were used instead of individual exposures.

*Quality assurance*

The sample collections for the children were performed in a completely harmonized way, using the same protocols and equipment for sample collection and processing in all the six cohorts ^35^. The children’s samples were randomized into batches before chemical analyses, aiming at a minimum of three cohorts to be included in each batch. However, this was not feasible for the maternal samples as the cohorts shipped the maternal samples at different time points to the laboratories for analysis.

*Organochlorine compounds (OCs)*

Concentrations of OCs were determined in serum or plasma according to Caspersen et al ^40^ except that gas chromatography–mass spectrometry (GC-MS/MS) was used instead of gas chromatography/high-resolution mass spectrometry (GC-HRMS). The limit of detection (LOD) was in the range of 0.3 to 1.5 pg/g. OCs concentrations in maternal samples (serum) of INMA and RHEA were determined according to Goñi et al ^41^ with a LOD of 67.0 pg/g and Koponen et al ^42^ with LODs between 1.7 and 14.3 pg/g, respectively. We also calculated the sum of PCBs by summing the concentrations of the 5 PCBs in pg/g. In the current manuscript we use the 5 individual PCBs, not the sum.

*Brominated compounds (PBDEs)*

Concentrations of PBDEs were determined in serum or plasma following the method described in Caspersen et al ^40^ also using GC-MS/MS for detection. The LOD ranged from 0.15 to 0.3 pg/g. In RHEA only PBDE-47 was determined in maternal samples (serum) following the method described in Koponen et al ^42^ with a LOD of 2.85pg/g.

*Perfluorinated alkylated substances (PFAS)*

Concentrations of PFASs were determined in serum or plasma using the method by Haug et al ^43^, while the method by Poothong et al ^44^ was applied for the whole blood samples. The LOD was 0.02 µg/L for all PFASs. In the majority of INMA maternal samples (plasma), PFASs were determined according to Manzano-Salgado et al ^45^ and with LODs between 0.05 and 0.1 µg/L. Only five maternal samples from INMA were analyzed at NIPH. In order to know whether concentrations measured in both labs were comparable we performed an inter-laboratory comparison of 10 samples with low to high PFOS concentrations as reference selected from all analyzed in the Institute for Occupational Medicine, RWTH Aachen University (Germany) ^45^. NIPH was blinded to the concentrations of samples. PFOS and PFHxS plasma concentrations determined in both laboratories were highly correlated (Spearman r=0.83 and 0.93, respectively) whereas PFOA and PFNA were less correlated (Spearman r=0.70 and 0.55, respectively). The three samples with low PFOS concentrations had levels between the LOD and the LOQ or close to the LOQ for PFHxS, PFOA, and PFNA. Considering that concentrations between the LOD and the LOQ have higher uncertainty, we excluded these samples and the spearman correlations became higher: PFOA r=0.96, PFHxS r=0.93, and PFNA r=0.86. Due to the high correlations the NIPH concentrations for subjects included in the comparison have been used. For the PFASs, 1:1 ratios were assumed for serum and plasma, while 1:2 ratios were used for whole blood vs serum/plasma ^46^. Thus, for PFASs all whole blood concentrations were multiplied by two.

*Metals and essential elements*

Concentrations of 15 metals and elements in whole blood were performed at ALS Scandinavia, Sweden according to Rodushkin et al ^47^. The LOD ranged from 0.003–3.03 µg/L except for sodium (Na), potassium (K) and magnesium (Mg) for which the LOD ranges from 0.06-0.15 mg/L. Mercury in INMA was determined in cord whole blood following the procedure described in Ramon et al ^48^ with a LOD of 2.0 μg/L. Cord blood Hg concentrations were be divided by 1.7 to be comparable with maternal whole blood concentrations ^49^. Ten of these metals and elements (Hg, Cd, Pb, As, Cs, Cu, Tl, Mn, Co, and Mo) were included in the exposome analyses because of their potential toxicity. Zn, Na, K, Mg, and Se were not considered toxic and were included as covariates. This classification was based on expert judgment (Joan Grimalt, personal communication) and literature review ^50^.

*Phthalate metabolites*

Concentrations of ten phthalate metabolites were determined in urine according to Sabaredzovic et al ^51^. The LOD ranged from 0.06 to 0.61μg/L. In the majority of INMA maternal samples, phthalates were determined according to Valvi et al ^52^ with LOD ranged from 0.5-1.0 μg/L except 37 INMA samples that were analyzed at NIPH. For comparability, we analyzed 10 samples with low to high monoethyl phthalate (MEP) concentrations as reference selected from all analyzed in the Bioanalysis Research Group at the Hospital del Mar Medical Research Institute (Barcelona, Spain) ^52^. NIPH was blinded to the concentrations of samples. Urinary concentrations of the phthalate metabolites determined in both laboratories were highly correlated (Spearman ranging from r=0.69 to 0.97). Due to the high correlations the

NIPH concentrations for subjects included in the comparison have been used. We also calculated the total concentration of di-2-ethylhexyl phthalate (DEHP) by summing the molar concentrations of mono-2-ethylhexyl phthalate (MEHP), mono-2-ethyl-5-hydroxyhexyl phthalate (MEHHP), mono-2-ethyl-5-oxohexyl phthalate (MEOHP), and mono-2-ethyl 5-carboxypentyl phthalate (MECPP). The molar concentrations (in µmol/L) were calculated by dividing the concentration of every metabolite by its molecular weight. In the current manuscript we do not use the sum variables.

*Phenols*

Concentrations of phenols were determined in urine according to Sakhi et al ^53^ with the LOD ranged from 0.03-0.06 μg/L. In EDEN, phthalate metabolites were determined in urine samples according to Philippat et al ^54^ with the LOD ranged from 0.2-2.3 μg/L. We performed an inter-lab comparison of 12 samples selected from all analyzed in the I National Center for Environmental Health laboratory at the CDC in Atlanta, Georgia, USA ^54^. NIPH was blinded to the concentrations of samples. Phenols urinary concentrations determined in both laboratories were strongly correlated (Spearman ranging from r=0.90 to 1.0). Due to the high correlations the NIPH concentrations for subjects included in the comparison have been used.

*Organophosphate (OP) pesticide metabolites*

Analysis of OP pesticide metabolites in urine was made according to Cequier et al ^55^ and with the LOD ranged from 0.06-0.36μg/L. DMDTP in children was detected in less than 20% of samples and DEDTP in children and mothers was detected in less than 2% of samples; therefore, categorical variables were created categorizing urinary DMTDP and DEDTP levels as detected or not detected considering the limits of detection of 0,19 and 0,05 μg/L, respectively. However, the DEDTP variable in mothers and children and the DMDTP variable in mothers had too few subjects in the “detected” category (less than 30) and were finally removed from the exposome analyses.

*Cotinine*

Concentrations of cotinine in urine were determined using The Immulite® 2000 Nicotine Metabolite (Cotinine) 600 Test on an Immulite 2000 XPi from Siemens Healthineers at Fürst Medisinsk Laboratorium, Norway. The LOD was 3.03μg/L. Cotinine in maternal urine samples from INMA were determined according to Aurrekoetxea et al ^56^ and with a LOD of 1.21μg/L. We performed an interlab-comparison of 10 urine samples with low to high cotinine concentrations selected from all analyzed in the Public Health Laboratory of Bilbao - LSPPV (Spain) ^56^. NIPH was blinded to the concentrations of samples. Cotinine urinary concentrations determined in both laboratories were highly correlated (Spearman r=0.95).

For maternal smoking, a categorical variable was created based on the urinary cotinine levels to distinguish non-smokers, second-hand-tobacco smokers, and smokers ^57^:

- Non-smokers: values <LOD or cotinine levels <18.5 µg/L
- Second-hand-tobacco smokers: cotinine levels ≥18.5-50 µg/L
- Smokers: cotinine levels >50 µg/L

In the children, a categorical variable was created categorizing urinary cotinine levels as detected or not detected considering the limit of detection of 3.03μg/L.

*Adjustments for creatinine*

Concentrations of creatinine in urine were performed on an AU680 Chemistry System form Beckman Coulter using DRI® Creatinine-Detect® Test at Fürst Medisinsk Laboratorium, Norway with a LOD of 0,03mmol/L. Creatinine in maternal samples of INMA and EDEN were determined by using the Jaffé method - Beckman Coulter© AU5400 and an enzymatic reaction using a Roche Hitachi 912 chemistry analyzer (Roche Hitachi, Basel, Switzerland), respectively. Urinary concentrations of phthalate metabolites, phenols, OP pesticide metabolites, and cotinine were adjusted in respect to creatinine and expressed in μg/g of creatinine.

# eMethods 5. Creation of the general health score

For each health domain (cardiometabolic, respiratory/allergy and mental), a sub-score was created. Following the approach of Eisenmann (2008), subscores were defined as the sum of z-scores or multiple factor analysis (applying equal weighting to predefined groups of variables). All of the three sub-scores were built such that a high score means the child is in good health on this specific domain.

*Creation of z-scores*

Continuous health parameters were transformed in z-scores, using Generalize Additive Model for Location, Scale and Shape (GAMLSS) ^59^ to standardize on covariates and approach normality. The standardisation was done on age and stratified by sex for all parameters except for the FEV_1_ % pred given that it already considers them by construct. For BPs, a standardization for height was added ^60^ (see eTable 5 and 6).

*Cardiometabolic subscore*

As used previously in the Helix population, the cardiometabolic sub-score was defined as (-z waist circumference) + (- z insulin) + (z HDL cholesterol – z triglycerides)/2 + (-z systolic BP – z diastolic BP)/2 ^61,62^.

*Respiratory/allergy subscore*

The respiratory/allergy sub-score was defined as the first principal component of the multiple factorial analysis. This approach was used because, unlike the sum of z-scores, it can be used with mixed-type variables. This factorial analysis considered a first group of variables with asthma, allergies, eczema and rhinitis and another group with FEV_1_% pred. The coordinates and contribution of each variable on the first component is available in eTable 7. The coordinates of asthma and allergy-related variables are negatives, which means that a child with many allergy-related outcomes should be at the right sight of the first axis. On the contrary, the negative coordinate of the lung function (FEV1) means that a child with a good lung function should be at the left side of the first axis. This is coherent with the inverse correlation between spirometry results (FEV_1_% pred) and allergy-related outcomes. Overall, it means that a child with a good lung function and few allergy-related outcomes also tend to have a high respiratory/allergy sub-score.

*Mental subscore*

The mental sub-score was defined as the first principal component of the multiple factorial analysis. This approach was used because, to the best of our knowledge, no mental scores based on z-scores was found in the literature. This factorial analysis considered the z-scores of behavioural parameters (ADHD, internalizing, externalizing) in one group and the cognitive parameter (fluid intelligence) in another group. The coordinates and contribution of each variable on the first component is available in eTable 8. The coordinates of behavioural scores (ADHD, internalizing, externalizing) are positive, which means that a child with high behavioural scores (more behavioural troubles) should be at the right sight of the first axis. On the contrary, the negative coordinate of the cognitive score (fluid intelligence) means that a child with a high cognitive score (good cognition) should be at the left side of the first axis. This is coherent with the inverse correlation between behavioral scores and cognitive results. Overall, it means that a child with high cognition and few behavioral troubles also tend to have a high mental sub-score.

*General health score*

The three sub-scores were scaled and aggregated into one general health score by taking their mean. By construct, the general health score is low for children with conjointly low-to-moderate cardiometabolic, respiratory/allergy and mental health in children, as well as for children highly affected in one health domain while no or moderately affected for the other two.

# eMethods 6. Data pre-processing

For each variable, the optimal transformation to approach normality was chosen using a Box-Cox power transformation approach and described in eTable 9. When normality could not be achieved by transformation, the variable was categorized. For biomarker exposures, values under the limit of detection were imputed using a distribution-based method ^63^. To improve comparison between exposures, estimates reported for continuous variables are expressed as an increase in interquartile range (IQR) (see eTable 9).

*Imputation process*

To prevent losing information and introducing potential selection biases, missing values of exposures and confounders were imputed. When possible, we relied on the multiple imputation procedure, which is a commonly used and accepted method to deal with missing data ^64^. Multiple imputation provides valid inferences under the missing at random (MAR) assumption, which assumes that missing data are associated to observed variables and not to unobserved information. In multiple imputation, missing values are imputed stochastically several times. Imputing missing values several times allows the quantification of the uncertainty in results associated with imputation, and to account for this uncertainty in the final standard errors, confidence intervals and p-values.

For the imputation process, continuous variables should have a normal distribution. As mentioned above, skewed exposure variables were transformed to achieve normality or categorized if no transformation worked. The distributions of all transformed variables were examined to make sure that transformations did not lead to extreme/influential observations. In cases of variables with zeros that required a log transformation, a constant value was added to the variable as the log of zero is minus infinity. The constant value was chosen to minimize the skewness of the resulting variable. Exposures with more than 30% of missing values were excluded from the process.

Missing values of exposures and adjustment variables were imputed using the method of chained equations (White, Royston, and Wood 2011), using the mice package in R (Buuren and Groothuis-Oudshoorn 2011). The large number of variables involved in the HELIX analyses implies that not all exposures can be used as predictors in imputation models, as imputation models would be too large, and therefore reduced imputation models had to be specified. It is recommended that imputation models include between 10 and 25 variables (Buuren and Groothuis-Oudshoorn 2011). We used the quickpred function to reduce the number of predictors. With this function, potential predictors for a given variable var1 were restricted to those that had: 1) an absolute correlation greater than 0.2 with var1 or with the binary variable indicating if var1 is missing (mincor=0.2); and 2) a proportion of non-missing observations greater than 40% among the observations with missing values in var1 (minpuc=0.4). Values for mincor and minpucwere tuned to have a number of predictors between 5 and 25 for all variables. If some variables continued to have too many predictors, they were reduced manually. In addition, we forced into the imputation models cohort and one representative outcome variable of each the main groups of health outcomes that will be subsequently analysed in the HELIX project (namely asthma as representative of respiratory health; z-score of BMI as representative of cardio metabolic outcomes; and number of correct responses in RAVEN test and total problems in CBCL as representative of neurodevelopment). Those variables were included with the include option. With the procedures described, the imputation process still produced some errors, which were solved on a case-by-case basis. Mostly, this consisted in removing from the predictor set of a given variable highly correlated variables. The method of predictive mean matching was used for all continuous exposures. Twenty imputed datasets were created.

After imputation, the following diagnostics were conducted. All variables with missing values were inspected. In particular, the imputed and non-missing observations were compared using density plots and stripplots of Van Buuren (2011). These types of comparison were only done when there were more than 5% of the observations with missing values. Numerically, variables were flagged if they have 1) an absolute difference between means of the observed and imputed values greater than 2 standard deviations; or 2) a ratio of variances of the observed and imputed values that is less than 0.5 or greater than 2 ^64^. For categorical variables, those with a significant chi-squared test between imputed and non-imputed were examined. If variables were flagged and imputations seem implausible, the predictors included in the imputation model for that variable were changed to those plausible imputations were generated.

Under the missing at random hypothesis, **imputing the outcomes would not benefit the model compared to an analysis on complete cases** ^65^**.** Therefore, all participants with at least one missing value on the health parameters were removed, leading to the inclusion of 870 mother-child pairs.

# eMethods 7. Exposome-health association study

The statistical methods were identified a priori through a series of simulation studies mimicking as closely as possible the situation expected with Helix data ^66^. Two approaches were retained.

First, the LASSO algorithm was used for testing the association of multiple environmental exposures with the general health score (multi-pollutant model). LASSO considers all exposures simultaneously ^53^⁠ and performs variable selection through estimates’ shrinkage (i.e. the lowest regression coefficients corresponding to the least informative predictors are assigned a zero value). LASSO models using linear regression were computed using the *glmnet* function in the glmnet R package ^67^. We determined the overall penalty parameter, lambda, by maximizing the prediction log-likelihood using 10-fold cross-validation (function *cv.glmnet*), applied on the 20 imputed datasets separately. To identify the final exposures selected, we applied LASSO penalized models with the selected lambdas on the 20 imputed datasets separately, and kept the variables selected for at least 10 datasets out of the 20 (50%). To obtain final effect estimates, we fitted a linear regression (*lm*) that was simultaneously adjusted for all exposure variables selected by LASSO (after checking for potential collinearity) plus our set of *a priori* confounders. Finally, we removed all exposures with p-value over 10% one by one, removing each time the exposure with the highest p-value.

Then, exposome-wide association study (ExWAS) analyses were used to perform exposure-by-exposure estimation of the association with the general health score, using linear regression adjusted for confounders using the *lm* function in the R. To account for multiple comparisons, a family wise error rate correction was used to correct the p-value threshold ^68^. The correction uses a Bonferroni procedure extended to handle correlated tests: the actual number of exposures being tested (M) is replaced by a smaller value called the effective number of independent exposures (Me). Me is estimated by ∑i=1 M [I(λi >1)(λi − 1)], where I(x) is an indicator function and λi are the eigenvalues of the matrix of correlations between M exposures. The p value threshold to control FWER to α, using Me in a Bonferroni procedure, is then α /Me.

Both statistical methods (ExWAS and LASSO) were performed separately for the prenatal and the childhood exposomes and were adjusted for potential confounders. Fixed effects were used for all confounders including the cohort. Rubin’s rules were used to aggregate the results from the 20 imputed datasets ^69^. The final model consisted in a multivariable linear regression considering all the selected exposures with LASSO, using the Rubin’s rule to pool the results from the 20 imputed datasets. All exposures that were not significant at the 10% risk level were removed one by one, removing each time the exposure with the highest p-value.

The two approaches retained have each their advantages and drawbacks and both are complementary. ExWAS allows screening all individual exposures, but does not take into account potential confounding between them and is at higher risk of false positive selection with our data structure ^66^. On the other hand, LASSO - a variable selection method which aims to optimally predict the outcome – has the advantage that it mutually adjusts for the effect of other exposures, but will tend to select only one predictor in the case when a true predictor is highly correlated with other exposures (e.g., within an exposure family) ^66^.

# eTable 1. Collection time points of health parameters in childhood (mean, SD)

|  | **Cohort** | | | | | |
| --- | --- | --- | --- | --- | --- | --- |
|  | **BiB** | **EDEN** | **KANC** | **INMA** | **MoBa** | **RHEA** |
| **Child age** (years) | 6.6 (0.2) | 10.8 (0.6) | 6.5 (0.5) | 8.8 (0.6) | 8.5 (0.5) | 6.5 (0.3) |

Abbreviations: SD: standard deviation

# eTable 2. Biological matrices of maternal and child samples

| **Chemicals** | **Cohort** | | | | | |
| --- | --- | --- | --- | --- | --- | --- |
|  | **BiB** | **EDEN** | **KANC** | **INMA** | **MoBa** | **RHEA** |
| **OCs and PBDEs** | | | | | | |
| Mother | serum/plasma | serum | - | serum | plasma | serum |
| Child | serum | serum | serum | serum | serum | serum |
| **PFASs** |  |  |  |  |  |  |
| Mother | serum/plasma | serum | whole blood | plasma | plasma | serum |
| Child | plasma | plasma | plasma | plasma | plasma | plasma |
| **Metals** |  |  |  |  |  |  |
| Mother | whole blood | whole blood | whole blood | cord whole blood | whole blood | whole blood |
| Child | whole blood | whole blood | whole blood | whole blood | whole blood | whole blood |
| **Phthalate metabolites, phenols, OP pesticide metabolites, cotinine, and creatinine** | | | | | | |
| Mother | urine | urine | - | urine | urine | urine |
| Child | urine | urine | urine | urine | urine | urine |
| **Lipids** |  |  |  |  |  |  |
| Mother | serum/plasma | serum | - | serum | plasma | serum |
| Child | plasma | plasma | Plasma | plasma | plasma | plasma |

Abbreviations: **OC**: organochlorine; **OP**: organophosphate pesticides; **PBDEs**: polybrominateddiphenyl ethers; **PFASs**: per- and polyfluoroalkyl substances.

# eTable 3. Collection time points of prenatal exposures

| **Types of exposure** | **Time of assessment during pregnancy** |
| --- | --- |
| Urban exposures | Mean of the assessment at each of the 3 trimesters |
| Lifestyle / Social environment | Whole pregnancy   - Tobacco: any consumption during pregnancy - Diet: average consumption in times/week |
| Biomarkers | See eTable 4 |

# eTable 4. Collection time points of maternal blood and urine samples (mean, SD)

|  | **Cohort** | | | | | |
| --- | --- | --- | --- | --- | --- | --- |
|  | **BiB** | **EDEN** | **KANC** | **INMA** | **MoBa** | **RHEA** |
| **Gestational weeks** | 26.6 (1.4) | 26.1 (1.2) | 39.4 (1.3) | 13.7 (2.0) / 34.2 (1.3) ^a^ | 18.7 (0.9) | 14.1 (3.7) |

Abbreviations: SD: standard deviation

^a^In INMA, blood was collected in the first trimester whereas urine was collected in the third trimester of pregnancy.

# eTable 5. Standardization of the cardiometabolic parameters

|  | Distribution | Sex | $\mu$  Location | $log(\sigma)$  Scale | $\nu$  Skewness | $log(\tau)$  Kurtosis |
| --- | --- | --- | --- | --- | --- | --- |
| Waist circumference | BCT | Girls | CS(age) | age | 1 | age |
|  |  | Boys | age | age | 1 | age |
| Insulin (Log) | BCT | Girls | CS(age) | age | 1 | 1 |
|  |  | Boys | CS(age) | age | 1 | 1 |
| Triglycerides | BCCG | Girls | 1 | 1 | 1 | - |
|  |  | Boys | 1 | 1 | 1 | - |
| HDL cholesterol | BCPE | Girls | CS(age) | age | 1 | 1 |
|  |  | Boys | CS(age) | age | 1 | 1 |
| Systolic blood pressure | BCT | Girls | age + height | 1 | 1 | 1 |
|  |  | Boys | $log \left( \mu\right)$ ~ age + height | 1 | 1 | 1 |
| Diastolic blood pressure | BCT | Girls | age + height | 1 | age | 1 |
|  |  | Boys | $log \left( \mu\right)$ ~ age + height | 1 | 1 | 1 |

Method: for each health outcome, a distribution is chosen among the one proposed by the package GAMLSS (Box-Cox, Weibull, binomial etc), and its parameters (location, scale, skewness, kurtosis) are additive functions of the covariates. The distribution and the function for the parameters were defined in the IDEFICS study ^60,70–72^. A stratification on sex is used. The estimation of the parameters and the creation of the z-scores are done via the package GAMLSS in R. Acronyms: BCT=Box-Cox t, BCCG=Box-Cox Cole Green, BCPE=Box-Cox Power Exponential, CS = cubic splines. Example on how to read this table: for the waist circumference, a Box Cox t distribution has been chosen, with $\mu$ being a function of the cubic spline of age, $loglog (\sigma$) a function of age and $loglog \left( \tau\right)$ a function of age.

# eTable 6. Standardization of the mental and cognitive parameters

|  | Distribution | Stratified on sex? | $\mu$  Location | $log(\sigma)$  Scale | $\nu$  Skewness | $log(\tau)$  Kurtosis |
| --- | --- | --- | --- | --- | --- | --- |
| ADHD | ZINBI | Yes | $log \left( \mu\right) \sim$ age | age | $\nu\sim age$ | - |
| Internalizing | ZINBI | Yes | $log \left( \mu\right)$ $\sim$ age | age | $\nu\sim age$ | - |
| Externalizing | ZINBI | Yes | $log \left( \mu\right)$ $\sim$ age | age | $\nu\sim age$ | - |
| Fluid intelligence | BCPE | Yes | age | age | age | age |

Method: for each health outcome, a distribution is chosen among the one proposed by the package GAMLSS (Box-Cox, Weibull, binomial etc), and its parameters (location, scale, skewness, kurtosis) are additive functions of the covariates. The best distribution was chosen by minimizing the AIC (see the distribution chosen in this table). The estimation of the parameters and the creation of the z-scores are done via the package GAMLSS in R. Acronyms: ZINBI=Zero-Inflated Negative Binomial, BCPE=Box-Cox Power Exponential.

# eTable 7. Factor analysis on respiratory and allergy-related parameters – Dimension 1

|  | **Coordinates** | **Contribution** | **Cos2** |
| --- | --- | --- | --- |
| FEV-1 % pred | 0.88 | 73.77 | 0.77 |
| Asthma (no) | 0.19 | 1.83 | 0.34 |
| Asthma (yes) | -1.34 | 13.17 | 0.34 |
| Rhinitis (no) | 0.17 | 1.31 | 0.13 |
| Rhinitis (yes) | -0.54 | 4.23 | 0.13 |
| Eczema (no) | 0.15 | 1.06 | 0.12 |
| Eczema (yes) | -0.56 | 3.96 | 0.12 |
| Food allergy (no) | 0.04 | 0.07 | 0.02 |
| Food allergy (yes) | -0.31 | 0.60 | 0.02 |

The first dimension (used as the respiratory/allergy sub-score) represented 29% of variance. The coordinates of asthma and allergy-related variables are negatives, which means that a child with many allergy-related outcomes should be at the right sight of the first axis. On the contrary, the negative coordinate of the lung function (FEV1) means that a child with a good lung function should be at the left side of the first axis. This is coherent with the inverse correlation between spirometry results (FEV_1_% pred) and allergy-related outcomes. Overall, it means that a child with a good lung function and few allergy-related outcomes also tend to have a high respiratory/allergy sub-score.

# eTable 8. Factor analysis on mental and cognitive parameters – Dimension 1

|  | **Coordinates** | **Contribution** | **Cos2** |
| --- | --- | --- | --- |
| z-ADHD | 0.62 | 15.75 | 0.38 |
| z-Internalizing | 0.61 | 15.30 | 0.37 |
| z-Externalizing | 0.68 | 18.95 | 0.46 |
| z- fluid intelligence | -0.76 | 50.00 | 0.58 |

The first dimension represented 48% of variance, with high values corresponding to a high cognitive results and low behavioural indexes. The coordinates of behavioral scores (ADHD, internalizing, externalizing) are positive, which means that a child with high behavioral scores (more behavioral troubles) should be at the right sight of the first axis. On the contrary, the negative coordinate of the cognitive score (fluid intelligence) means that a child with a high cognitive score (high cognition) should be at the left side of the first axis. This is coherent with the inverse correlation between behavioral scores and cognitive results. Overall, it means that a child with high cognition and few behavioral troubles also tend to have a high mental sub-score.

# eTable 9. Transformation applied to continuous postnatal exposures and IQR

| Variable | Transformation | IQR^a^ |
| --- | --- | --- |
| KIDMED |  | 2.00 |
| sleep |  | 0.93 |
| Physical activity |  | 32.76 |
| Sedentary activity |  | 130.71 |
| NO2 - home | Log | 0.59 |
| PM10 - home | - | 10.93 |
| PM2.5 - home | Log | 3.13 |
| PM2.5abs - home | - | 0.19 |
| NO2 - school | Log | 0.54 |
| PM 2.5 - school | Log | 3.00 |
| PM 2.5 abs - school | - | 0.60 |
| Temperature | - | 9.36 |
| Humidity | - | 15.91 |
| Uv | - | 2.16 |
| NDVI - home | - | 0.26 |
| NDVI - school | - | 0.21 |
| Population density - home | Sqrt | 51.70 |
| Population density - school | Sqrt | 45.97 |
| Built density - home | Sqrt | 183.52 |
| Built density - school | Sqrt | 138.99 |
| Connect density - home | Sqrt | 0.73 |
| Connect density - school | Sqrt | 0.60 |
| Bststop density - home | Log | 1.25 |
| Bststop density - school | Log | 1.10 |
| Facility richness - home | - | 0.11 |
| Facility richness - school | - | 0.11 |
| Facility density - home | - | 1.47 |
| Facility density - school | - | 1.39 |
| SEI - home | - | 0.18 |
| SEI - school | - | 0.19 |
| walkability - home | - | 0.15 |
| walkability - school | - | 0.13 |
| Traffic load - home | ^(1/3) | 120.46 |
| Traffic nearest road - home | ^(1/3) | 13.67 |
| Inverse distance to nearest road - home | Log | 1.44 |
| Traffic load - school | ^(1/3) | 119.25 |
| Traffic nearest road - school | ^(1/3) | 12.23 |
| Inverse distance to nearest road - school | Log | 0.91 |
| TEX indoor | Log | 0.44 |
| NO2 indoor | Log | 1.27 |
| Benzene indoor | Log | 0.31 |
| PM 2.5 indoor | Log | 0.34 |
| PM absorbance indoor | Log | 0.35 |
| BPA | Log | 0.99 |
| BUPA | Log | 1.18 |
| OXBE | Log | 3.01 |
| TRCS | Log | 2.22 |
| ETPA | Log | 0.95 |
| MEPA | Log | 2.18 |
| MBZP | Log | 0.94 |
| SumDEHP | Log | 0.89 |
| MEP | Log | 2.21 |
| MIBP | Log | 1.50 |
| MNBP | Log | 1.32 |
| SumDINP | Log | 1.34 |
| K | Log | 0.11 |
| Mg | Log | 0.12 |
| Na | Log | 0.09 |
| Se | Log | 0.18 |
| Zn | Log | 0.17 |
| Cd | Log | 0.78 |
| Co | Log | 0.39 |
| Cu | Log | 0.16 |
| Cs | Log | 0.43 |
| Hg | Log | 1.37 |
| Mn | Log | 0.31 |
| Mo | Log | 0.44 |
| Pb | Log | 0.45 |
| PFHXS | Log | 1.58 |
| PFNA | Log | 1.25 |
| PFOA | Log | 0.72 |
| PFOS | Log | 1.24 |
| PBDE47 | Log | 0.71 |
| DEP | Log | 2.39 |
| DMTP | Log | 1.61 |
| DDE | Log | 1.25 |
| HCB | Log | 0.50 |
| SumPCB | Log | 0.96 |

^a^ IQR: Interquartile range (Q75%-Q25%), calculated based on the 20 stacked imputed datasets (the original dataset was not included). Standardisation on IQR was applied after the transformation (log, Log etc) by removing the mean and dividing by the IQR.

# eTable 10. Description of transformed urban exposures

| Exposure | Unit | Period | N (%) | Min, or % | Q1 | Median | Q3 | Max |
| --- | --- | --- | --- | --- | --- | --- | --- | --- |
| **Outdoor air pollution** | | | | | | | | |
| NO2 | μg/m3 | Pregnancy | 799 (91.8%) | 3,1 | 3,33 | 3,51 | 3,71 | 4,67 |
| PM10 | μg/m3 | Pregnancy | 791 (90.9%) | 8,1 | 16,8 | 23,8 | 29,2 | 47,7 |
| PM2.5 | μg/m3 | Pregnancy | 854 (98.2%) | 7,5 | 13 | 14,9 | 17 | 21,7 |
| PM2.5 absorbance | 10-5m-1 | Pregnancy | 743 (85.4%) | 0,55 | 0,79 | 0,93 | 1,21 | 1,89 |
| NO2 - home | μg/m3 | Childhood | 867 (99.7%) | 2,62 | 3,17 | 3,53 | 3,75 | 4,56 |
| NO2 - school | μg/m3 | Childhood | 864 (99.3%) | 2,66 | 3,22 | 3,54 | 3,75 | 4,5 |
| PM10 - home | μg/m3 | Childhood | 756 (86.9%) | 11,2 | 15,9 | 27,5 | 32,3 | 47,7 |
| PM2.5 - home | μg/m3 | Childhood | 867 (99.7%) | 4,8 | 12 | 13,8 | 15,2 | 21,8 |
| PM2.5 - school | μg/m3 | Childhood | 864 (99.3%) | 4,9 | 11,9 | 13,8 | 15 | 21,2 |
| PM2.5 abs - home | 10-5m-1 | Childhood | 614 (70.6%) | 0,44 | 0,67 | 0,77 | 0,89 | 1,58 |
| PM2.5 abs - school | 10-5m-2 | Childhood | 611 (70.2%) | 0,6 | 0,88 | 1,24 | 1,47 | 2,75 |
|  |  |  |  |  |  |  |  |  |
| **Indoor air pollution** | | | |  |  |  |  |  |
| NO2 indoor | μg/m3 | Childhood | 1152 (88.5%) | 23.6 | 37.34 | 55.7 | 129.02 | 1224.2 |
| TEX indoor | μg/m3 | Childhood | 1163 (89.4%) | 20.5 | 27.94 | 34.47 | 43.38 | 154.47 |
| Benzene indoor | μg/m3 | Childhood | 1219 (93.7%) | 2.1 | 2.75 | 3.19 | 3.71 | 23.57 |
| PM2.5 indoor | μg/m3 | Childhood | 1221 (93.9%) | 12.6 | 15.64 | 17.46 | 21.98 | 196.37 |
| PM absorbance indoor | μg/m3 | Childhood | 1212 (93.2%) | 0.97 | 1.16 | 1.34 | 1.65 | 30.57 |
|  |  |  |  |  |  |  |  |  |
| **Meteorology** |  |  |  |  |  |  |  |  |
| Pressure |  | Pregnancy | 997 (76.6%) | 975 | 981 | 1000.1 | 1003 | 1015.5 |
| Temperature | °C | Pregnancy | 1199 (92.2%) | 1.2 | 8.5 | 10.4 | 14.5 | 22.6 |
| Humidity | % | Pregnancy | 1199 (92.2%) | 55.8 | 69.9 | 75.9 | 85.4 | 90.8 |
| Temperature - home | °C | Childhood | 1202 (92.4%) | -3.5 | 6.5 | 11.9 | 16 | 27.3 |
| Humidity - home | % | Childhood | 1202 (92.4%) | 52.1 | 67.3 | 74.4 | 83.1 | 96.1 |
| UV - home | kJ / m2 | Childhood | 1295 (99.5%) | 0 | 0.23 | 1.02 | 2.4 | 5.25 |
|  |  |  |  |  |  |  |  |  |
| **Surrounding natural spaces** | | | | | | | | |
| NDVI < 100 m | - | Pregnancy | 1279 (98.3%) | 0.11 | 0.25 | 0.41 | 0.52 | 0.74 |
| Greenspace < 300 m | - | Pregnancy | 1188 (91.3%) | 75% | - | | | |
| Bluespace < 300 m | - | Pregnancy | 1188 (91.3%) | 8% | - | | | |
| NDVI < 100 m - home | - | Childhood | 1297 (99.7%) | 0.09 | 0.32 | 0.49 | 0.58 | 0.81 |
| NDVI < 100 m - school | - | Childhood | 1291 (99.2%) | 0.1 | 0.31 | 0.45 | 0.52 | 0.76 |
| Greenspace < 300 m - home | - | Childhood | 1194 (91.8%) | 79% | - | | | |
| Greenspace < 300 m - school | - | Childhood | 1185 (91.1%) | 78% | - | | | |
| Bluespace < 300 m - home | - | Childhood | 1194 (91.8%) | 8% | - | | | |
| Bluespace < 300 m - school | - | Childhood | 1185 (91.1%) | 7% | - | | | |
|  |  |  |  |  |  | | | |
| **Built environment** |  |  |  |  |  |  |  |  |
| Population density - home | people/km2 | Childhood | 1299 (99.8%) | 3 | 1 116 | 4 570 | 7 225 | 68 382 |
| Population density - school | people/km2 | Childhood | 1293 (99.4%) | 0 | 1 521 | 4 802 | 7 225 | 44 521 |
| Population density - home | built/km2 | Childhood | 1157 (88.9%) | 412 | 86 201 | 144 476 | 228 962 | 649 314 |
| Population density - school | built/km2 | Childhood | 1185 (91.1%) | 41 | 106 146 | 152 022 | 230 112 | 648 186 |
| Connectivity density - home | intersect/km2 | Childhood | 1162 (89.3%) | 74 | 143 | 213 | 293 | 821 |
| Connectivity density - school |  | Childhood | 1179 (90.6%) | 89 | 171 | 230 | 302 | 804 |
| BST stop density - home | n/km2 | Childhood | 1131 (86.9%) | 7 | 7 | 14 | 25 | 107 |
| BST stop density - school | n/km2 | Childhood | 1123 (86.3%) | 4 | 7 | 14 | 21 | 60 |
| Facility richness - home | - | Childhood | 1299 (99.8%) | 0 | 0 | 0.04 | 0.11 | 0.33 |
| Facility richness - school | - | Childhood | 1293 (99.4%) | 0 | 0.04 | 0.07 | 0.14 | 0.33 |
| Landuse Shannon's Evenness Index - home | - | Childhood | 1196 (91.9%) | 0 | 0.31 | 0.4 | 0.49 | 0.7 |
| Landuse Shannon's Evenness Index - school | - | Childhood | 1185 (91.1%) | 0.08 | 0.33 | 0.43 | 0.52 | 0.73 |
| Walkability index - home | - | Childhood | 1109 (85.2%) | 0.1 | 0.25 | 0.3 | 0.4 | 0.6 |
| Walkability index - school | - | Childhood | 1117 (85.9%) | 0.15 | 0.28 | 0.32 | 0.4 | 0.62 |
| Pop density | people/km2 | Pregnancy | 1278 (98.2%) | 0 | 2 673 | 5 520 | 9 565 | 68 382 |
| Building density | m2 built/km2 | Pregnancy | 1193 (91.7%) | 121 | 112 426 | 164 430 | 264 093 | 652 218 |
| Connectivity density | intersect/km2 | Pregnancy | 1203 (92.5%) | 4 | 88 | 164 | 250 | 745 |
| BST stop density | n/km2 | Pregnancy | 1124 (86.4%) | 4 | 7 | 14 | 25 | 93 |
| Facility richness | - | Pregnancy | 1237 (95.1%) | 0 | 0.02 | 0.07 | 0.14 | 0.42 |
| Landuse Shannon's Evenness Index | - | Pregnancy | 1188 (91.3%) | 0 | 0.33 | 0.42 | 0.51 | 1 |
| Walkability index | - | Pregnancy | 1105 (84.9%) | 0.1 | 0.22 | 0.28 | 0.32 | 0.62 |
| **Noise** |  |  |  |  |  |  |  |  |
| Lden - home | dB(A) | Childhood | 887 (68.2%) | 21 | 50 | 55.5 | 62 | 76 |
| Lden - school | dB(A) | Childhood | 883 (67.9%) | 32.2 | 50 | 52 | 61.4 | 76 |
| Ln - home | dB(A) | Childhood | 524 (40.3%) | 34.1 | 50 | 50 | 52 | 68 |
| Ln - school | dB(A) | Childhood | 523 (40.2%) | 31.3 | 50 | 50 | 50 | 68 |
| Lden | dB(A) | Pregnancy | 899 (69.1%) | 33.5 | 50 | 57.4 | 63 | 77.4 |
| Ln | dB(A) | Pregnancy | 526 (40.4%) | 32.8 | 50 | 50 | 53.6 | 69.8 |
|  |  |  |  |  |  |  |  |  |
| **Water DBPs** |  |  |  |  |  |  |  |  |
| THM | μg/L | Pregnancy | 1112 (85.5%) | 4.1 | 8.17 | 17.81 | 48.42 | 157.59 |
| Brominated THMs | μg/L | Pregnancy | 1112 (85.5%) | 0.52 | 1.04 | 1.65 | 16.28 | 135.64 |
| Chloroform | μg/L | Pregnancy | 1112 (85.5%) | 0.55 | 1.09 | 4.14 | 13.6 | 46.99 |
|  |  |  |  |  |  |  |  |  |
| **Traffic** |  |  |  |  |  |  |  |  |
| Traffic load all roads - home | veh / day m | Childhood | 1257 (96.6%) | 0 | 0 | 241 804 | 1 749 690 | 25 308 602 |
| Traffic load all roads - school |  | Childhood | 1237 (95.1%) | 0 | 0 | 258 475 | 1 647 213 | 20 956 092 |
| Traffic density nearest road - home | veh / day | Childhood | 1283 (98.6%) | 0 | 593 | 3 308 | 10941 | 119 823 |
| Traffic density nearest road - school |  | Childhood | 1105 (84.9%) | 0 | 831 | 3582 | 9938 | 134218 |
| Inverse distance nearest road - home | m-1 | Childhood | 1283 (98.6%) | 0.01 | 0.01 | 0.02 | 0.06 | 99.48 |
| Inverse distance nearest road - school |  | Childhood | 1283 (98.6%) | 0.01 | 0.01 | 0.02 | 0.04 | 2.72 |
| Traffic load all roads | veh / day m | Pregnancy | 985 (75.7%) | 0 | 0 | 296 741 | 2 131 747 | 25 490 056 |
| Traffic density nearest road | veh / day | Pregnancy | 1281 (98.5%) | 0 | 493 | 1 093 | 4020 | 60 698 |
| Traffic density nearest road | m-1 | Pregnancy | 1280 (98.4%) | 0.02 | 0.04 | 0.07 | 0.12 | 16.44 |

Population: Helix population, n=1301. Acronyms: N=number of individuals without missing values. %=percentage of individuals without missing values over the whole Helix sample (n=1301). Min=minimum. Q1=first quantile. Q3=third quantile. Max = maximum. Max=maximum.

# eTable 11. Description of exposures regarding the lifestyle

| Variable name | Units | Period | N (%) before imputation | Min, or % | | | Q1 | Median | Q3 | | Max |  |  |
| --- | --- | --- | --- | --- | --- | --- | --- | --- | --- | --- | --- | --- | --- |
| **Tobacco** |  |  |  |  | | | | | | | | |  |
| Maternal active smoking | Factor | Pregnancy | 1259 (96.8%) | Yes, n = 190 (15.1%) | | | | | | | | |  |
| Maternal passive smoking | - | Pregnancy | 1279 (98.3%) | Yes, n = 515 (40.3%) | | | | | | | | |  |
| Smoking status of parents (both) | - | Childhood | 1286 (98.8%) | Neither, n = 800 (62.2%) | | | | | | | | |  |
|  |  |  |  | One, n = 350 (27.2%) | | | | | | | | |  |
|  | - |  |  | Both, n = 136 (10.6%) | | | | | | | | |  |
| Exposure to smoke | - | Childhood | 1271 (97.7%) | Exposure, n = 445 (35%) | | | | | | | | |  |
|  |  |  |  |  | | | | | | | | |  |
| **Sleep** |  |  |  |  | | |  |  |  | |  |  |  |
| Sleep duration | - | Childhood | 1290 (99.2%) | 7.6 | | | 9.8 | 10.3 | 10.7 | | 12.9 |  |  |
|  |  |  |  |  | | |  |  |  | |  |  |  |
| **Physical activity** | | |  |  | | | | | | | | |  |
| Moderate activity | times/week | Pregnancy | 790 (60.7%) | None or sometimes, n = 251 (31.8%) | | | | | | | | |  |
|  |  |  |  | Often, n = 303 (38.4%) | | | | | | | | |  |
|  |  |  |  | Very Often, n = 236 (29.9%) | | | | | | | | |  |
| Vigorous activity | times/week | Pregnancy | 772 (59.3%) | Medium-High, n = 286 (37%) | | | | | | | | |  |
| Physical activity | min/day | Childhood | 1036 (79.6%) | -27.8 | | | 21.6 | 35.9 | 54.6 | | 146.8 |  |  |
| Sedentary activity | min/day | Childhood | 1283 (98.6%) | 3.1 | | | 152.1 | 210 | 282.9 | | 994.3 |  |  |
| pet | - | Childhood | 1292 (99.3%) | Yes, n = 485 (37.5%) | | | | | | | | |  |
|  |  |  |  |  | | | | | | | | |  |
| **Socio-economic situation** | | | | | |  | | | | | | | |
| Family Affluence Scale |  | Childhood | 1293 (99.4%) | Low, n = 136 (10.5%) | | | | | | | | |  |
|  |  |  |  | Middle, n = 499 (38.6%) | | | | | | | | |  |
|  |  |  |  | High, n = 658 (50.9%) | | | | | | | | |  |
| Participation in organization |  | Childhood | 1222 (93.9%) | None, n = 680 (55.6%) | | | | | | | | |  |
|  |  |  |  | 1 organisation, n = 350 (28.6%) | | | | | | | | |  |
|  |  |  |  | 2 or more organisations, n = 192 (15.7%) | | | | | | | | |  |
| Contact with family | - | Childhood | 1281 (98.5%) | Less than once a week, n = 63 (4.9%) | | | | | | | | |  |
|  |  |  |  | Once a week, n = 370 (28.9%) | | | | | | | | |  |
|  |  |  |  | (almost) Daily, n = 848 (66.2%) | | | | | | | | |  |
| House crowding | Number of people | Pregnancy | 1067 (82%) | 0 | | | 2 | 3 | 3 | | 11 |  |  |
|  |  |  |  |  | | |  |  |  | |  |  |  |
| **Diet** |  |  |  |  | | |  |  |  | |  |  |  |
| KIDMED index | - | Childhood | 1290 (99.2%) | -3 | | | 2 | 3 | 4 | | 9 |  |  |
| Sweets | times/week | Childhood | 1288 (99%) | <4 times/week, n = 434 (33.7%) | | | | | | | | |  |
|  |  |  |  | 4-8.5 times/week, n = 428 (33.2%)  >8.5 times/week, n = 426 (33.1%) | | | | | | | |  |  |
|  |  |  |  |  |  |  |  |  |  |  |  |  |  |
| Meat | - | Childhood | 1289 (99.1%) | <6 times/week, n = 491 (38.1%)  6-9 times/week, n = 360 (27.9%)  >9 times/week, n = 438 (34%) | | | | | | | |  |  |
|  |  |  |  |  |  |  |  |  |  |  |  |  |  |
|  |  |  |  |  |  |  |  |  |  |  |  |  |  |
| Processed meat | - | Childhood | 1289 (99.1%) | <1.5 times/week, n = 470 (36.5%)  1.5-4 times/week, n = 525 (40.7%)  >4 times/week, n = 294 (22.8%) | | | | | | |  |  |  |
|  |  |  |  |  |  |  |  |  |  |  |  |  |  |
|  |  |  |  |  |  |  |  |  |  |  |  |  |  |
| Fish/ Seafood | - | Childhood | 1289 (99.1%) | <1.5 times/week, n = 500 (38.8%)  1.5-3 times/week, n = 400 (31%)  >3 times/week, n = 389 (30.2%) | | | | | | | |  |  |
|  |  |  |  |  |  |  |  |  |  |  |  |  |  |
|  |  |  |  |  |  |  |  |  |  |  |  |  |  |
| Yogurt | - | Childhood | 1289 (99.1%) | <3 times/week, n = 564 (43.8%)  3-7 times/week, n = 291 (22.6%)  >7 times/week, n = 434 (33.7%) | | | | | | | |  |  |
|  |  |  |  |  |  |  |  |  |  |  |  |  |  |
|  |  |  |  |  |  |  |  |  |  |  |  |  |  |
| Beverages (sodas) | - | Childhood | 1288 (99%) | <1times/month, n = 488 (37.9%)  1-times/month – 1 times/week, n = 478 (37.1%)  >1times/week, n = 322 (25%) | | | | | | | |  |  |
|  |  |  |  |  |  |  |  |  |  |  |  |  |  |
|  |  |  |  |  |  |  |  |  |  |  |  |  |  |
| Bread | - | Childhood | 1288 (99%) | <7 times/week, n = 572 (44.4%)  7-14 times/week, n = 388 (30.1%)  >14 times/week, n = 328 (25.5%) | | | | | | | |  |  |
|  |  |  |  |  |  |  |  |  |  |  |  |  |  |
|  |  |  |  |  |  |  |  |  |  |  |  |  |  |
| Breakfast cereals | - | Childhood | 1289 (99.1%) | <1.5 times/week, n = 444 (34.4%)  1.5-5.5 times/week, n = 420 (32.6%)  >5.5 times/week, n = 425 (33%) | | | | | | | |  |  |
|  |  |  |  |  |  |  |  |  |  |  |  |  |  |
|  |  |  |  |  |  |  |  |  |  |  |  |  |  |
| Potatoes | - | Childhood | 1288 (99%) | <3 times/week, n = 469 (36.4%)  3-4 times/week, n = 555 (43.1%)  >4 times/week, n = 264 (20.5%) | | | | | | |  |  |  |
|  |  |  |  |  |  |  |  |  |  |  |  |  |  |
|  |  |  |  |  |  |  |  |  |  |  |  |  |  |
| Vegetables | - | Childhood | 1288 (99%) | <6 times/week, n = 634 (49.2%)  6-9 times/week, n = 241 (18.7%)  >9 times/week, n = 413 (32.1%) | | | | | | | |  |  |
|  |  |  |  |  |  |  |  |  |  |  |  |  |  |
|  |  |  |  |  |  |  |  |  |  |  |  |  |  |
| Dairy | - | Childhood | 1289 (99.1%) | <15 times/week, n = 429 (33.3%)  15-26 times/week, n = 430 (33.4%)  >26 times/week, n = 430 (33.4%) | | | | | | | |  |  |
|  |  |  |  |  |  |  |  |  |  |  |  |  |  |
|  |  |  |  |  |  |  |  |  |  |  |  |  |  |
| Fruits | - | Childhood | 1287 (98.9%) | <7 times/week, n = 431 (33.5%)  7-17 times/week, n = 426 (33.1%)  >17 times/week, n = 430 (33.4%) | | | | | | | |  |  |
|  |  |  |  |  |  |  |  |  |  |  |  |  |  |
|  |  |  |  |  |  |  |  |  |  |  |  |  |  |
| Cereals | - | Childhood | 1287 (98.9%) | <14 times/week, n = 426 (33.1%)  14-23 times/week, n = 426 (33.1%)  >23 times/week, n = 435 (33.8%) | | | | | | | |  |  |
|  |  |  |  |  |  |  |  |  |  |  |  |  |  |
|  |  |  |  |  |  |  |  |  |  |  |  |  |  |
| Bakery products | - | Childhood | 1289 (99.1%) | <2 times/week, n = 477 (37%) | | | | | | |  |  |  |
|  |  |  |  | 2-6 times/week, n = 481 (37.3%)  >6 times/week, n = 331 (25.7%) | | | | | | |  |  |  |
|  |  |  |  |  |  |  |  |  |  |  |  |  |  |
| Added lipids | - | Childhood | 1289 (99.1%) | <3 times/week, n = 491 (38.1%)  3-7 times/week, n = 373 (28.9%)  >7 times/week, n = 425 (33%) | | | | | | | |  |  |
|  |  |  |  |  |  |  |  |  |  |  |  |  |  |
|  |  |  |  |  |  |  |  |  |  |  |  |  |  |
| Caffeinated drinks | - | Childhood | 1286 (98.8%) | Never, n = 796 (61.9%)  1 times/month, n = 185 (14.4%)  >1 times/month, n = 305 (23.7%) | | | | | | |  |  |  |
|  |  |  |  |  |  |  |  |  |  |  |  |  |  |
|  |  |  |  |  |  |  |  |  |  |  |  |  |  |
| Fast food | - | Childhood | 1287 (98.9%) | <1 times/month, n = 734 (57%) | | | | | | | | |  |
|  |  |  |  | 1-2 times/month, n = 410 (31.9%)  >2 times/month, n = 143 (11.1%) | | | | | | | |  |  |
|  |  |  |  |  |  |  |  |  |  |  |  |  |  |
| Organic food | - | Childhood | 1287 (98.9%) | <1 time/month, n = 428 (33.3%)  1 time/month – 1 time/week, n = 534 (41.5%)  >1 time/week, n = 325 (25.3%) | | | | | | | |  |  |
|  |  |  |  |  |  |  |  |  |  |  |  |  |  |
|  |  |  |  |  |  |  |  |  |  |  |  |  |  |
| Ready-made meal | - | Childhood | 1287 (98.9%) | <1 times/month, n = 619 (48.1%)  1 times/month – 1 times/week, n = 341 (26.5%)  >1 times/week, n = 327 (25.4%) | | | | | | | |  |  |
|  |  |  |  |  |  |  |  |  |  |  |  |  |  |
|  |  |  |  |  |  |  |  |  |  |  |  |  |  |
| Meat | - | Pregnancy | 940 (72.3%) | <7 times/week, n = 313 (33.7%) | | | | | | |  |  |  |
|  |  |  |  | 7-10 times/week, n = 303 (32.6%) | | | | | | |  |  |  |
|  |  |  |  | >10 times/week, n = 313 (33.7%) | | | | | | |  |  |  |
| Fish/ Seafood | - | Pregnancy | 827 (63.6%) | <2 times/week, n = 325 (34.6%) | | | | | | | |  |  |
|  |  |  |  | 2-4 times/week, n = 296 (31.5%) | | | | | | | |  |  |
|  |  |  |  | >4 times/week, n = 319 (33.9%) | | | | | | |  |  |  |
| Vegetables | - | Pregnancy | 832 (64%) | <9 times/week, n = 277 (33.5%) | | | | | | |  |  |  |
|  |  |  |  | 9-16 times/week, n = 269 (32.5%) | | | | | | | |  |  |
|  |  |  |  | >16 times/week, n = 281 (34%) | | | | | | |  |  |  |
| Fruits | - | Pregnancy | 829 (63.7%) | <10 times/week, n = 275 (33.1%) | | | | | | |  |  |  |
|  |  |  |  | 10-18 times/week, n = 274 (32.9%) | | | | | | |  |  |  |
|  |  |  |  | | >18 times/week, n = 283 (34%) | | | | |  |  |  |  |
| Dairy | - | Pregnancy | 832 (64%) | <17 times/week, n = 274 (33.1%) | | | | | | |  |  |  |
|  |  |  |  | 17-27 times/week, n = 273 (32.9%) | | | | | | |  |  |  |
|  |  |  |  | >27 times/week, n = 282 (34%) | | | | | | | | |  |
| Legumes | - | Pregnancy | 944 (72.6%) | <0.5 times/week, n = 280 (33.7%) | | | | | | | |  |  |
|  |  |  |  | 0.5-2 times/week, n = 304 (36.5%) | | | | | | |  |  |  |
|  |  |  |  | >2 times/week, n = 248 (29.8%) | | | | | | | |  |  |
| Cereals | - | Pregnancy | 457 (35.1%) | <9 times/week, n = 313 (33.2%) | | | | | | |  |  |  |
|  |  |  |  | 9-27 times/week, n = 310 (32.8%) | | | | | | |  |  |  |
|  |  |  |  | >27 times/week, n = 321 (34%) | | | | | | | | |  |
| Fast food | - | Pregnancy | 457 (35.1%) | <1 times/month, n = 229 (50.1%) | | | | | | | |  |  |
|  |  |  |  | 1 times/month, n = 74 (16.2%) | | | | |  | |  |  |  |
|  |  |  |  | >1 times/month, n = 154 (33.7%) | | | | | | |  |  |  |
| Folic Acid | - | Pregnancy | 875 (67.3%) | No, n = 386 (44.1%) | | | | | | |  |  |  |
|  |  |  |  | Yes, n = 489 (55.9%) | | | | |  | |  |  |  |

Population: Helix population, n=1301. Acronyms: N=number of individuals without missing values. %=percentage of individuals without missing values over the whole Helix sample (n=1301). Min=minimum. Q1=first quantile. Q3=third quantile. Max = maximum. Max=maximum.

# eTable 12. Description of biomarkers in the Helix population

| Exposure | Unit | Period | N (%) before imputation | % quantifiable | | Min | | Q1 | | Median | | Q3 | | | Max | | |  |
| --- | --- | --- | --- | --- | --- | --- | --- | --- | --- | --- | --- | --- | --- | --- | --- | --- | --- | --- |
| **Phthlates** |  |  |  |  | |  | |  | |  | |  | | |  | | |  |
| MBzP | μg/g | Childhood | 1300 (99.9%) | 99.70% | | 3.7 | | 6 | | 8 | | 11.5 | | | 354.6 | | |  |
| MECPP | μg/g | Childhood | 1300 (99.9%) | 99.90% | | 27.7 | | 43.1 | | 56.9 | | 81 | | | 3691 | | |  |
| MEHHP | μg/g | Childhood | 1298 (99.8%) | 100% | | 15.7 | | 24.4 | | 32.2 | | 45.3 | | | 2257 | | |  |
| MEHP | μg/g | Childhood | 1260 (96.8%) | 99.50% | | 2 | | 3.4 | | 4.6 | | 6.8 | | | 284 | | |  |
| MEOHP | μg/g | Childhood | 1300 (99.9%) | 100% | | 9.8 | | 15.3 | | 20.3 | | 28.4 | | | 1296 | | |  |
| MEP | μg/g | Childhood | 1301 (100%) | 99.00% | | 3.4 | | 16.4 | | 33.6 | | 76.1 | | | 3191.5 | | |  |
| MiBP | μg/g | Childhood | 1301 (100%) | 99.90% | | 5 | | 26 | | 41.6 | | 73 | | | 861.1 | | |  |
| MnBP | μg/g | Childhood | 1301 (100%) | 100% | | 2.1 | | 15.3 | | 23.9 | | 38.3 | | | 487.8 | | |  |
| Oh-MINP | μg/g | Childhood | 1301 (100%) | 92.60% | | 0.6 | | 3.4 | | 5.4 | | 9.2 | | | 548.7 | | |  |
| Oxo-MINP | μg/g | Childhood | 1301 (100%) | 95.70% | | 0.5 | | 1.9 | | 2.8 | | 4.9 | | | 680.3 | | |  |
| MBzP | μg/g | Pregnancy | 1088 (83.6%) | 99.90% | | 3.9 | | 7.3 | | 10.9 | | 18.9 | | | 781.4 | | |  |
| MECPP | μg/g | Pregnancy | 913 (70.2%) | 99.90% | | 27.9 | | 44.9 | | 56.1 | | 74.5 | | | 1379.6 | | |  |
| MEHHP | μg/g | Pregnancy | 1089 (83.7%) | 99.80% | | 11.2 | | 21.1 | | 28.6 | | 41.6 | | | 975.5 | | |  |
| MEHP | μg/g | Pregnancy | 1085 (83.4%) | 96.80% | | 4.8 | | 8.9 | | 13.2 | | 19.7 | | | 421.7 | | |  |
| MEOHP | μg/g | Pregnancy | 1089 (83.7%) | 99.90% | | 8.9 | | 16.6 | | 22.3 | | 32 | | | 792.4 | | |  |
| MEP | μg/g | Pregnancy | 1080 (83%) | 100% | | 73 | | 145 | | 250.7 | | 541.2 | | | 17805 | | |  |
| MiBP | μg/g | Pregnancy | 1088 (83.6%) | 100% | | 25.3 | | 46.5 | | 61.8 | | 83.9 | | | 729.1 | | |  |
| MnBP | μg/g | Pregnancy | 1089 (83.7%) | 100% | | 18.9 | | 36.8 | | 47.8 | | 65.8 | | | 6472 | | |  |
| Oh-MINP | μg/g | Pregnancy | 914 (70.3%) | 100% | | 0.6 | | 1.2 | | 1.5 | | 2.1 | | | 67.2 | | |  |
| Oxo-MINP | μg/g | Pregnancy | 914 (70.3%) | 100% | | 0.6 | | 1.2 | | 1.7 | | 2.4 | | | 75.6 | | |  |
|  |  |  |  |  | |  | |  | |  | |  | | |  | | |  |
| **Phenols** |  |  |  |  | |  | |  | |  | |  | | |  | | |  |
| BPA | μg/g | Pregnancy | 1084 (83.3%) | 99.40% | | 1.6 | | 3.1 | | 4.4 | | 8.1 | | | 108.4 | | |  |
| BUPA | μg/g | Pregnancy | 1083 (83.2%) | 97.00% | | 0.4 | | 0.7 | | 3.7 | | 14.6 | | | 372.2 | | |  |
| ETPA | μg/g | Pregnancy | 817 (62.8%) | 97.40% | | 1.1 | | 2.3 | | 7.4 | | 27.9 | | | 6793.8 | | |  |
| MEPA | μg/g | Pregnancy | 815 (62.6%) | 99.80% | | 40.5 | | 79.3 | | 206.5 | | 427.6 | | | 39239 | | |  |
| OXBE | μg/g | Pregnancy | 1085 (83.4%) | 98.50% | | 1.5 | | 2.9 | | 6.4 | | 28.8 | | | 12854 | | |  |
| PRPA | μg/g | Pregnancy | 1083 (83.2%) | 97.30% | | 8.9 | | 17.8 | | 53.1 | | 143 | | | 12503 | | |  |
| TRCS | μg/g | Pregnancy | 1085 (83.4%) | 99.30% | | 1.5 | | 3 | | 7.8 | | 81 | | | 1652 | | |  |
| BPA | μg/g | Childhood | 1289 (99.1%) | 98.30% | | 2.4 | | 4.8 | | 6.5 | | 9.6 | | | 364.6 | | |  |
| BUPA | μg/g | Childhood | 1296 (99.6%) | 96.60% | | 0 | | 0.1 | | 0.1 | | 0.2 | | | 97 | | |  |
| ETPA | μg/g | Childhood | 1298 (99.8%) | 99.30% | | 0.4 | | 0.9 | | 1.1 | | 1.6 | | | 2033.9 | | |  |
| MEPA | μg/g | Childhood | 1299 (99.8%) | 99.70% | | 3.3 | | 6.5 | | 9.8 | | 29.7 | | | 23987 | | |  |
| OXBE | μg/g | Childhood | 1301 (100%) | 100% | | 0 | | 0.9 | | 2.2 | | 6.9 | | | 7968 | | |  |
| PRPA | μg/g | Childhood | 1284 (98.7%) | 67.30% | | 0 | | 0 | | 0.3 | | 1.7 | | | 1782.9 | | |  |
| TRCS | μg/g | Childhood | 1301 (100%) | 99.90% | | 0.1 | | 0.3 | | 0.6 | | 1.5 | | | 724.1 | | |  |
|  |  |  |  |  | |  | |  | |  | |  | | |  | | |  |
| **Organochlorines compounds (OCs)** | | | | | | | | | | | | |  | | |  | | |
| DDE | pg/g | Pregnancy | 1048 (80.6%) | 99.90% | | 388 | | 675 | | 1024 | | 1663 | | | 21618 | | |  |
| DDT | pg/g | Pregnancy | 855 (65.7%) | 65.60% | | 10.9 | | 21.7 | | 27.7 | | 56.1 | | | 1120.6 | | |  |
| HCB | pg/g | Pregnancy | 1048 (80.6%) | 99.10% | | 73 | | 143 | | 178.5 | | 232.3 | | | 1488.9 | | |  |
| PCB 118 | pg/g | Pregnancy | 858 (65.9%) | 79.10% | | 20.4 | | 38.1 | | 55.3 | | 84.4 | | | 600.5 | | |  |
| PCB 138 | pg/g | Pregnancy | 1048 (80.6%) | 96.50% | | 76.6 | | 125.4 | | 174.9 | | 256 | | | 1951 | | |  |
| PCB 153 | pg/g | Pregnancy | 1048 (80.6%) | 99.60% | | 146 | | 245.6 | | 340.1 | | 494.6 | | | 3213.7 | | |  |
| PCB 170 | pg/g | Pregnancy | 855 (65.7%) | 99.50% | | 24.4 | | 47.8 | | 69.6 | | 117 | | | 1438.2 | | |  |
| PCB 180 | pg/g | Pregnancy | 1048 (80.6%) | 97.60% | | 75.1 | | 136.2 | | 196.7 | | 290 | | | 3444.3 | | |  |
| DDE | pg/g | Childhood | 1296 (99.6%) | 99.90% | | 141 | | 237.2 | | 335.5 | | 564.2 | | | 24662 | | |  |
| DDT | pg/g | Childhood | 1296 (99.6%) | 100.00% | | 63.1 | | 123.6 | | 143 | | 174.9 | | | 867.1 | | |  |
| HCB | pg/g | Childhood | 1296 (99.6%) | 99.90% | | 33.8 | | 66.7 | | 87.4 | | 119.4 | | | 2120.2 | | |  |
| PCB 118 | pg/g | Childhood | 1296 (99.6%) | 99.80% | | 99.7 | | 143 | | 184.8 | | 257.8 | | | 2195 | | |  |
| PCB 138 | pg/g | Childhood | 1296 (99.6%) | 99.80% | | 16.1 | | 31.3 | | 53.1 | | 96.3 | | | 657.1 | | |  |
| PCB 153 | pg/g | Childhood | 1296 (99.6%) | 100% | | 2.9 | | 5.6 | | 9.8 | | 18.8 | | | 2256.7 | | |  |
| PCB 170 | pg/g | Childhood | 1296 (99.6%) | 90.70% | | 15 | | 29.7 | | 35 | | 43.4 | | | 1314.2 | | |  |
| PCB 180 | pg/g | Childhood | 1296 (99.6%) | 99.20% | | 5.7 | | 11.2 | | 18.3 | | 33.4 | | | 230.7 | | |  |
| PBDE 153 | Pg/g | Pregnancy | 648 (49.8%) | 72.90% | | 0.4 | | 0.6 | | 6.5 | | 9.5 | | | 2721.1 | | |  |
| PBDE 47 | Pg/g | Pregnancy | 684 (52.6%) | 80.90% | | 4 | | 7.7 | | 9.8 | | 14.7 | | | 675.6 | | |  |
| PBDE 153 | Pg/g | Childhood | 1279 (98.3%) | 54.40% | | 0.4 | | 0.5 | | 1.9 | | 4.4 | | | 135.3 | | |  |
| PBDE 47 | Pg/g | Childhood | 1279 (98.3%) | 90.80% | | 1.6 | | 3.1 | | 3.8 | | 5.1 | | | 342.5 | | |  |
|  |  |  |  |  | |  | |  | |  | |  | | |  | | |  |
| **Per- and poly-fluoroalkyl substance (PFAS)** | | | | | | | | | | | | | | | |  | | |
| PFHXS | µg/L | Pregnancy | 1240 (95.3%) | 97.50% | | 0.4 | | 0.6 | | 0.9 | | 1.2 | | | 21.1 | | |  |
| PFNA | µg/L | Pregnancy | 1240 (95.3%) | 97.90% | | 0.4 | | 0.9 | | 1.1 | | 1.5 | | | 6.4 | | |  |
| PFOA | µg/L | Pregnancy | 1240 (95.3%) | 100% | | 1.4 | | 2.8 | | 3.7 | | 4.7 | | | 33.1 | | |  |
| PFOS | µg/L | Pregnancy | 1240 (95.3%) | 100% | | 4.4 | | 8.3 | | 10.6 | | 13.7 | | | 52 | | |  |
| PFUNDA | µg/L | Pregnancy | 1032 (79.3%) | 95.40% | | 0.1 | | 0.2 | | 0.3 | | 0.4 | | | 2.8 | | |  |
| PFHXS | µg/L | Childhood | 1301 (100%) | 99.70% | | 0 | | 0.2 | | 0.4 | | 0.6 | | | 27.9 | | |  |
| PFNA | µg/L | Childhood | 1301 (100%) | 99.50% | | 0 | | 0.3 | | 0.5 | | 0.8 | | | 11.3 | | |  |
| PFOA | µg/L | Childhood | 1301 (100%) | 99.80% | | 0.2 | | 1.2 | | 1.5 | | 2 | | | 6.7 | | |  |
| PFOS | µg/L | Childhood | 1301 (100%) | 99.80% | | 0.2 | | 1.4 | | 2.2 | | 3.4 | | | 34.1 | | |  |
| PFUNDA | µg/L | Childhood | 1301 (100%) | 68.60% | | 0 | | 0 | | 0 | | 0.1 | | | 1.5 | | |  |
|  |  |  |  |  | |  | |  | |  | |  | | |  | | |  |
| **Metals and essential minerals** | | | | | | | | | | |  | |  | | |  | | |
| K | µg/L | Pregnancy | 833 (64%) | 100% | | 2896 | | 3565 | | 3565 | | 3821 | | | 7131 | | |  |
| Mg | µg/L | Pregnancy | 833 (64%) | 100% | | 39.1 | | 50.9 | | 53.8 | | 56.5 | | | 77.7 | | |  |
| Na | µg/L | Pregnancy | 833 (64%) | 100% | | 2896 | | 3565 | | 3565 | | 3821 | | | 4390 | | |  |
| Se | µg/L | Pregnancy | 833 (64%) | 100% | | 111.4 | | 147 | | 157.6 | | 170.1 | | | 249 | | |  |
| Zn | µg/L | Pregnancy | 833 (64%) | 100% | | 6208 | | 8780 | | 10085 | | 10809 | | | 37640 | | |  |
| As | µg/L | Pregnancy | 833 (64%) | 58.50% | | 0.4 | | 0.6 | | 1.5 | | 2.5 | | | 90.5 | | |  |
| Cd | µg/L | Pregnancy | 833 (64%) | 99.60% | | 0.2 | | 0.3 | | 0.4 | | 0.5 | | | 27.9 | | |  |
| Co | µg/L | Pregnancy | 833 (64%) | 100% | | 0.2 | | 0.3 | | 0.3 | | 0.4 | | | 5.7 | | |  |
| Cu | µg/L | Pregnancy | 833 (64%) | 100% | | 1782.9 | | 2521 | | 2702 | | 2896 | | | 3565 | | |  |
| Cs | µg/L | Pregnancy | 833 (64%) | 100% | | 1.6 | | 2.4 | | 2.8 | | 3.3 | | | 12.1 | | |  |
| Hg | µg/L | Pregnancy | 1020 (78.4%) | 98.80% | | 1.1 | | 2 | | 2.9 | | 4.4 | | | 44.6 | | |  |
| Mn | µg/L | Pregnancy | 833 (64%) | 100% | | 11.6 | | 16.9 | | 19.6 | | 22.8 | | | 57.7 | | |  |
| Mo | µg/L | Pregnancy | 833 (64%) | 100% | | 0.6 | | 1 | | 1.1 | | 1.3 | | | 70.5 | | |  |
| Pb | µg/L | Pregnancy | 833 (64%) | 100% | | 9.4 | | 14.3 | | 16.8 | | 20.4 | | | 194 | | |  |
| K | µg/L | Childhood | 1298 (99.8%) | 100% | | 3104 | | 3821 | | 4096 | | 4096 | | | 5792 | | |  |
| Mg | µg/L | Childhood | 1298 (99.8%) | 100% | | 53.1 | | 58.9 | | 61.4 | | 64 | | | 86.8 | | |  |
| Na | µg/L | Childhood | 1298 (99.8%) | 100% | | 2195 | | 3565 | | 3565 | | 3821 | | | 4705 | | |  |
| Se | µg/L | Childhood | 1298 (99.8%) | 100% | | 128 | | 166 | | 178.5 | | 190 | | | 238 | | |  |
| Zn | µg/L | Childhood | 1298 (99.8%) | 100% | | 5404 | | 7643 | | 8192 | | 8780 | | | 15286 | | |  |
| As | µg/L | Childhood | 1298 (99.8%) | 67.10% | | 0.4 | | 0.6 | | 1.6 | | 2.6 | | | 64 | | |  |
| Cd | µg/L | Childhood | 1298 (99.8%) | 86.50% | | 0 | | 0.1 | | 0.1 | | 0.1 | | | 1.9 | | |  |
| Co | µg/L | Childhood | 1298 (99.8%) | 99.90% | | 0.2 | | 0.3 | | 0.3 | | 0.4 | | | 2.8 | | |  |
| Cu | µg/L | Childhood | 1298 (99.8%) | 100% | | 1351 | | 1663 | | 1663 | | 1782 | | | 5404 | | |  |
| Cs | µg/L | Childhood | 1298 (99.8%) | 100% | | 1.4 | | 2.1 | | 2.4 | | 2.8 | | | 9.4 | | |  |
| Hg | µg/L | Childhood | 1298 (99.8%) | 97.70% | | 0.4 | | 0.8 | | 1.3 | | 2.2 | | | 20.5 | | |  |
| Mn | µg/L | Childhood | 1298 (99.8%) | 100% | | 10.4 | | 14.2 | | 15.7 | | 17.6 | | | 34.8 | | |  |
| Mo | µg/L | Childhood | 1298 (99.8%) | 99.50% | | 0.6 | | 1.2 | | 1.4 | | 1.6 | | | 35.5 | | |  |
| Pb | µg/L | Childhood | 1298 (99.8%) | 100% | | 8.5 | | 12.8 | | 14.9 | | 17.5 | | | 219.8 | | |  |
| Tl | - | Childhood | 1298 (99.8%) | 7.20% | | Detected, n = 93 (7.2%) | | | | | | | | | | |  |  |
|  |  |  |  |  | |  | | | | | | | | | | |  |  |
| **Organophosphate pesticides (OP)** | | | | |  | |  | |  | |  | |  | | |  | | |
| DEP | μg/g | Pregnancy | 1082 (83.2%) | 97.80% | | 1.9 | | 3.7 | | 5.2 | | 8.3 | | | 199.5 | | |  |
| DETP | μg/g | Pregnancy | 1037 (79.7%) | 50.00% | | 0.1 | | 0.3 | | 0.7 | | 2.6 | | | 45.3 | | |  |
| DMP | μg/g | Pregnancy | 1080 (83%) | 90.80% | | 4.2 | | 8.3 | | 12.5 | | 20.5 | | | 326.3 | | |  |
| DMTP | μg/g | Pregnancy | 1084 (83.3%) | 88.90% | | 2.1 | | 4.1 | | 7 | | 14.4 | | | 221.3 | | |  |
| DMDTP | μg/g | Pregnancy | 969 (74.5%) | 41.60% | | 0.2 | | 0.3 | | 0.3 | | 5 | | | 588.1 | | |  |
| DEP | μg/g | Childhood | 1299 (99.8%) | 80.90% | | 0.5 | | 1 | | 2.3 | | 5 | | | 666.3 | | |  |
| DETP | μg/g | Childhood | 1280 (98.4%) | 43.30% | | 0.1 | | 0.2 | | 0.3 | | 1.9 | | | 78.8 | | |  |
| DMP | μg/g | Childhood | 1295 (99.5%) | 49.30% | | 0.4 | | 0.6 | | 1.1 | | 5 | | | 83.3 | | |  |
| DMTP | μg/g | Childhood | 1300 (99.9%) | 90.40% | | 1.3 | | 2.5 | | 4.3 | | 7.8 | | | 407.3 | | |  |
| DMDTP | - | Childhood | 1300 (99.9%) | 18.20% | | Detected, n = 237 (18.2%) | | | | | | | |  | | |  |  |
|  |  |  |  |  | |  | | | | | | | |  | | |  |  |
| **Tobacco** |  |  |  |  | |  | | | | | | | | | | |  |  |
| Cotinine | - | Pregnancy | 1093 (84%) | - | | 18.5-50 µg/L, n = 137 (12.5%) | | | | | | | | | | |  |  |
|  |  |  |  |  | | >50 µg/L, n = 186 (17%) | | | | | | | | | | |  |  |
| Cotinine | - | Childhood | 1301 (100%) | - | | Detected, n = 237 (18.2%) | | | | | | | |  | | |  |  |

Population: Helix population, n=1301. Acronyms: N=number of individuals without missing values. %=percentage of individuals without missing values over the whole Helix sample (n=1301). Min=minimum. Q1=first quantile. Q3=third quantile. Max = maximum. Max=maximum.

# eTable 13. Description of the health parameters considered

| Variable name | Health domain | N (%) | Min | Q1 | Median | Q3 | Max |
| --- | --- | --- | --- | --- | --- | --- | --- |
| HDL cholesterol | Cardiometabolic | 1284 (98.7%) | 0.7 | 1.3 | 1.5 | 1.7 | 2.9 |
| Triglycerides | Cardiometabolic | 1284 (98.7%) | 0.28 | 0.66 | 0.85 | 1.15 | 4.37 |
| Insulin ^a^ | Cardiometabolic | 1170 (89.9%) | 89 | 160 | 208 | 307 | 2740 |
| Systolic blood pressure | Cardiometabolic | 1298 (99.8%) | 71 | 92 | 99 | 107 | 159 |
| Diastolic blood pressure | Cardiometabolic | 1298 (99.8%) | 37 | 52.5 | 57 | 62.5 | 118.5 |
| Circumference of the waist | Cardiometabolic | 1299 (99.8%) | 21 | 54 | 57 | 62 | 93 |
| ADHD | Mental-Cognition | 1292 (99.3%) | 0 | 2 | 6 | 11 | 35 |
| Internalizing | Mental-Cognition | 1294 (99.5%) | 0 | 2 | 5 | 9 | 42 |
| Externalizing | Mental-Cognition | 1294 (99.5%) | 0 | 2 | 5 | 10 | 38 |
| Raven score | Mental-Cognition | 1292 (99.3%) | 9 | 22 | 27 | 32 | 36 |
| FEV1-pred | Respiratory - Allergies | 1033 (79.4%) | 60.9 | 89.6 | 98.6 | 107.4 | 139.2 |
| Variable name |  | N (%) | Modality, n (%) | | | | |
| Rhinitis | Respiratory - Allergies | 1295 (99.5%) | Yes, n=324 (25%) | | | | |
| Eczema | Respiratory - Allergies | 1288 (99%) | Yes, n=270 (21%) | | | | |
| Food allergy | Respiratory - Allergies | 1286 (98.8%) | Yes, n=129 (10%) | | | | |
| Asthma | Respiratory - Allergies | 1292 (99.3%) | Yes, n=155 (12%) | | | | |

Population: Helix population, n=1301 children. Acronyms: N=number of individuals without missing values. %=percentage of individuals without missing values over the whole Helix sample (n=1301). Min=minimum. Q1=first quantile. Q3=third quantile. Max = maximum.

^a^ Insulin levels had a skewed distribution and were log-transformed before statistical analysis (back-transformed in this table).

# eTable 14. Correlations between the three sub-scores

|  | Cardiometabolic subscore | Respiratory subscore | Mental subscore |
| --- | --- | --- | --- |
| Cardiometabolic subscore | - | -0.03 | 0.12 |
| Respiratory subscore | - | - | 0.01 |
| Mental subscore | - | - | - |

Method: pearson coefficients of correlation between pairwise complete observations.

# eTable 15. Description of the general health score by covariates

| Categorical covariates ^a^ | N (%) | General health score | | |  |  |
| --- | --- | --- | --- | --- | --- | --- |
|  |  | mean | sd | p-value ^b^ |  |  |
| Cohort |  |  |  | <0.01 | |  |
| BiB | 134 (15%) | -0.20 | 0.59 |  | |  |
| EDEN | 112 (13%) | -0.26 | 0.50 |  | |  |
| INMA | 160 (18%) | -0.04 | 0.59 |  | |  |
| KANC | 129 (15%) | 0.23 | 0.52 |  | |  |
| MoBA | 193 (22%) | 0.39 | 0.55 |  | |  |
| RHEA | 142 (16%) | -0.10 | 0.56 |  | |  |
| Child sex |  |  |  | 0.29 | |  |
| Girls | 411 (47%) | 0.06 | 0.58 |  | |  |
| Boys | 459 (53%) | 0.01 | 0.62 |  | |  |
| Highest parental education |  |  |  | <0.01 | |  |
| Primary | 79 (9%) | -0.20 | 0.65 |  | |  |
| Secondary | 262 (30%) | -0.07 | 0.58 |  | |  |
| Higher | 529 (61%) | 0.12 | 0.59 |  | |  |
| Parents from the cohort country |  |  |  | 0.12 | |  |
| None | 101 (12%) | 0.01 | 0.67 |  | |  |
| One | 37 (4%) | -0.16 | 0.60 |  | |  |
| Both | 732 (84%) | 0.05 | 0.59 |  | |  |
| Season of birth |  |  |  | 0.53 | |  |
| Winter | 181 (21%) | 0.04 | 0.55 |  | |  |
| Spring | 186 (21%) | 0.08 | 0.59 |  | |  |
| Summer | 244 (28%) | 0.00 | 0.66 |  | |  |
| Autumn | 259 (30%) | 0.02 | 0.59 |  | |  |
| Breastfeeding duration |  |  |  | <0.01 | |  |
| <11 weeks | 273 (31%) | -0.07 | 0.61 |  | |  |
| 11-35 weeks | 291 (33%) | -0.05 | 0.62 |  | |  |
| >35 weeks | 306 (35%) | 0.20 | 0.55 |  | |  |
|  |  |  |  |  | |  |
| Continuous covariates^a^ |  | General health score | | | |  |
|  |  | Correlation coefficient^c^ | | p-value^d^ | |  |
| Pre-pregnancy BMI |  | -0.10 | | <0.01 | |  |
| Maternal age at birth |  | 0.11 | | <0.01 | |  |
| Child age at follow up |  | -0.04 | | 0.21 | |  |
|  |  |  |  |  | |  |

For categorical covariates, the mean (sd) of the general health score in each category is presented in this table; for the continuous covariates, the correlation coefficient between this confounder and the general health score is showed.
Population: Helix study population (n=870)
Accronyms: BiB: Born in Bradford, EDEN: Étude des Déterminants pré et postnatals du développement et de la santé de l’Enfant, INMA: Infancia y Medio Ambiente, KANC: Kaunus Cohort, MoBa: The Norwegian Mother, Father and Child Cohort Study, RHEA: Mother-Child Cohort in Crete.
sd= standard deviation.
^a^ Imputed covariates (first imputed set)
^b^ Test of equality of mean between groups (one-way ANOVA)
^c^ Pearson coefficient of correlation.
^d^ Pearson correlation test (null hypothesis: correlation is equal to 0).

# eTable 16. Description of the general health score divided in terciles with health parameters and sub-scores

| **General health score ^a^** | **<T1**  **Bad general health** | **T1-T2**  **Basic general health** | **>T2**  **Good general health** |
| --- | --- | --- | --- |
| Cardiometabolic sub-score | -0,64 | -0,02 | 0,7 |
| Overweight ^a^ | 38% | 15% | 6% |
| Respiratory/allergy sub-score | -0,61 | 0,07 | 0,6 |
| Asthma | 23% | 8% | 5% |
| Mental sub-score | -0,65 | 0,05 | 0,74 |
| At risk for ADHD ^a^ | 15% | 9% | 2% |

The table gives the mean/percentage of some key health parameters and the three sub-scores in each tercile-based group of the general health score.
^a^ The general health score has been divided according to its terciles (Q33%= -0.22, Q66%=0.30)
^b^ Definition from the International Obesity Task Force (IOTF). Obese children are included as overweight. ^a^ ADHD score superior or equal than 17 (Conner’s rate).

# eTable 17. Complete results of the ExWAS on prenatal exposures

| **Exposure** | **Estimate (CI 95%)** | **p-value** | **p corrected** |
| --- | --- | --- | --- |
| Smoke (yes) | -0.06 [-0.17; 0.05] | 0,31 | 1,00 |
| Passive smoke (yes) | -0.13 [-0.23; -0.03] | 0,01 | 0,24 |
| Meat (medium) | 0.00 [-0.11; 0.11] | 0,98 | 1,00 |
| Meat (high) | -0.04 [-0.14; 0.07] | 0,51 | 1,00 |
| Fish (medium) | -0.01 [-0.12; 0.1] | 0,87 | 1,00 |
| Fish (high) | -0.01 [-0.13; 0.11] | 0,87 | 1,00 |
| Cereal (medium) | -0.01 [-0.14; 0.12] | 0,88 | 1,00 |
| Cereal (high) | -0.03 [-0.19; 0.13] | 0,69 | 1,00 |
| House crowding | 0.00 [-0.04; 0.03] | 0,88 | 1,00 |
| NO2 | 0.05 [-0.03; 0.13] | 0,22 | 1,00 |
| PM10 | 0.11 [-0.03; 0.25] | 0,11 | 1,00 |
| PM25 | 0.03 [-0.05; 0.12] | 0,41 | 1,00 |
| PM25abs | 0.05 [-0.07; 0.17] | 0,38 | 1,00 |
| Pressure | 0.04 [-0.15; 0.22] | 0,71 | 1,00 |
| Temperature | -0.04 [-0.19; 0.11] | 0,58 | 1,00 |
| Humidity | 0.00 [-0.25; 0.25] | 0,99 | 1,00 |
| NDVI | -0.06 [-0.18; 0.06] | 0,31 | 1,00 |
| Green spaces (yes) | 0.06 [-0.03; 0.15] | 0,20 | 1,00 |
| Blue spaces (yes) | 0.13 [-0.02; 0.28] | 0,09 | 1,00 |
| Population density | -0.01 [-0.06; 0.03] | 0,56 | 1,00 |
| Building density | 0.00 [-0.06; 0.05] | 0,89 | 1,00 |
| Connectivity | 0.03 [-0.05; 0.1] | 0,50 | 1,00 |
| Bus stop density | 0.04 [-0.04; 0.13] | 0,30 | 1,00 |
| Facility richness | 0.06 [-0.01; 0.12] | 0,08 | 1,00 |
| SEI | 0.04 [-0.01; 0.09] | 0,11 | 1,00 |
| Walkability | 0.07 [0; 0.14] | 0,06 | 1,00 |
| Traffic load | -0.02 [-0.1; 0.05] | 0,54 | 1,00 |
| Traffic nearest road | -0.01 [-0.06; 0.03] | 0,54 | 1,00 |
| Inverse distance to nearest road | 0.01 [-0.04; 0.06] | 0,65 | 1,00 |
| THM | 0.00 [-0.15; 0.15] | 0,99 | 1,00 |
| Brominated THM | -0.06 [-0.21; 0.09] | 0,46 | 1,00 |
| Chloroform | 0.01 [-0.16; 0.18] | 0,91 | 1,00 |
| BPA | 0.00 [-0.05; 0.06] | 0,96 | 1,00 |
| BUPA | 0.02 [-0.06; 0.1] | 0,64 | 1,00 |
| PRPA | 0.00 [-0.05; 0.05] | 0,92 | 1,00 |
| OXBE | -0.03 [-0.1; 0.04] | 0,40 | 1,00 |
| TRCS | -0.01 [-0.07; 0.06] | 0,87 | 1,00 |
| MBZP | -0.01 [-0.06; 0.05] | 0,78 | 1,00 |
| Molar sum of DEHP | -0.01 [-0.06; 0.04] | 0,67 | 1,00 |
| Molar sum of DINP | 0.00 [-0.06; 0.06] | 0,95 | 1,00 |
| MEP | -0.02 [-0.07; 0.03] | 0,41 | 1,00 |
| MIBP | -0.02 [-0.06; 0.03] | 0,42 | 1,00 |
| MNBP | -0.01 [-0.06; 0.04] | 0,58 | 1,00 |
| Hg | -0.01 [-0.09; 0.06] | 0,72 | 1,00 |
| PFHXS | 0.00 [-0.06; 0.06] | 0,88 | 1,00 |
| PFNA | -0.02 [-0.09; 0.04] | 0,51 | 1,00 |
| PFOA | -0.04 [-0.1; 0.03] | 0,25 | 1,00 |
| PFOS | 0.00 [-0.06; 0.06] | 0,93 | 1,00 |
| PFUNDA | 0.01 [-0.06; 0.08] | 0,74 | 1,00 |
| Cotinine (passive-smoke) | -0.06 [-0.19; 0.06] | 0,34 | 1,00 |
| Cotinine (smokers) | -0.13 [-0.24; -0.01] | 0,03 | 0,95 |
| DEP | 0.01 [-0.04; 0.06] | 0,77 | 1,00 |
| DMP | -0.01 [-0.06; 0.04] | 0,67 | 1,00 |
| DMTP | -0.01 [-0.07; 0.05] | 0,74 | 1,00 |
| DDE | -0.03 [-0.1; 0.04] | 0,46 | 1,00 |
| HCB | -0.02 [-0.07; 0.04] | 0,56 | 1,00 |
| SumPCB | -0.01 [-0.11; 0.09] | 0,83 | 1,00 |

# eTable 18. Complete results of the ExWAS on postnatal exposures

| **Exposure** | **Estimate (CI 95%)** | **p-value** | **p corrected** |
| --- | --- | --- | --- |
| Parental smoking (one) | -0.04 [-0.14; 0.05] | 0,36 | 1,00 |
| Parental smoking (both) | -0.04 [-0.17; 0.09] | 0,57 | 1,00 |
| ETS (exposed) | -0.12 [-0.2; -0.03] | 0,01 | 0,45 |
| Sweets (medium) | 0.02 [-0.07; 0.12] | 0,60 | 1,00 |
| Sweets (high) | -0.02 [-0.12; 0.08] | 0,68 | 1,00 |
| Meat (medium) | -0.03 [-0.13; 0.06] | 0,53 | 1,00 |
| Meat (high) | -0.05 [-0.14; 0.04] | 0,27 | 1,00 |
| Processed meat (medium) | 0.07 [-0.02; 0.16] | 0,12 | 1,00 |
| Processed meat (high) | 0.02 [-0.09; 0.12] | 0,74 | 1,00 |
| Fish (medium) | 0.06 [-0.04; 0.16] | 0,23 | 1,00 |
| Fish (high) | 0.01 [-0.1; 0.11] | 0,89 | 1,00 |
| Yogurt (medium) | 0.02 [-0.08; 0.12] | 0,75 | 1,00 |
| Yogurt (high) | 0.02 [-0.07; 0.12] | 0,63 | 1,00 |
| Beverages (medium) | 0.04 [-0.05; 0.13] | 0,36 | 1,00 |
| Beverages (high) | -0.08 [-0.18; 0.02] | 0,12 | 1,00 |
| Bread (medium) | -0.04 [-0.13; 0.05] | 0,34 | 1,00 |
| Bread (high) | 0.04 [-0.07; 0.14] | 0,49 | 1,00 |
| Breakfast cereals (medium) | 0.06 [-0.03; 0.15] | 0,22 | 1,00 |
| Breakfast cereals (high) | 0.09 [-0.01; 0.19] | 0,06 | 1,00 |
| Potatoes (medium) | -0.04 [-0.12; 0.05] | 0,40 | 1,00 |
| Potatoes (high) | -0.07 [-0.18; 0.04] | 0,21 | 1,00 |
| Vegetables (medium) | 0.02 [-0.09; 0.12] | 0,75 | 1,00 |
| Vegetables (high) | 0.11 [0.02; 0.2] | 0,02 | 1,00 |
| Dairy (medium) | 0.02 [-0.08; 0.11] | 0,74 | 1,00 |
| Dairy (high) | 0.01 [-0.09; 0.11] | 0,90 | 1,00 |
| Total fruits (medium) | 0 [-0.09; 0.1] | 0,96 | 1,00 |
| Total fruits (high) | -0.01 [-0.11; 0.09] | 0,81 | 1,00 |
| Cereal (medium) | 0.01 [-0.09; 0.1] | 0,89 | 1,00 |
| Cereal (high) | 0.01 [-0.08; 0.11] | 0,76 | 1,00 |
| Bakery (medium) | 0.07 [-0.03; 0.16] | 0,18 | 1,00 |
| Bakery (high) | -0.01 [-0.13; 0.1] | 0,79 | 1,00 |
| Total lipids (medium) | -0.03 [-0.14; 0.07] | 0,55 | 1,00 |
| Total lipids (high) | 0.03 [-0.08; 0.14] | 0,62 | 1,00 |
| Caffeinated drink (medium) | 0.01 [-0.1; 0.12] | 0,89 | 1,00 |
| Caffeinated drink (high) | -0.09 [-0.19; 0] | 0,05 | 1,00 |
| Fastfood (medium) | 0.01 [-0.08; 0.09] | 0,88 | 1,00 |
| Fastfood (high) | -0.14 [-0.28; -0.01] | 0,04 | 1,00 |
| Organic food (medium) | 0.03 [-0.07; 0.13] | 0,62 | 1,00 |
| Organic food (high) | 0.04 [-0.07; 0.15] | 0,46 | 1,00 |
| Ready-made (medium) | -0.01 [-0.1; 0.09] | 0,86 | 1,00 |
| Ready-made (high) | -0.02 [-0.12; 0.07] | 0,62 | 1,00 |
| KIDMED | 0.04 [0; 0.09] | 0,08 | 1,00 |
| sleep | 0.02 [-0.04; 0.08] | 0,42 | 1,00 |
| pet (Yes) | 0.15 [0.07; 0.23] | <0,01 | 0,03 |
| FAS (Middle) | 0.01 [-0.14; 0.15] | 0,94 | 1,00 |
| FAS (High) | 0.06 [-0.08; 0.21] | 0,40 | 1,00 |
| Participation (1 organisation) | 0.03 [-0.06; 0.12] | 0,48 | 1,00 |
| Participation (2 or more organisations) | 0.08 [-0.04; 0.2] | 0,19 | 1,00 |
| Contact family (Once a week) | -0.08 [-0.16; 0.01] | 0,08 | 1,00 |
| Contact family ((almost) Daily) | 0.03 [-0.14; 0.21] | 0,72 | 1,00 |
| Physical activity | 0.07 [0.01; 0.14] | 0,03 | 1,00 |
| Sedentary activity | 0 [-0.04; 0.04] | 0,97 | 1,00 |
| NO2 - home | -0.08 [-0.2; 0.05] | 0,23 | 1,00 |
| PM10 - home | -0.05 [-0.19; 0.09] | 0,47 | 1,00 |
| PM2.5 - home | -0.05 [-0.12; 0.03] | 0,21 | 1,00 |
| PM2.5abs - home | -0.04 [-0.11; 0.03] | 0,28 | 1,00 |
| NO2 - school | -0.08 [-0.2; 0.04] | 0,21 | 1,00 |
| PM 2.5 - school | -0.07 [-0.15; 0.01] | 0,10 | 1,00 |
| PM 2.5 abs - school | -0.08 [-0.17; 0.02] | 0,12 | 1,00 |
| Temperature | -0.01 [-0.08; 0.06] | 0,83 | 1,00 |
| Humidity | 0.05 [-0.03; 0.13] | 0,23 | 1,00 |
| UV | -0.01 [-0.08; 0.06] | 0,85 | 1,00 |
| NDVI - home | 0.02 [-0.07; 0.11] | 0,63 | 1,00 |
| NDVI - school | 0.01 [-0.07; 0.1] | 0,74 | 1,00 |
| Greenspace - home (yes) | 0.02 [-0.07; 0.12] | 0,65 | 1,00 |
| Greenspace - school (yes) | 0 [-0.09; 0.1] | 0,98 | 1,00 |
| Bluespace - home (yes) | 0.08 [-0.07; 0.23] | 0,32 | 1,00 |
| Bluespace - school (yes) | 0.1 [-0.05; 0.26] | 0,19 | 1,00 |
| Population density - home | -0.03 [-0.09; 0.02] | 0,24 | 1,00 |
| Population density - school | -0.01 [-0.06; 0.05] | 0,78 | 1,00 |
| Built density - home | -0.03 [-0.09; 0.02] | 0,23 | 1,00 |
| Built density - school | -0.04 [-0.08; 0] | 0,08 | 1,00 |
| Connect density - home | 0.01 [-0.07; 0.08] | 0,90 | 1,00 |
| Connect density - school | -0.03 [-0.1; 0.04] | 0,36 | 1,00 |
| Bststop density - home | 0.03 [-0.11; 0.16] | 0,70 | 1,00 |
| Bststop density - school | 0 [-0.08; 0.08] | 0,95 | 1,00 |
| Facility richness - home | 0.02 [-0.04; 0.09] | 0,46 | 1,00 |
| Facility richness - school | -0.01 [-0.06; 0.05] | 0,84 | 1,00 |
| Facility density - home | 0 [-0.08; 0.07] | 0,95 | 1,00 |
| Facility density - school | -0.02 [-0.08; 0.03] | 0,41 | 1,00 |
| SEI - home | 0.04 [-0.02; 0.1] | 0,18 | 1,00 |
| SEI - school | 0.01 [-0.05; 0.08] | 0,68 | 1,00 |
| walkability - home | 0.02 [-0.06; 0.09] | 0,67 | 1,00 |
| walkability - school | -0.02 [-0.07; 0.04] | 0,57 | 1,00 |
| Traffic load - home | -0.06 [-0.13; 0.01] | 0,10 | 1,00 |
| Traffic nearest road - home | -0.04 [-0.12; 0.04] | 0,30 | 1,00 |
| Inverse distance to nearest road - home | 0 [-0.06; 0.05] | 0,91 | 1,00 |
| Traffic load – school | -0.02 [-0.1; 0.06] | 0,57 | 1,00 |
| Traffic nearest road - school | -0.06 [-0.13; 0.02] | 0,13 | 1,00 |
| Inverse distance to nearest road - school | -0.02 [-0.07; 0.04] | 0,54 | 1,00 |
| TEX indoor | -0.03 [-0.1; 0.04] | 0,41 | 1,00 |
| NO2 indoor | -0.11 [-0.2; -0.01] | 0,03 | 1,00 |
| Benzene indoor | -0.1 [-0.16; -0.05] | <0,01 | 0,02 |
| PM 2.5 indoor | -0.08 [-0.12; -0.04] | <0,01 | 0,01 |
| PM absorbance indoor | -0.06 [-0.1; -0.01] | 0,01 | 0,62 |
| BPA | 0.02 [-0.02; 0.06] | 0,42 | 1,00 |
| BUPA | 0 [-0.03; 0.04] | 0,80 | 1,00 |
| OXBE | 0 [-0.06; 0.05] | 0,86 | 1,00 |
| TRCS | -0.01 [-0.06; 0.03] | 0,60 | 1,00 |
| ETPA | -0.02 [-0.05; 0] | 0,10 | 1,00 |
| MEPA | -0.05 [-0.09; -0.01] | 0,03 | 1,00 |
| MBZP | 0 [-0.04; 0.04] | 0,99 | 1,00 |
| SumDEHP | -0.04 [-0.09; 0.02] | 0,19 | 1,00 |
| MEP | -0.01 [-0.07; 0.05] | 0,70 | 1,00 |
| MIBP | 0 [-0.07; 0.06] | 0,90 | 1,00 |
| MNBP | -0.02 [-0.08; 0.04] | 0,48 | 1,00 |
| SumDINP | -0.03 [-0.07; 0.01] | 0,20 | 1,00 |
| K | 0 [-0.06; 0.06] | 0,94 | 1,00 |
| Mg | 0 [-0.05; 0.04] | 0,91 | 1,00 |
| Na | 0.05 [0.01; 0.09] | 0,01 | 0,54 |
| Se | -0.12 [-0.17; -0.07] | <0,01 | <0,01 |
| Zn | 0.02 [-0.05; 0.09] | 0,61 | 1,00 |
| Cd | 0 [-0.06; 0.07] | 0,91 | 1,00 |
| Co | -0.04 [-0.09; 0.01] | 0,12 | 1,00 |
| Cu | 0.02 [-0.01; 0.05] | 0,19 | 1,00 |
| Cs | 0 [-0.05; 0.04] | 0,87 | 1,00 |
| Hg | -0.07 [-0.21; 0.07] | 0,32 | 1,00 |
| Mn | 0.07 [0; 0.14] | 0,06 | 1,00 |
| Mo | 0.06 [0.01; 0.11] | 0,02 | 0,94 |
| Pb | 0.1 [0.05; 0.16] | <0,01 | <0,01 |
| Tl (detected) | 0.09 [0.03; 0.15] | <0,01 | 0,11 |
| PFHXS | -0.01 [-0.04; 0.02] | 0,48 | 1,00 |
| PFNA | 0.02 [-0.04; 0.08] | 0,42 | 1,00 |
| PFOA | 0.15 [0.07; 0.23] | <0,01 | 0,03 |
| PFOS | 0.01 [-0.14; 0.15] | 0,94 | 1,00 |
| PBDE47 | 0.06 [-0.08; 0.21] | 0,40 | 1,00 |
| Cotinine (detected) | -0.05 [-0.16; 0.06] | 0,41 | 1,00 |
| DEP | 0.05 [0; 0.11] | 0,06 | 1,00 |
| DMTP | 0.01 [-0.04; 0.06] | 0,57 | 1,00 |
| DDE | 0.14 [0.08; 0.2] | <0,01 | <0,01 |
| HCB | 0.14 [0.08; 0.21] | <0,01 | <0,01 |
| sumPCB | 0.18 [0.1; 0.26] | <0,01 | <0,01 |

# eTable 19. Final multivariable models with and without fish consumption as a confounder (results for postnatal PFOA).

|  | Estimate [CI 95%] | p-value |
| --- | --- | --- |
| PFOA unadjusted on fish consumption^a^ | 0.08 [0.03; 0.13] | <0.01 |
| PFOA adjusted on total fish consumption^b^ | 0.08 [0.03; 0.13] | <0.01 |

a: These estimates correspond to the original multivariable model presented in Table 4 of the manuscript.
b: These estimates correspond to the same multivariable model to which total fish consumption was added a confounder variable.

# eFigure 1. Variable plot - Mixed Factorial Analysis on standardized variables of mental health (Helix subcohort, n=1278 with mental health data)


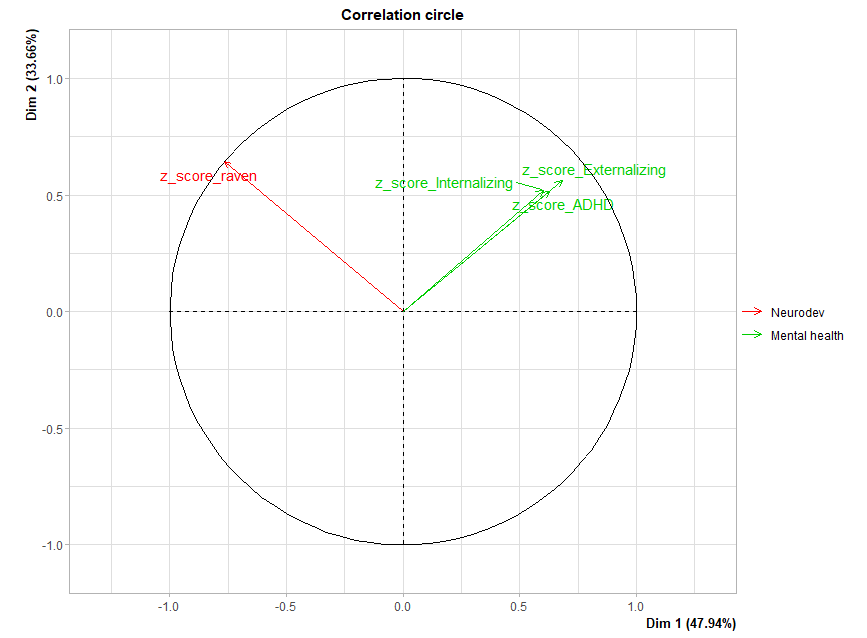


# eFigure 2. Variable plots - Mixed Factorial Analysis on standardized variables of respiratory health and allergies (Helix subcohort, n=1009 with respiratory and allergic data)

a) Qualitative variables

**
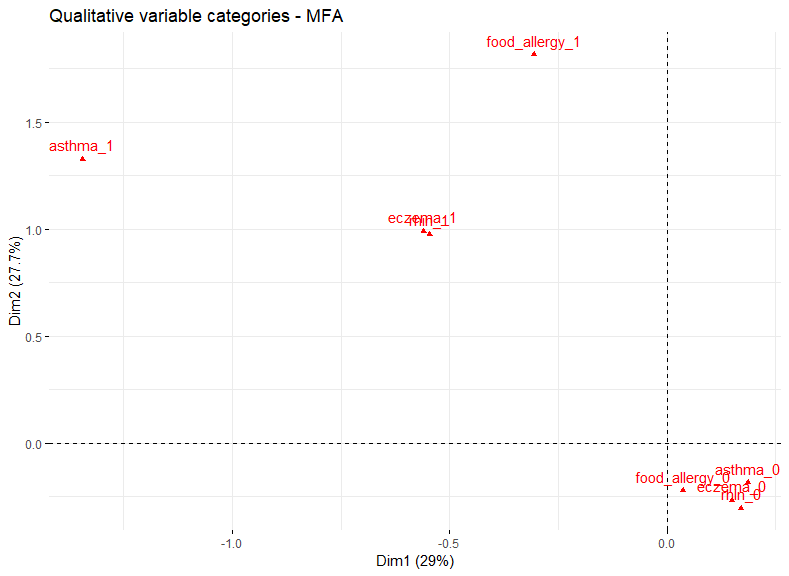
**

b) Quantitative variable

**
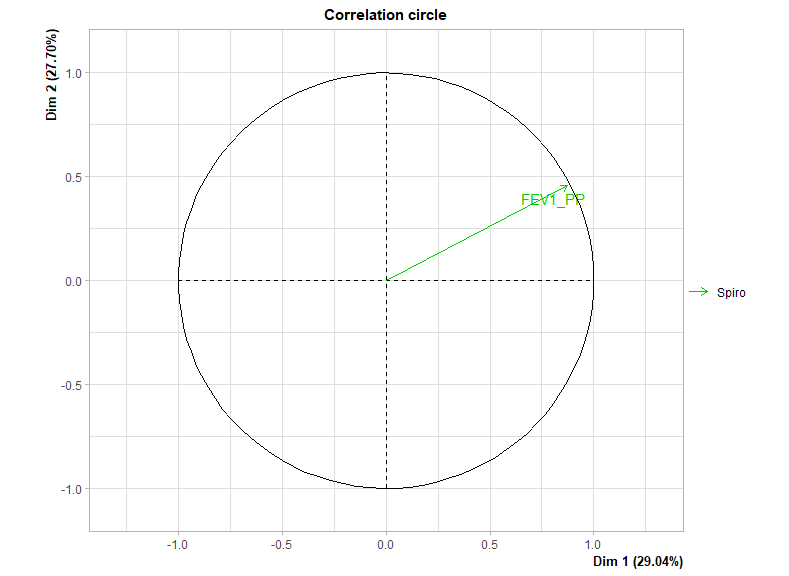
**

# eFigure 3. Distribution of each sub-score by cohort


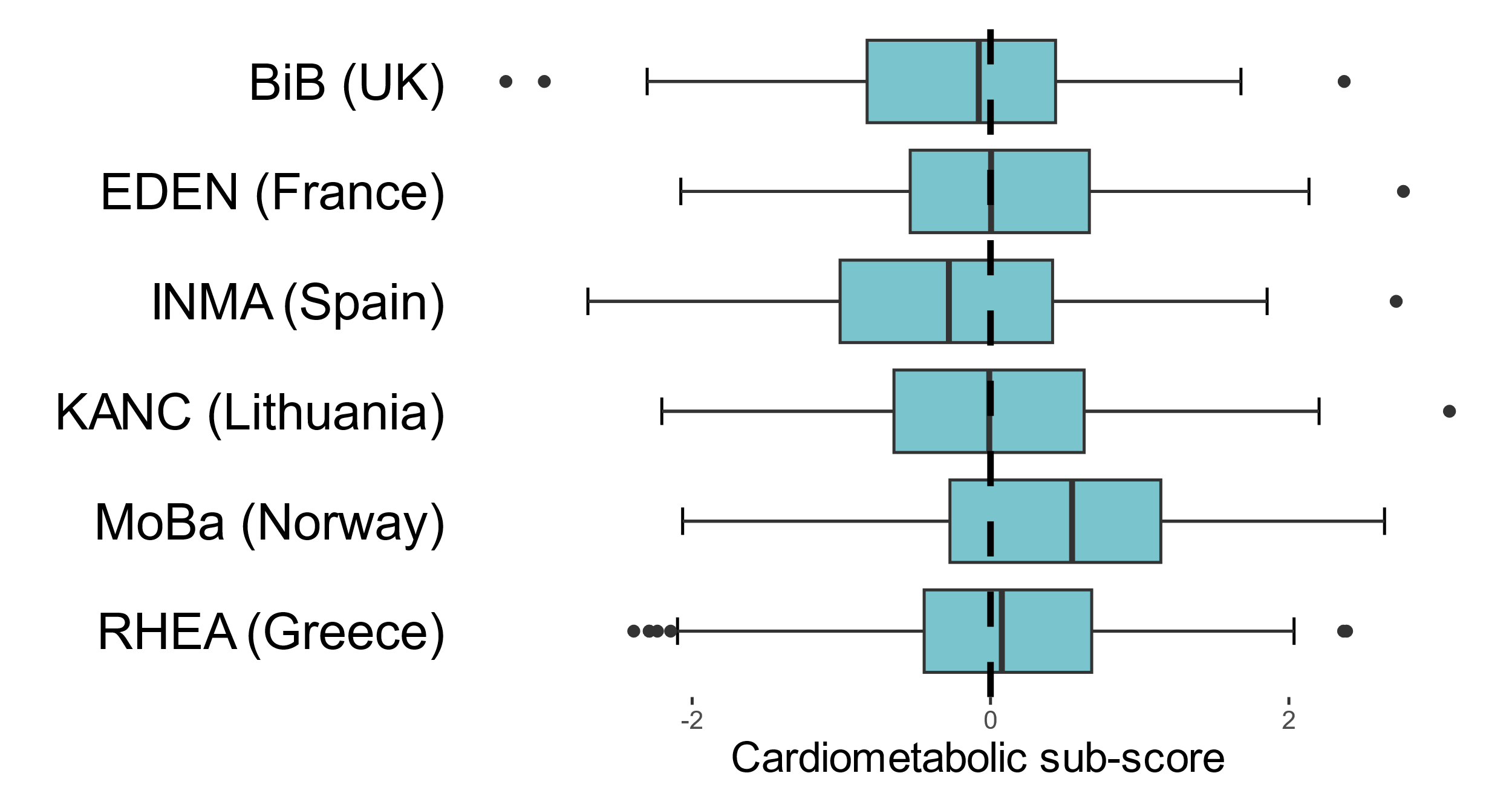


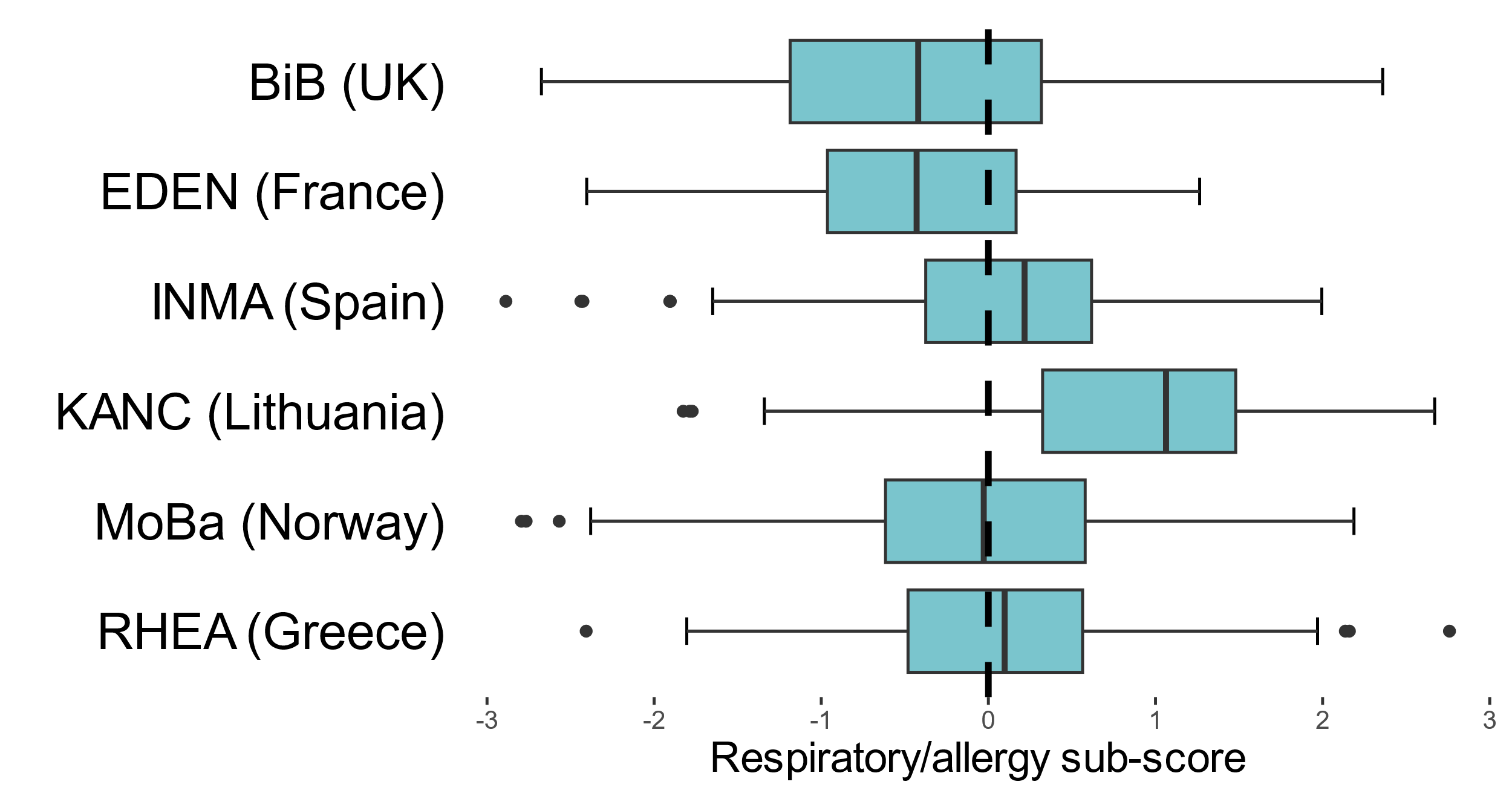


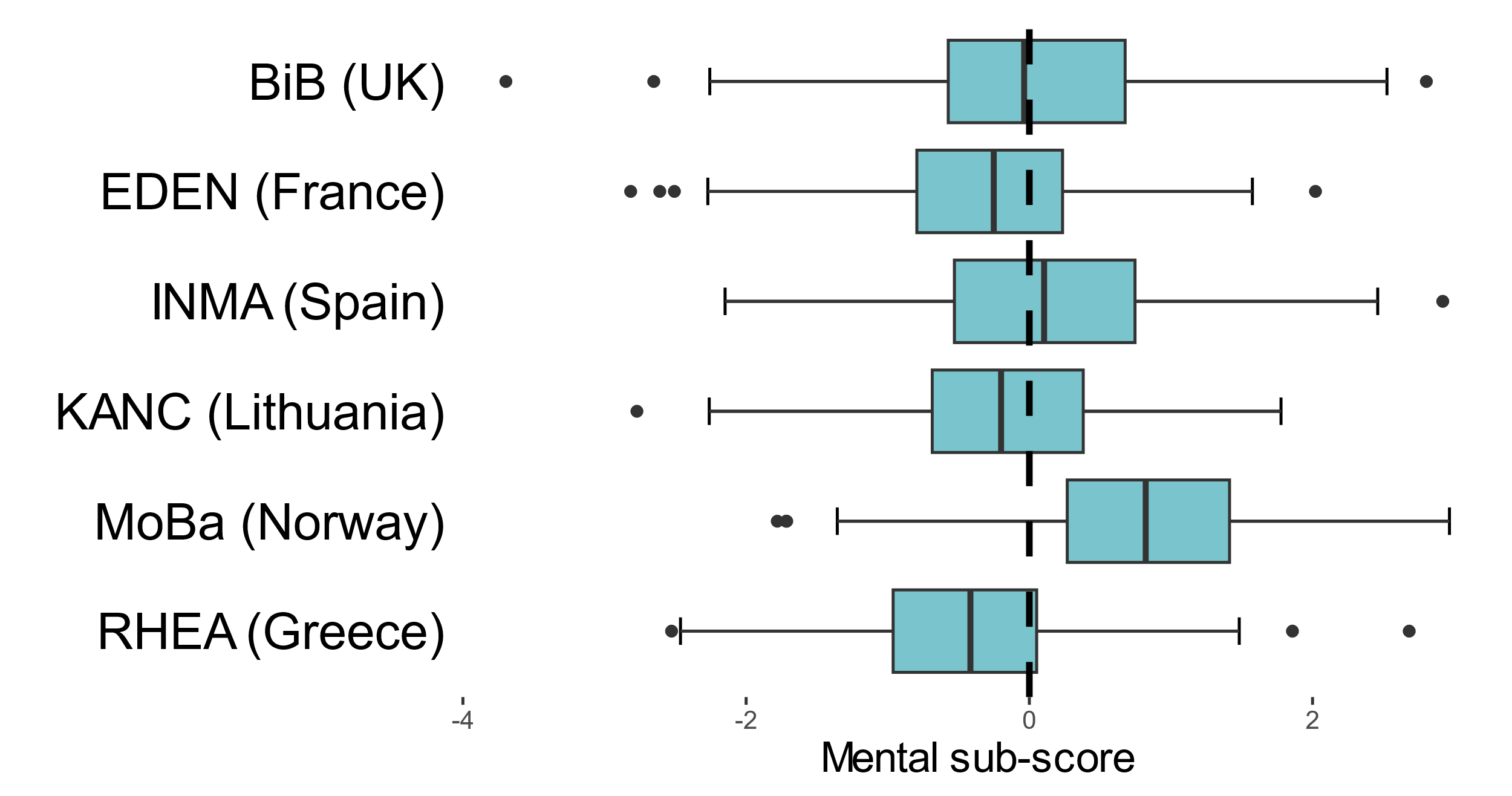


# eFigure 4. Multi-exposure model on postnatal exposures stratified on the terciles of z-BMI.

**
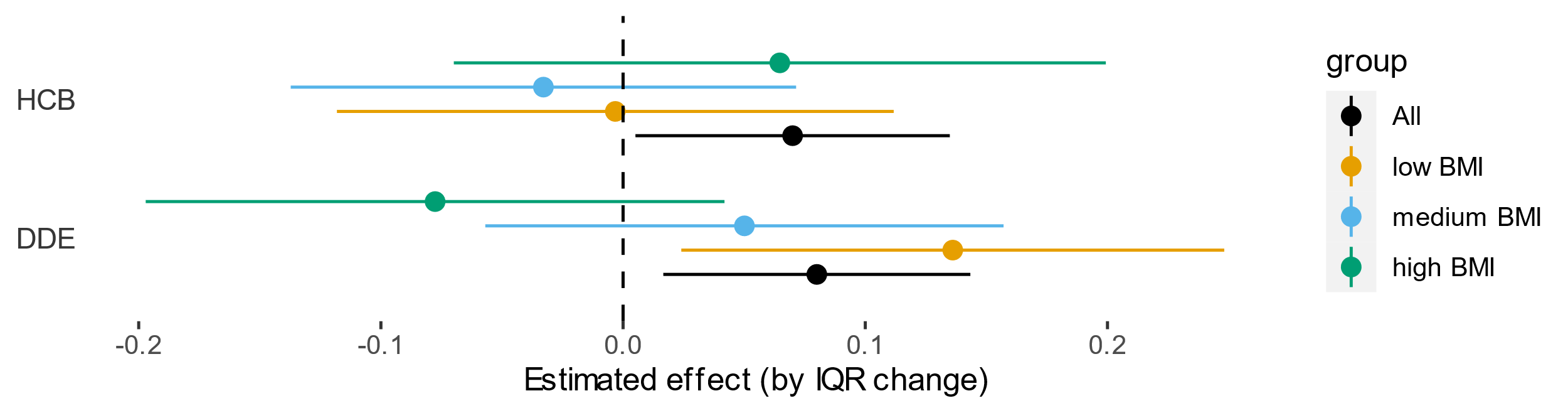
**

Note: only results on organochlorine’s compounds are shown here as the hypothesis is that these specific compounds accumulate in fat. The whole model included all selected postnatal exposures.

# eFigure 5. Multi-exposure model on prenatal exposures stratified on sex

**
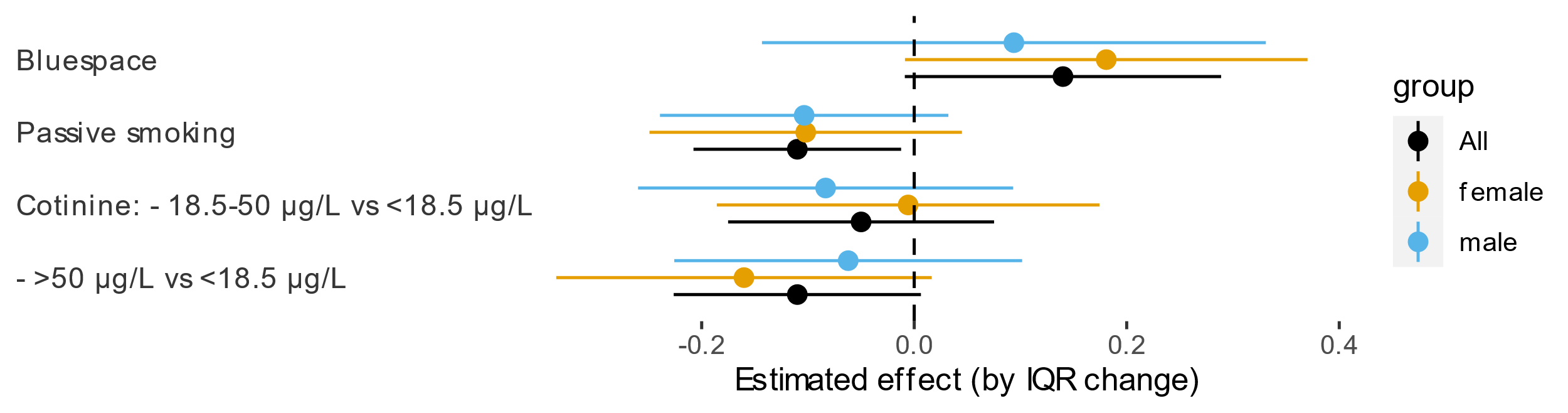
**

# eFigure 6. Multi-exposure model on postnatal exposures stratified on sex

**
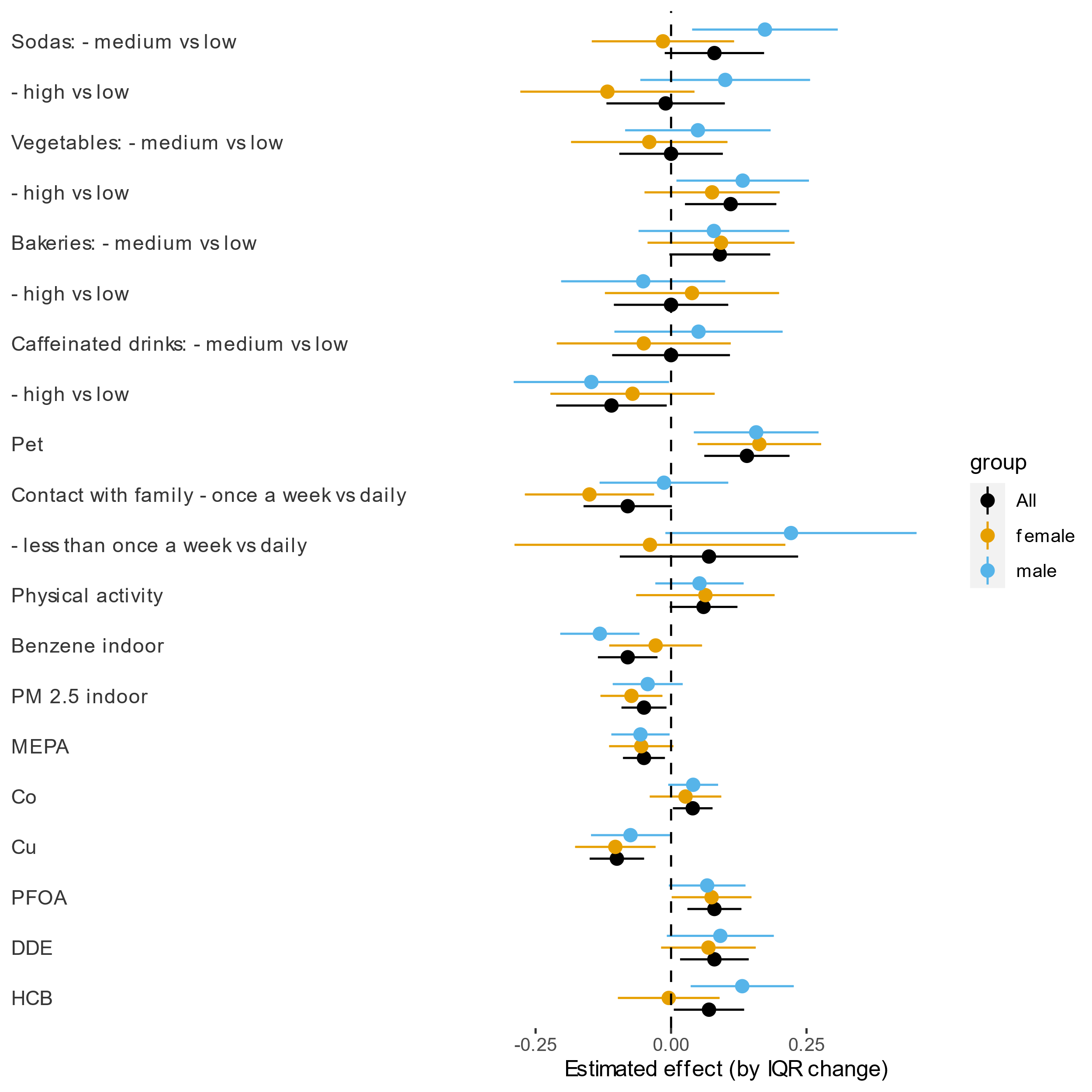
**

# eFigure 7. Multi-exposure model on postnatal exposures stratified on cohorts

**
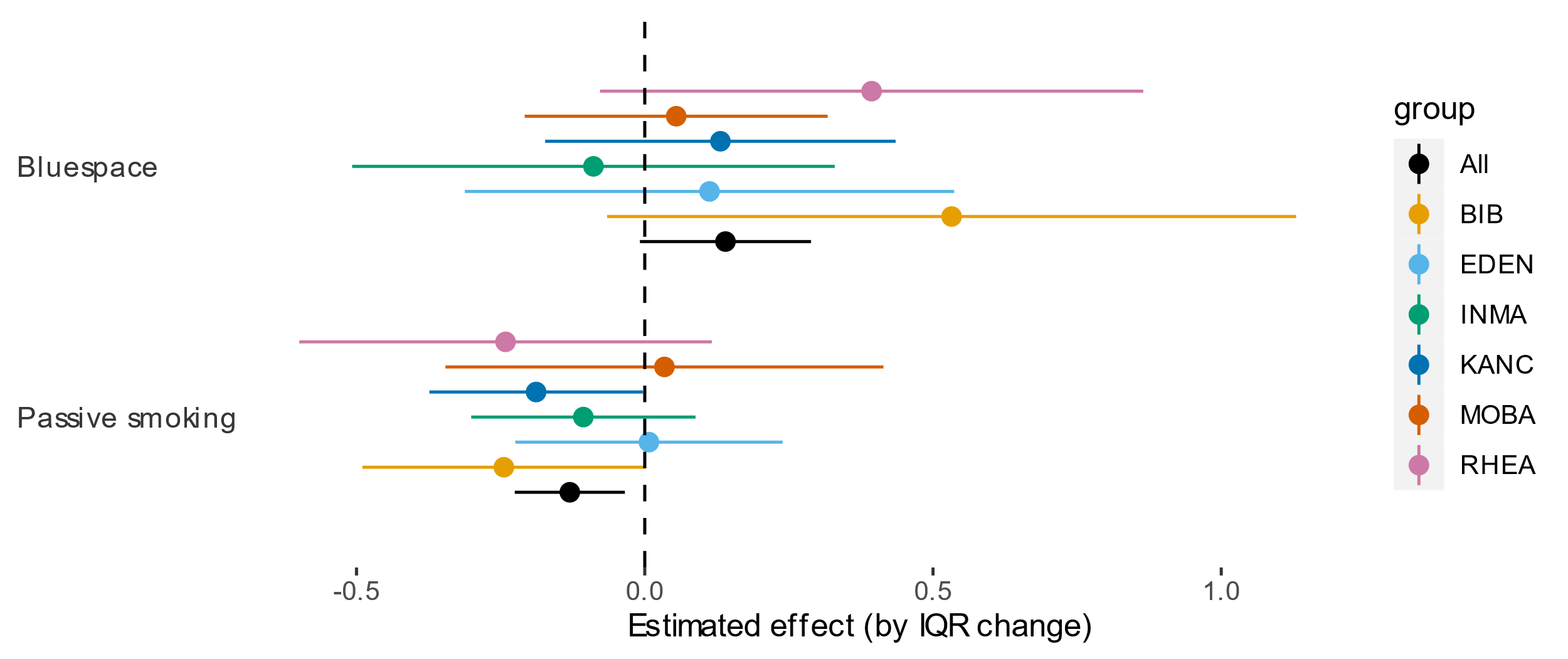
**

Note: cotinine was not included in this model because missing in the whole KANC cohort before imputation.

# eFigure 8. Multi-exposure model on postnatal exposures stratified on cohorts

**
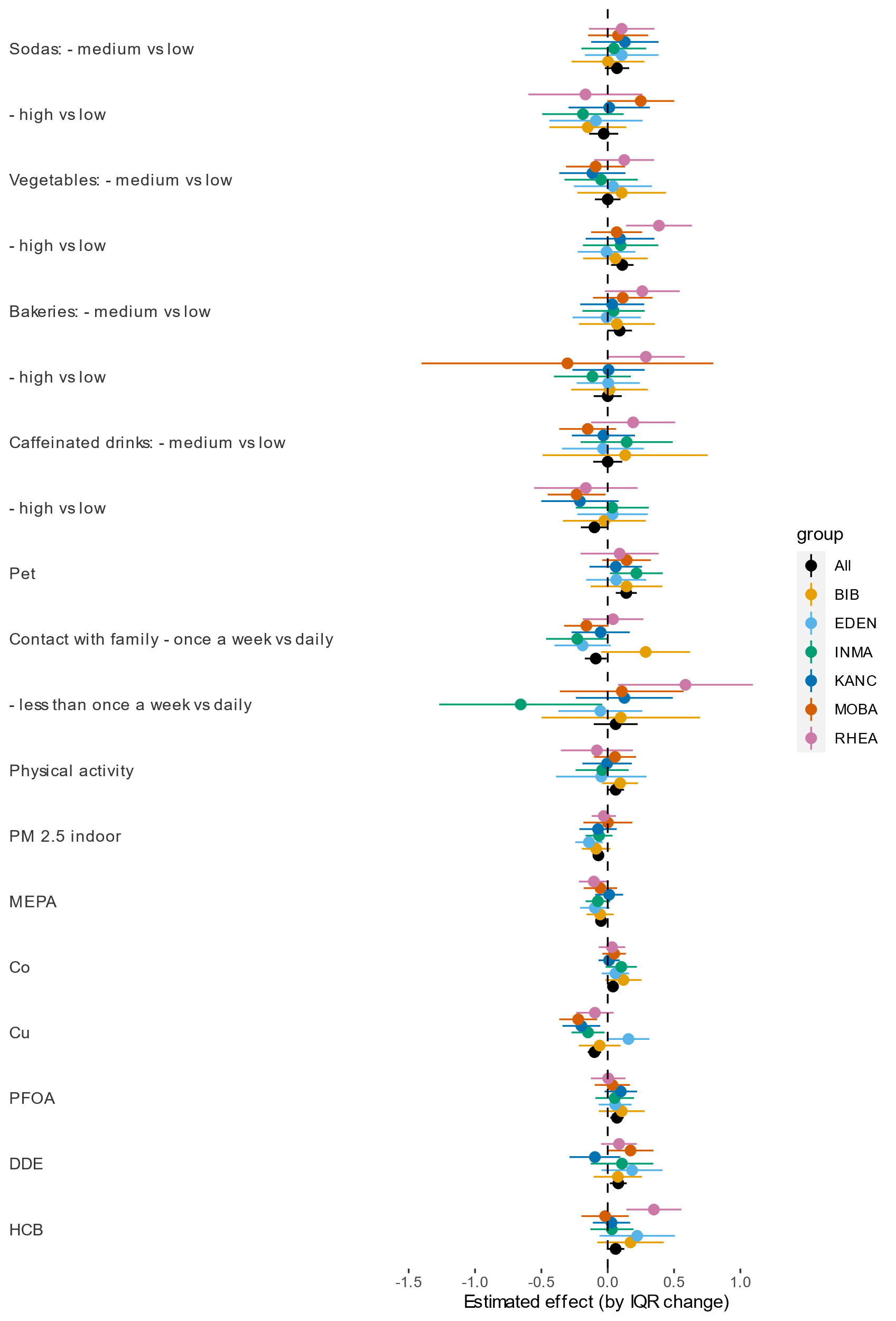
**

Note: bluespace at school was not included in this model because there were all children from RHEA had the same value before imputation (no access).

# eFigure 9. GAM estimated curve for postnatal selected exposures by LASSO


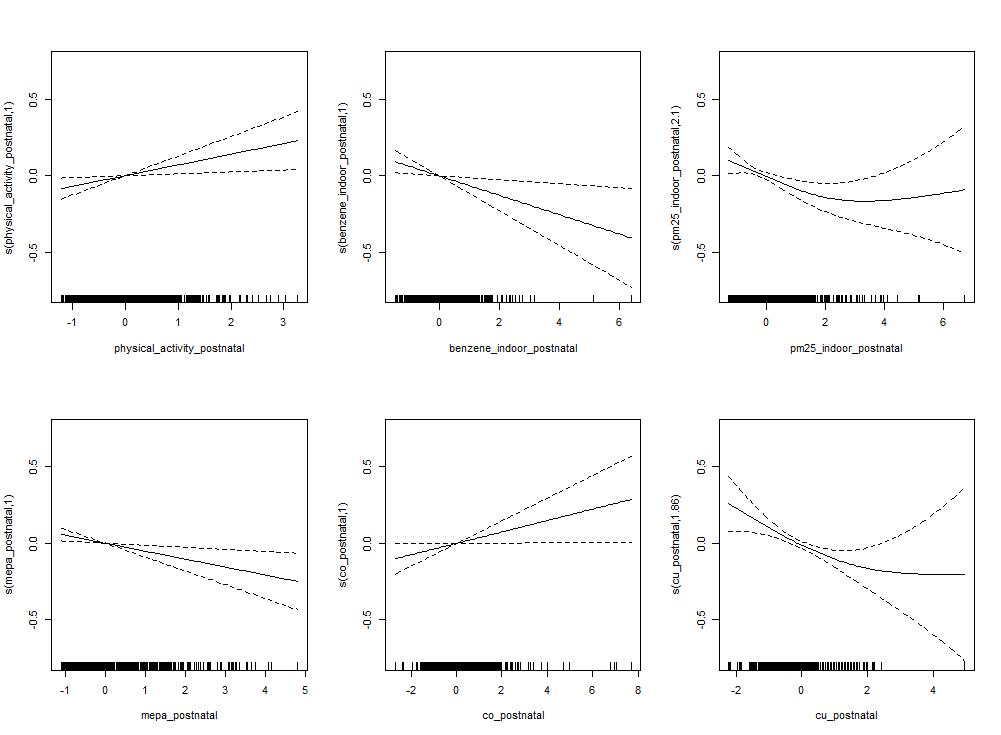


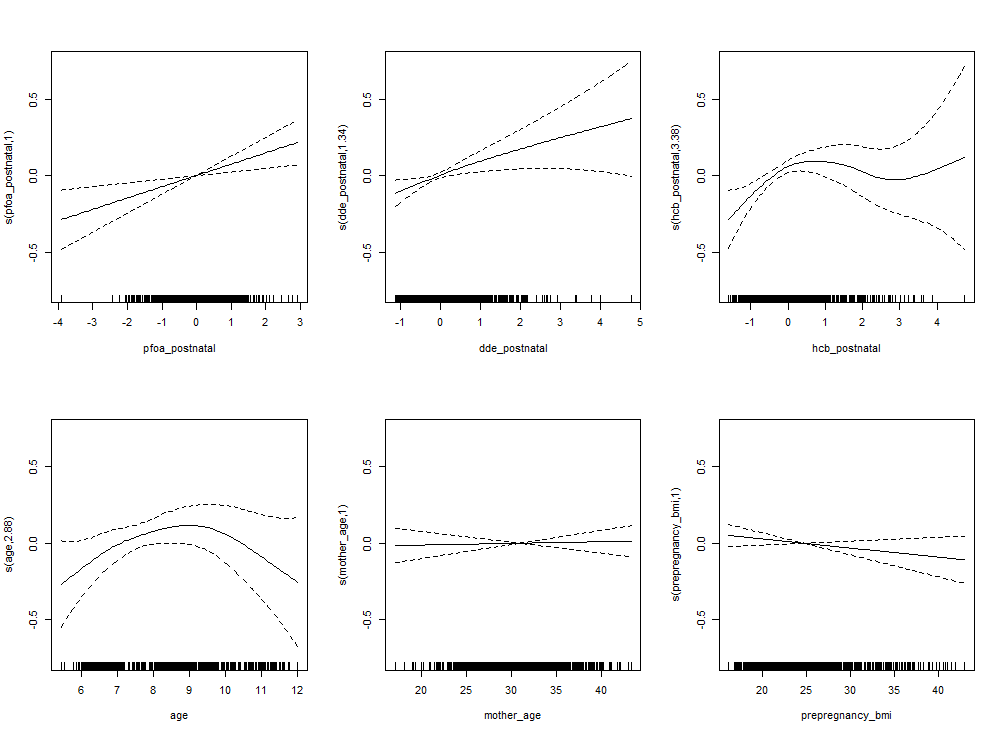


# eFigure 10. Multi-exposure model without the 4% most extreme values for the general health score

**
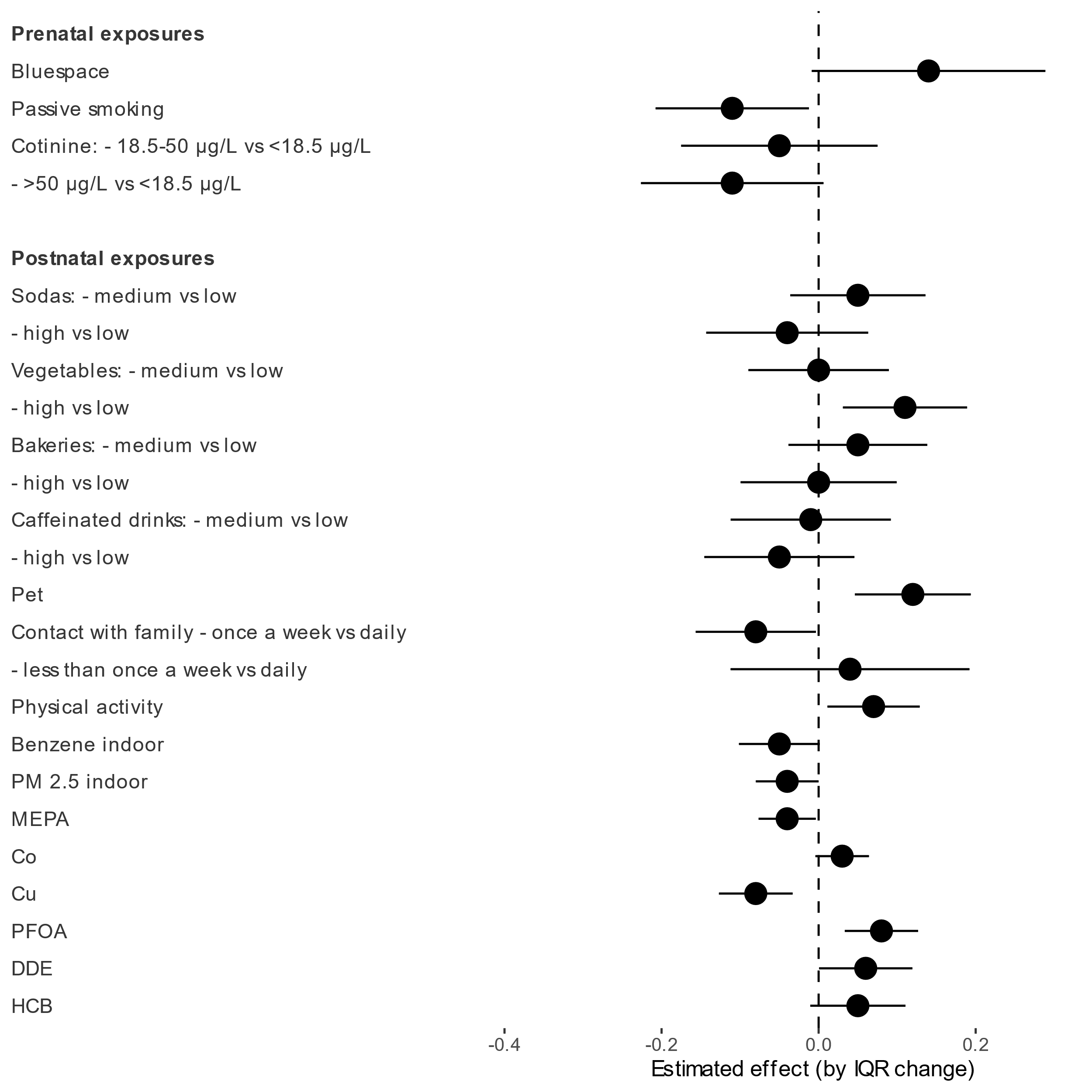
**

# eReferences

1. Warembourg C, Maitre L, Tamayo-Uria I, et al. Early-Life Environmental Exposures and Blood Pressure in Children. *J Am Coll Cardiol*. 2019;74(10):1317-1328. doi:10.1016/j.jacc.2019.06.069

2. Vrijheid M, Fossati S, Maitre L, et al. Early-Life Environmental Exposures and Childhood Obesity: An Exposome-Wide Approach. *Environ Health Perspect*. 2020;128(6):67009. doi:10.1289/EHP5975

3. de Onis M, Onyango AW, Borghi E, Siyam A, Nishida C, Siekmann J. Development of a WHO growth reference for school-aged children and adolescents. *Bull World Health Organ*. 2007;85(9):660-667. doi:10.2471/blt.07.043497

4. Horowitz G, Zaman Z, Blanckaert N, et al. MODULAR ANALYTICS: A new approach to automation in the clinical laboratory. *J Autom Methods Manag Chem*. 2005;2005:8-25. doi:10.1155/JAMMC.2005.8

5. Stratakis N, Conti DV, Borras E, et al. Association of Fish Consumption and Mercury Exposure During Pregnancy With Metabolic Health and Inflammatory Biomarkers in Children. *JAMA Netw Open*. 2020;3(3):e201007. doi:10.1001/jamanetworkopen.2020.1007

6. Agier L, Basagaña X, Maitre L, et al. Early-life exposome and lung function in children in Europe: an analysis of data from the longitudinal, population-based HELIX cohort. *Lancet Planet Health*. 2019;3(2):e81-e92. doi:10.1016/S2542-5196(19)30010-5

7. Granum B, Oftedal B, Agier L, et al. Multiple environmental exposures in early-life and allergy-related outcomes in childhood. *Environ Int*. 2020;144:106038. doi:10.1016/j.envint.2020.106038

8. Asher MI, Keil U, Anderson HR, et al. International Study of Asthma and Allergies in Childhood (ISAAC): rationale and methods. *Eur Respir J*. 1995;8(3):483-491. doi:10.1183/09031936.95.08030483

9. Raven JC, Raven J. Progressive matrices couleur/colored progressive matrices. *Paris Cent Psychol Appliquée*. Published online 1998.

10. Achenbach T, Rescorla L. *Manual for the ASEBA School-Age Forms and Profiles. An Integrated System of Multi-Informant Assessment.*; 2001.

11. Gurley JR. Conners’ Parent Rating Scales – Revised. In: Goldstein S, Naglieri JA, eds. *Encyclopedia of Child Behavior and Development*. Springer US; 2011:404-405. doi:10.1007/978-0-387-79061-9_670

12. Maitre L, Julvez J, López-Vicente M, et al. Early-life environmental exposure determinants of child behavior in Europe: A longitudinal, population-based study. *Environ Int*. 2021;153:106523. doi:10.1016/j.envint.2021.106523

13. Beelen R, Hoek G, Pebesma E, Vienneau D, de Hoogh K, Briggs DJ. Mapping of background air pollution at a fine spatial scale across the European Union. *Sci Total Environ*. 2009;407(6):1852-1867. doi:10.1016/j.scitotenv.2008.11.048

14. Cyrys J, Eeftens M, Heinrich J, et al. Variation of NO2 and NOx concentrations between and within 36 European study areas: Results from the ESCAPE study. *Atmos Environ*. 2012;62:374-390. doi:10.1016/j.atmosenv.2012.07.080

15. Eeftens M, Tsai MY, Ampe C, et al. Spatial variation of PM2.5, PM10, PM2.5 absorbance and PMcoarse concentrations between and within 20 European study areas and the relationship with NO2 – Results of the ESCAPE project. *Atmos Environ*. 2012;62:303-317. doi:10.1016/j.atmosenv.2012.08.038

16. Beelen R, Hoek G, Vienneau D, et al. Development of NO2 and NOx land use regression models for estimating air pollution exposure in 36 study areas in Europe – The ESCAPE project. *Atmos Environ*. 2013;72:10-23. doi:10.1016/j.atmosenv.2013.02.037

17. Eeftens M, Beelen R, de Hoogh K, et al. Development of Land Use Regression Models for PM2.5, PM2.5 Absorbance, PM10 and PMcoarse in 20 European Study Areas; Results of the ESCAPE Project. *Environ Sci Technol*. 2012;46(20):11195-11205. doi:10.1021/es301948k

18. Schembari A, de HK, Pedersen M, et al. Ambient Air Pollution and Newborn Size and Adiposity at Birth: Differences by Maternal Ethnicity (the Born in Bradford Study Cohort). *Environ Health Perspect*. 2015;123(11):1208-1215. doi:10.1289/ehp.1408675

19. Wang M, Beelen R, Bellander T, et al. Performance of Multi-City Land Use Regression Models for Nitrogen Dioxide and Fine Particles. *Environ Health Perspect*. 2014;122(8):843-849. doi:10.1289/ehp.1307271

20. Rahmalia A, Giorgis-Allemand L, Lepeule J, et al. Pregnancy exposure to atmospheric pollutants and placental weight: an approach relying on a dispersion model. *Environ Int*. 2012;48:47-55. doi:10.1016/j.envint.2012.06.013

21. Nieuwenhuijsen MJ, Kruize H, Gidlow C, et al. Positive health effects of the natural outdoor environment in typical populations in different regions in Europe (PHENOTYPE): a study programme protocol. *BMJ Open*. 2014;4(4):e004951. doi:10.1136/bmjopen-2014-004951

22. Weier, J. and Herring, D. (2000) Measuring Vegetation (NDVI & EVI). NASA Earth Observatory, Washington DC. - References - Scientific Research Publishing. Accessed February 3, 2023. https://scirp.org/reference/referencespapers.aspx?referenceid=2089851

23. Urban Atlas — European Environment Agency. Accessed February 3, 2023. https://www.eea.europa.eu/data-and-maps/data/urban-atlas

24. Smargiassi A, Goldberg MS, Plante C, Fournier M, Baudouin Y, Kosatsky T. Variation of daily warm season mortality as a function of micro-urban heat islands. *J Epidemiol Community Health*. 2009;63(8):659-664. doi:10.1136/jech.2008.078147

25. Duncan DT, Aldstadt J, Whalen J, Melly SJ, Gortmaker SL. Validation of Walk Score® for Estimating Neighborhood Walkability: An Analysis of Four US Metropolitan Areas. *Int J Environ Res Public Health*. 2011;8(11):4160-4179. doi:10.3390/ijerph8114160

26. Frank LD, Sallis JF, Conway TL, Chapman JE, Saelens BE, Bachman W. Many Pathways from Land Use to Health: Associations between Neighborhood Walkability and Active Transportation, Body Mass Index, and Air Quality. *J Am Plann Assoc*. 2006;72(1):75-87. doi:10.1080/01944360608976725

27. Walk Score Terms of Use. Accessed February 3, 2023. https://www.walkscore.com/terms-of-use.shtml

28. OpenStreetMap. OpenStreetMap. Accessed February 3, 2023. https://www.openstreetmap.org/

29. van Nunen E, Vermeulen R, Tsai MY, et al. Land Use Regression Models for Ultrafine Particles in Six European Areas. *Environ Sci Technol*. 2017;51(6):3336-3345. doi:10.1021/acs.est.6b05920

30. Jeong CH, Wagner ED, Siebert VR, et al. Occurrence and Toxicity of Disinfection Byproducts in European Drinking Waters in Relation with the HIWATE Epidemiology Study. *Environ Sci Technol*. 2012;46(21):12120-12128. doi:10.1021/es3024226

31. Smith RB, Edwards SC, Best N, Wright J, Nieuwenhuijsen MJ, Toledano MB. Birth Weight, Ethnicity, and Exposure to Trihalomethanes and Haloacetic Acids in Drinking Water during Pregnancy in the Born in Bradford Cohort. *Environ Health Perspect*. 2016;124(5):681-689. doi:10.1289/ehp.1409480

32. Villanueva CM, Gracia-Lavedán E, Ibarluzea J, et al. Exposure to trihalomethanes through different water uses and birth weight, small for gestational age, and preterm delivery in Spain. *Environ Health Perspect*. 2011;119(12):1824-1830. doi:10.1289/ehp.1002425

33. Stayner LT, Pedersen M, Patelarou E, et al. Exposure to Brominated Trihalomethanes in Water During Pregnancy and Micronuclei Frequency in Maternal and Cord Blood Lymphocytes. *Environ Health Perspect*. 2014;122(1):100-106. doi:10.1289/ehp.1206434

34. Danileviciute A, Grazuleviciene R, Vencloviene J, Paulauskas A, Nieuwenhuijsen MJ. Exposure to Drinking Water Trihalomethanes and Their Association with Low Birth Weight and Small for Gestational Age in Genetically Susceptible Women. *Int J Environ Res Public Health*. 2012;9(12):4470-4485. doi:10.3390/ijerph9124470

35. Maitre L, de Bont J, Casas M, et al. Human Early Life Exposome (HELIX) study: a European population-based exposome cohort. *BMJ Open*. 2018;8(9):e021311. doi:10.1136/bmjopen-2017-021311

36. Serra-Majem L, Ribas L, Ngo J, et al. Food, youth and the Mediterranean diet in Spain. Development of KIDMED, Mediterranean Diet Quality Index in children and adolescents. *Public Health Nutr*. 2004;7(7):931-935. doi:10.1079/phn2004556

37. Liu Y, Wang M, Villberg J, et al. Reliability and Validity of Family Affluence Scale (FAS II) among Adolescents in Beijing, China. *Child Indic Res*. 2012;5(2):235-251. doi:10.1007/s12187-011-9131-5

38. Boyce W, Torsheim T, Currie C, Zambon A. The Family Affluence Scale as a Measure of National Wealth: Validation of an Adolescent Self-Report Measure. *Soc Indic Res*. 2006;78(3):473-487. doi:10.1007/s11205-005-1607-6

39. Haug LS, Sakhi AK, Cequier E, et al. In-utero and childhood chemical exposome in six European mother-child cohorts. *Environ Int*. 2018;121(Pt 1):751-763. doi:10.1016/j.envint.2018.09.056

40. Caspersen IH, Kvalem HE, Haugen M, et al. Determinants of plasma PCB, brominated flame retardants, and organochlorine pesticides in pregnant women and 3 year old children in The Norwegian Mother and Child Cohort Study. *Environ Res*. 2016;146:136-144. doi:10.1016/j.envres.2015.12.020

41. Goñi F, López R, Etxeandia A, Millán E, Amiano P. High throughput method for the determination of organochlorine pesticides and polychlorinated biphenyls in human serum. *J Chromatogr B Analyt Technol Biomed Life Sci*. 2007;852(1-2):15-21. doi:10.1016/j.jchromb.2006.12.049

42. Koponen J, Rantakokko P, Airaksinen R, Kiviranta H. Determination of selected perfluorinated alkyl acids and persistent organic pollutants from a small volume human serum sample relevant for epidemiological studies. *J Chromatogr A*. 2013;1309:48-55. doi:10.1016/j.chroma.2013.07.064

43. Haug LS, Thomsen C, Becher G. A sensitive method for determination of a broad range of perfluorinated compounds in serum suitable for large-scale human biomonitoring. *J Chromatogr A*. 2009;1216(3):385-393. doi:10.1016/j.chroma.2008.10.113

44. Poothong S, Lundanes E, Thomsen C, Haug LS. High throughput online solid phase extraction-ultra high performance liquid chromatography-tandem mass spectrometry method for polyfluoroalkyl phosphate esters, perfluoroalkyl phosphonates, and other perfluoroalkyl substances in human serum, plasma, and whole blood. *Anal Chim Acta*. 2017;957:10-19. doi:10.1016/j.aca.2016.12.043

45. Manzano-Salgado CB, Casas M, Lopez-Espinosa MJ, et al. Transfer of perfluoroalkyl substances from mother to fetus in a Spanish birth cohort. *Environ Res*. 2015;142:471-478. doi:10.1016/j.envres.2015.07.020

46. Poothong S, Thomsen C, Padilla-Sanchez JA, Papadopoulou E, Haug LS. Distribution of Novel and Well-Known Poly- and Perfluoroalkyl Substances (PFASs) in Human Serum, Plasma, and Whole Blood. *Environ Sci Technol*. 2017;51(22):13388-13396. doi:10.1021/acs.est.7b03299

47. Rodushkin I, Axelsson MD. Application of double focusing sector field ICP-MS for multielemental characterization of human hair and nails. Part II. A study of the inhabitants of northern Sweden. *Sci Total Environ*. 2000;262(1-2):21-36. doi:10.1016/s0048-9697(00)00531-3

48. Ramon R, Murcia M, Aguinagalde X, et al. Prenatal mercury exposure in a multicenter cohort study in Spain. *Environ Int*. 2011;37(3):597-604. doi:10.1016/j.envint.2010.12.004

49. Stern AH, Smith AE. An assessment of the cord blood:maternal blood methylmercury ratio: implications for risk assessment. *Environ Health Perspect*. 2003;111(12):1465-1470.

50. Padilla MA, Elobeid M, Ruden DM, Allison DB. An Examination of the Association of Selected Toxic Metals with Total and Central Obesity Indices: NHANES 99-02. *Int J Environ Res Public Health*. 2010;7(9):3332-3347. doi:10.3390/ijerph7093332

51. Sabaredzovic A, Sakhi AK, Brantsæter AL, Thomsen C. Determination of 12 urinary phthalate metabolites in Norwegian pregnant women by core–shell high performance liquid chromatography with on-line solid-phase extraction, column switching and tandem mass spectrometry. *J Chromatogr B*. 2015;1002:343-352. doi:10.1016/j.jchromb.2015.08.040

52. Valvi D, Monfort N, Ventura R, et al. Variability and predictors of urinary phthalate metabolites in Spanish pregnant women. *Int J Hyg Environ Health*. 2015;218(2):220-231. doi:10.1016/j.ijheh.2014.11.003

53. Sakhi AK, Sabaredzovic A, Papadopoulou E, Cequier E, Thomsen C. Levels, variability and determinants of environmental phenols in pairs of Norwegian mothers and children. *Environ Int*. 2018;114:242-251. doi:10.1016/j.envint.2018.02.037

54. Philippat C, Mortamais M, Chevrier C, et al. Exposure to phthalates and phenols during pregnancy and offspring size at birth. *Environ Health Perspect*. 2012;120(3):464-470. doi:10.1289/ehp.1103634

55. Cequier E, Sakhi AK, Haug LS, Thomsen C. Development of an ion-pair liquid chromatography-high resolution mass spectrometry method for determination of organophosphate pesticide metabolites in large-scale biomonitoring studies. *J Chromatogr A*. 2016;1454:32-41. doi:10.1016/j.chroma.2016.05.067

56. Aurrekoetxea JJ, Murcia M, Rebagliato M, et al. Determinants of self-reported smoking and misclassification during pregnancy, and analysis of optimal cut-off points for urinary cotinine: a cross-sectional study. *BMJ Open*. 2013;3(1):e002034. doi:10.1136/bmjopen-2012-002034

57. Sunyer J, Garcia-Esteban R, Castilla AM, et al. Exposure to second-hand smoke and reproductive outcomes depending on maternal asthma. *Eur Respir J*. 2012;40(2):371-376. doi:10.1183/09031936.00091411

58. Eisenmann JC. On the use of a continuous metabolic syndrome score in pediatric research. *Cardiovasc Diabetol*. 2008;7(1):17. doi:10.1186/1475-2840-7-17

59. Rigby R, Stasinopoulos M, Heller G, Bastiani FD. Distributions for Modelling Location, Scale and Shape: Using GAMLSS in R. Published online November 14, 2017.

60. Barba G, Buck C, Bammann K, et al. Blood pressure reference values for European non-overweight school children: the IDEFICS study. *Int J Obes 2005*. 2014;38 Suppl 2:S48-56. doi:10.1038/ijo.2014.135

61. Ahrens W, Moreno LA, Mårild S, et al. Metabolic syndrome in young children: definitions and results of the IDEFICS study. *Int J Obes 2005*. 2014;38 Suppl 2:S4-14. doi:10.1038/ijo.2014.130

62. Stratakis N, V Conti D, Jin R, et al. Prenatal Exposure to Perfluoroalkyl Substances Associated With Increased Susceptibility to Liver Injury in Children. *Hepatol Baltim Md*. 2020;72(5):1758-1770. doi:10.1002/hep.31483

63. Jin Y, Hein MJ, Deddens JA, Hines CJ. Analysis of Lognormally Distributed Exposure Data with Repeated Measures and Values below the Limit of Detection Using SAS. *Ann Occup Hyg*. 2011;55(1):97-112. doi:10.1093/annhyg/meq061

64. Stuart EA, Azur M, Frangakis C, Leaf P. Multiple imputation with large data sets: a case study of the Children’s Mental Health Initiative. *Am J Epidemiol*. 2009;169(9):1133-1139. doi:10.1093/aje/kwp026

65. Little RJA. Regression with Missing X’s: A Review. *J Am Stat Assoc*. 1992;87(420):1227-1237. doi:10.1080/01621459.1992.10476282

66. Agier L, Portengen L, Chadeau-Hyam M, et al. A Systematic Comparison of Linear Regression–Based Statistical Methods to Assess Exposome-Health Associations. *Environ Health Perspect*. 2016;124(12):1848-1856. doi:10.1289/EHP172

67. Wang Z, Ma S, Zappitelli M, Parikh C, Wang CY, Devarajan P. Penalized count data regression with application to hospital stay after pediatric cardiac surgery. *Stat Methods Med Res*. 2016;25(6):2685-2703. doi:10.1177/0962280214530608

68. Li MX, Yeung JMY, Cherny SS, Sham PC. Evaluating the effective numbers of independent tests and significant p-value thresholds in commercial genotyping arrays and public imputation reference datasets. *Hum Genet*. 2012;131(5):747-756. doi:10.1007/s00439-011-1118-2

69. White IR, Royston P, Wood AM. Multiple imputation using chained equations: Issues and guidance for practice. *Stat Med*. 2011;30(4):377-399. doi:10.1002/sim.4067

70. De Henauw S, Michels N, Vyncke K, et al. Blood lipids among young children in Europe: results from the European IDEFICS study. *Int J Obes 2005*. 2014;38 Suppl 2:S67-75. doi:10.1038/ijo.2014.137

71. Nagy P, Kovacs E, Moreno LA, et al. Percentile reference values for anthropometric body composition indices in European children from the IDEFICS study. *Int J Obes 2005*. 2014;38 Suppl 2:S15-25. doi:10.1038/ijo.2014.131

72. Peplies J, Jiménez-Pavón D, Savva SC, et al. Percentiles of fasting serum insulin, glucose, HbA1c and HOMA-IR in pre-pubertal normal weight European children from the IDEFICS cohort. *Int J Obes 2005*. 2014;38 Suppl 2:S39-47. doi:10.1038/ijo.2014.134
